# Spontaneous speech enables scalable digital phenotyping of physical functional deficits in aging

**DOI:** 10.1038/s41514-026-00343-3

**Published:** 2026-02-28

**Authors:** Eloïse Da Cunha, Raphaël Zory, Frédéric Chorin, Valeria Manera, Auriane Gros

**Affiliations:** 1 Université Côte d’Azur, Speech and Language Pathology department of Nice, Faculty of Medicine, Nice, France; 2 Université Côte d’Azur, COBTEK Laboratory (Cognition Behaviour Technology Laboratory), Nice, France; 3 Université Côte d’Azur, Interdisciplinary Institute of Artificial Intelligence Côte d’Azur (3IA Côte d’Azur), Nice, Sophia Antipolis France; 4 Centre Hospitalier Universitaire de Nice, Nice, France; 5 Université Côte d’Azur, LAMHESS (Laboratoire Motricité Humaine Expertise Sport Santé), Nice, France; 6 Institut Universitaire de France (IUF), Paris, France

**Keywords:** Biomarkers, Health care, Medical research, Neuroscience

## Abstract

The rising global burden of pathological aging engenders an urgent need for accessible tools enabling early detection of physical decline, which significantly impacts quality of life and healthcare systems. We hypothesized that speech analysis could capture phenotype-specific signatures of physical deterioration through shared neuromuscular pathways, offering a novel approach to physical assessment. In this study, we employed machine learning to analyze multimodal speech features (acoustic, linguistic, temporal) derived from two 1-minute spontaneous emotional speech recordings obtained from 271 community-dwelling older adults (mean age: 77.3 ± 5.8 years). Our models classified physical functional deficits across ten critical domains: lower-limb strength, power, endurance, handgrip strength, flexibility, postural balance, gait speed, mobility, appendicular lean mass, and fatigue. Our ensemble approach achieved remarkable classification accuracy for each domain (mean AUC = 0.91 ± 0.04), with multimodal emotional task stacking enhancing detection for 80% of physical measures. Explainable AI (SHAP) analysis revealed distinct speech signatures for each deficit type, potentially reflecting specific pathophysiological mechanisms rather than demographic confounders. We identified three primary speech alteration clusters: lexico-syntactic simplification (decreased syntactic complexity), neuromotor-temporal slowing (diminished speech rate, increased pauses), and articulatory-spectral decline (spectral instability). This study supports the hypothesis that spontaneous speech serves as a comprehensive digital biomarker of multidimensional physical function in aging. Our approach pioneers speech analysis as a physical aging clock. This technology offers clinical-grade precision through accessible smartphone recordings, enabling domain-specific physiological mapping via interpretable biomarkers and scalable screening for precision geriatrics and underserved populations.

## Introduction

Global population aging presents a critical public health challenge, with individuals aged over 60 years projected to represent 25% of the world’s population by 2050^1,2^. This transition amplifies the burden of physical decline, a multisystem phenomenon rooted in the core biological hallmarks of aging including mitochondrial dysfunction, genomic instability, and progressive neuromuscular degeneration^3^. Clinically, these processes manifest as physical frailty: a state of diminished physiological reserve and heightened vulnerability to stressors that strongly predicts disability, institutionalization, and premature mortality^4,5^. Critically, emerging evidence indicates that physical frailty not only precedes but actively accelerates cognitive impairment through shared pathways like chronic inflammation and neuroendocrine dysregulation^6,7^. The reversibility of early-stage decline through targeted interventions underscores the need for accessible frailty detection tools^8,9^.

Current physical assessment paradigms face fundamental limitations. Performance-based tools like grip dynamometry and walk speed provide validated metrics but require specialized equipment, trained personnel, and dedicated clinical spaces. Crucially, these methods capture capacity at single timepoints rather than dynamic resilience trajectories during daily activities^10^, while population-level implementation remains impractical in low-resource settings^11^. Wearable sensors offer promise for continuous monitoring but suffer from poor adoption in frail elderly people due to cost, usability barriers, and data complexity^12^. Consequently, pre-frailty cases frequently remain undetected until acute decompensation occurs, missing critical windows for preventive intervention^13^.

Voice analysis has emerged as a promising approach for neurological phenotyping, where subtle alterations in acoustic features, linguistic patterns, and temporal dynamics enable early detection of cognitive disorders. Despite progress in vocal biomarkers for cognitive disorders, their relationship with physical function remains largely unexplored despite strong mechanistic plausibility^14^. Speech and mobility share common neuromuscular foundations such as respiratory and postural muscles contribute to both phonation and locomotion^15^. This overlap suggests that age-related sarcopenia may simultaneously weaken vocal intensity and physical capacities. Moreover, dual-task paradigms (such as walking while speaking) reveal competition for shared executive resources, highlighting the integration of motor and cognitive control^16^. Evidence from Parkinson’s disease further illustrates this link, with vocal alterations strongly correlating with motor impairments^17,18^. Preliminary studies in geriatric adults also suggest that specific vocal patterns may reflect physical frailty, reinforcing the hypothesis that speech encodes multidomain physical decline^19,20^. Therefore, this study aims to establish whether spontaneous speech can serve as an early non-invasive biomarker of physical aging. We hypothesize that machine learning-derived speech signatures encode specific mechanisms of physical decline in aging.

Here, we establish voice as a new biomarker of physical decline in aging through machine learning analysis of acoustic, linguistic, and temporal features derived from spontaneous speech in 271 geriatric adults. Our approach achieves accurate classification of deficits across ten critical physical domains, including lower-limb and grip strength, power, endurance, mobility, balance, flexibility, fatigue, body composition, and postural control. We further identify mechanistically grounded vocal signatures predicting specific physical or motor alterations. Crucially, this assessment facilitates scalable remote monitoring, detecting physical decline with one minute of speech recording only. Collectively, this work pioneers voice as a high-resolution window into physical and motor aging decline.

## Results

### Population demographics and phenotypic stratification

Our cohort comprised 271 geriatric adults (68.6% female, mean age 77.3 ± 5.8 years) stratified across ten physical domains and described in Table 1. Age significantly differentiated deficit groups in seven domains (*p* < 0.05): grip strength (Δ = +6.8 years), gait speed (Δ = +5.1 years), Lower-Limb power (Δ = +4.6 years), balance (Δ = +1.4 years), mobility (Δ = +4.9 years), fatigue (Δ = +3.0 years), and flexibility (Δ = +1.8 years). Three domains exhibited distinct pathophysiology: lean mass deficits showed no age difference but significantly lower weight (*p* < 0.001) and BMI (*p* < 0.001); muscular endurance and lower-limb strength demonstrated physical impairment without significant age or anthropometric differences. Sex differences consistently revealed greater male anthropometrics. All ten domains showed significant physical declines (*p* < 0.001) in the deficit group. Crucially, cognitive screening using the MMSE confirmed no significant differences between deficit and normal groups across all physical classifications (mean = 26.6 ± 3.0, range = 13-30, median = 27; all *p* > 0.05), supporting that the classified deficits are specific to physical function rather than reflecting underlying cognitive differences. Additionally, regarding the screening of psychiatric symptomatology risk, the cohort prevalence of reported low mood was 33.6% (91/271) and of loss of interest was 30.3% (82/271). There were no significant differences in the prevalence of these symptoms between the deficit and normal groups for any of the ten physical domains (all *p* > 0.05), as detailed in Supplementary Table 1.

**Table 1:** Demographic and clinical characteristics of participants stratified by classification group for each physical function task

LOWER-LIMB STRENGTH															
Classes	Norm						Deficit	Statistics (Man Whitney test)							
Population (*n*)	Global (*n* = 166)		Women (*n* = 116)		Men (*n* = 50)		Global (*n* = 105)	Women (*n* = 70)	Men (*n* = 35)	Δ women	Δ men				Δ global
	mean	SD	mean	SD	mean	SD	mean	SD	mean	SD	mean	SD	*p* value	*p* value	*p* value
Age	75,78	5,99	75,96	5,86	75,35	6,27	80,42	4,54	79,44	4,39	82,39	4,21	<0.001*	<0.001*	<0.001*
Size (cm)	164,84	6,96	160,89	4,56	174,00	4,68	163,18	68,32	158,86	5,06	171,83	5,25	0,32	0,13	0,23
Weight (kg)	66,21	10,39	61,70	8,46	76,68	11,16	7,35	9,58	64,17	8,77	76,61	7,84	0,12	0,69	0,06
BMI (kg/m²)	24,25	3,04	23,82	3,01	25,25	2,93	25,62	3,12	25,46	3,55	25,93	2,22	0,13	0,20	0,15
Maximum leg power (W)	209,82	61,19	172,90	33,63	295,47	70,09	128,80	30,41	110,21	18,08	166,53	29,18	<0.001*	<0.001*	<0.001*
MMSE	26,91	2,33	26,84	2,44	27,06	2,09	25,51	3,15	25,86	2,72	25,60	3,27	0,23	0,12	0,16
**LOWER LIMB MUSCULAR ENDURANCE**															
Population (*n*)	Global (*n* = 75)		Women (*n* = 50)		Men (*n* = 25)		Global (*n* = 196)	Women (*n* = 136)	Men (*n* = 60)	Δ women	Δ men				Δ global
	mean	SD	mean	SD	mean	SD	mean	SD	mean	SD	mean	SD	*p* value	*p* value	*p* value
Age	77,26	6,04	77,83	5,53	76,13	7,04	77,70	5,66	77,07	5,50	79,13	5,84	0,56	0,18	0,77
Size (cm)	163,81	7,34	159,76	5,50	171,92	6,32	164,34	7,03	160,26	4,51	173,60	4,43	0,75	0,34	0,70
Weight (kg)	65,94	9,47	62,36	8,42	73,10	8,98	67,44	10,36	62,73	8,73	78,13	9,41	0,58	0,34	0,25
BMI (kg/m²)	24,53	2,95	24,46	3,13	24,68	2,57	24,88	3,15	24,43	3,33	25,89	2,61	0,76	0,09	0,26
Leg muscular endurance (*J*)	106,87	6,03	106,99	6,72	106,63	4,72	85,96	5,49	86,42	5,00	84,92	6,52	<0.001*	<0.001*	<0.001*
MMSE	26,85	2,47	26,84	2,49	26,88	2,41	26,28	2,59	26,30	2,55	26,23	2,67	0,13	0,38	0,07
**GRIP STRENGTH**															
Population (*n*)	Global (*n* = 74)		Women (*n* = 46)		Men (*n* = 27)		Global (*n* = 197)	Women (*n* = 139)	Men (*n* = 58)	Δ women	Δ men				Δ global
	mean	SD	mean	SD	mean	SD	mean	SD	mean	SD	mean	SD	*p* value	*p* value	*p* value
Age	72,61	5,20	72,61	4,90	72,62	5,90	79,44	5,08	78,85	5,06	80,87	5,07	<0.001*	<0.001*	<0.001*
Size (cm)	168,23	6,32	164,91	5,10	174,00	5,26	162,68	6,83	158,50	4,27	172,69	4,87	0,06	0,32	0,09
Weight (kg)	68,81	10,37	63,74	7,97	77,63	10,23	66,36	10,10	62,25	8,92	76,20	9,59	0,63	0,63	0,31
BMI (kg/m²)	24,17	2,54	23,39	2,33	25,53	2,53	25,01	3,26	24,79	3,49	25,53	2,70	0,32	0,90	0,08
Non- dominant Grip (*N*)	216,96	55,75	178,96	22,96	281,70	45,02	134,25	37,85	114,56	24,47	181,43	40,06	<0.001*	<0.001*	<0.001*
Dominant grip (*N*)	236,04	63,46	185,70	21,22	321,81	38,18	141,75	38,79	122,58	25,41	187,69	46,58	<0.001*	<0.001*	<0.001*
mmse	27,38	1,75	27,04	2,08	27,96	1,09	26,09	2,85	26,28	2,73	25,71	3,09	0,31	0,21	0,30
**BALANCE**															
Population (*n*)	Global (*n* = 124)		Women (*n* = 85)		Men (*n* = 39)		Global (*n* = 147)	Women (*n* = 101)	Men (*n* = 46)	Δ women	Δ men				Δ global
	mean	SD	mean	SD	mean	SD	mean	SD	mean	SD	mean	SD	*p* value	*p* value	*p* value
Age	76,82	5,40	76,93	5,19	76,58	5,87	78,22	6,00	77,56	5,78	79,66	6,35	0,04*	0,03*	0,08*
Size (cm)	164,64	7,12	160,20	4,36	174,31	4,93	163,82	7,11	160,06	5,10	172,09	4,87	0,72	0,14	0,52
Weight (kg)	67,49	10,92	62,16	3,00	79,10	10,07	66,64	9,52	63,02	8,83	74,58	9,35	0,37	0,11	0,96
BMI (kg/m²)	24,76	2,93	24,21	3,47	25,96	2,56	24,80	3,25	24,63	3,47	25,17	2,75	0,36	0,40	0,69
Balance on left leg (s)	19,87	8,44	19,86	8,34	19,88	8,65	1,21	1,48	1,10	1,42	1,45	1,63	<0.001*	<0.001*	<0.001*
Balance on right leg (s)	21,47	8,44	20,75	8,13	23,04	8,67	1,99	2,52	2,15	2,83	1,65	1,86	<0.001*	<0.001*	<0.001*
Mean balance (s)	17,75	8,57	17,64	8,38	18,01	8,95	0,97	1,16	0,99	1,25	0,94	0,96	<0.001*	<0.001*	<0.001*
MMSE	26,70	2,40	26,46	2,50	26,23	8,95	26,20	2,71	26,41	2,62	25,74	2,93	0,78	0,42	0,71
**WALK SPEED**															
Population (*n*)	Global (*n* = 180)		Women (*n* = 121)		Men (*n* = 59)		Global (*n* = 91)	Women (*n* = 65)	Men (*n* = 26)	Δ women	Δ men				Δ global
	mean	SD	mean	SD	mean	SD	mean	SD	mean	SD	mean	SD	*p* value	*p* value	*p* value
Age	75,85	5,62	75,76	5,28	76,04	6,30	80,99	5,18	76,09	5,34	83,24	4,11	<0.001*	<0.001*	<0.001*
Size (cm)	164,97	7,05	160,72	4,44	173,68	5,23	162,67	7,22	162,02	5,31	171,81	4,16	0,33	0,14	0,44
Weight (kg)	67,06	10,34	61,77	7,99	77,92	11,10	66,96	9,73	64,23	9,95	73,79	6,18	0,27	0,28	0,63
BMI (kg/m²)	24,51	2,99	23,91	2,93	25,76	2,88	25,31	3,35	25,42	3,87	25,02	2,05	0,07	0,37	0,28
Gait speed (m/s)	1,12	0,22	1,12	0,22	1,11	0,22	0,83	0,22	0,85	0,23	0,96	0,20	0,01*	0,01*	0,02*
MMSE	26,73	2,41	26,61	2,45	26,97	2,28	25,89	2,84	26,47	2,73	26,19	3,09	0,41	0,17	0,19
**MOBILITY**															
Population (*n*)	Global (*n* = 38)		Women (*n* = 29)		Men (*n* = 9)		Global (*n* = 233)	Women (*n* = 157)	Men (*n* = 76)	Δ women	Δ men				Δ global
	mean	SD	mean	SD	mean	SD	mean	SD	mean	SD	mean	SD	*p* value	*p* value	*p* value
Age	73,32	4,27	73,50	4,30	72,74	4,14	78,27	5,67	77,97	5,45	78,90	6,08	<0.001*	0,02*	0,09
Size (cm)	163,53	5,40	161,38	4,60	170,44	5,16	164,30	7,41	159,89	4,80	173,42	4,95	0,16	0,16	0,16
Weight (kg)	62,16	6,47	59,93	6,00	69,33	3,56	67,82	10,59	63,13	9,09	77,52	10,14	0,16	0,08	0,11
BMI (kg/m²)	23,25	2,13	23,04	2,34	23,90	1,21	25,03	3,22	24,69	3,40	25,72	2,77	0,08	0,09	0,09
Walked distance (m)	523,21	43,45	504,03	29,42	585,00	42,22	361,86	83,36	361,87	76,90	361,84	96,70	<0.001*	<0.001*	<0.001*
Gait speed (m/s)	1,45	0,12	1,40	0,08	1,63	0,12	1,01	0,23	1,01	0,21	1,01	0,27	<0.001*	<0.001*	<0.001*
MMSE	27,79	1,27	27,72	1,50	28,00	1,11	27,22	2,69	26,21	2,79	26,24	2,73	0,31	0,17	0,11
**APPENDICULAR LEAN MASS (ALM)**															
Population (*n*)	Global (*n* = 163)		Women (*n* = 122)		Men (*n* = 41)		Global (*n* = 108)	Women (*n* = 64)	Men (*n* = 44)	Δ women	Δ men				Δ global
	mean	SD	mean	SD	mean	SD	mean	SD	mean	SD	mean	SD	*p* value	*p* value	*p* value
Age	76,85	5,67	76,85	5,41	76,87	6,45	78,67	5,78	78,09	5,69	79,53	5,79	0,24	0,07	0,08
Size (cm)	163,90	7,30	160,25	4,87	174,76	4,97	164,65	6,73	159,89	4,56	171,57	4,61	0,77	0,11	0,32
Weight (kg)	71,58	9,62	67,43	7,22	83,90	9,71	60,16	8,96	53,47	6,03	69,90	6,09	<0.001*	<0.001*	<0.001*
BMI (kg/m²)	26,58	2,79	26,31	2,86	27,41	2,45	22,06	2,22	20,87	1,78	23,78	2,12	<0.001*	<0.001*	<0.001*
LAM	6,69	0,70	6,42	0,56	7,50	0,45	5,50	0,65	5,07	0,33	6,14	0,65	<0.001*	<0.001**	<0.001
MMSE	26,58	2,55	26,49	2,57	26,85	2,44	76,28	7,63	26,31	2,56	26,02	2,70	0,73	0,28	0,42
**FATIGUE**															
Population (*n*)	Global (*n* = 142)		Women (*n* = 100)		Men (*n* = 42)		Global (*n* = 129)	Women (*n* = 86)	Men (*n* = 43)	Δ women	Δ men				Δ global
	mean	SD	mean	SD	mean	SD	mean	SD	mean	SD	mean	SD	*p* value	*p* value	*p* value
Age	76,17	5,57	76,01	5,00	76,56	6,94	79,13	5,42	78,75	5,68	79,89	4,90	<0.001*	0,05	0,05
Size (cm)	164,46	7,27	160,49	4,79	173,93	5,41	163,90	6,96	159,70	4,82	172,30	4,51	0,59	0,19	0,83
Weight (kg)	65,24	9,90	60,85	8,11	75,69	8,96	69,00	10,19	64,70	9,04	77,59	10,62	0,08	0,72	0,06
BMI (kg/m²)	24,03	2,88	23,62	2,94	25,00	2,51	25,61	3,22	25,38	3,44	26,05	2,76	0,09	0,18	0.10
Fatigability Scale Score	21,61	7,07	21,59	7,08	21,67	7,03	47,70	7,15	48,50	7,26	46,09	6,75	<0.001*	<0.001*	<0.001*
MMSE	26,84	2,29	26,72	2,43	27,12	1,95	26,00	2,82	26,13	2,65	25,74	3,25	0,15	0,28	0,07

Classes: Groups; Norm: Normal group; Deficit: Deficient group; Statistics (Mann-Whitney test): Statistical comparison (Mann-Whitney U test); Population (*n*): Participant count; Global: Overall cohort; Women: Female subgroup; Men: Male subgroup; Δ women: Women comparison *p* value; Δ men: Men comparison *p* value; Δ global: Overall comparison *p* value; BMI (kg/m²): Body Mass Index; Leg muscular endurance (J): Leg muscular endurance (Joules); *p* value: Statistical significance; *p* < 0.001 (statistically significant).

*MMSE* Mini-Mental State Examination, *mean* mean value, *SD* standard deviation.

### Base models achieve accurate classification of ten physical impairments

Our analysis reveals that speech biomarkers provide a robust, non-invasive method for assessing geriatric physical function across ten key domains. All best base-models significantly surpass random baselines (AUC > 0.85 vs. 0.50, *p* < 0.001). Notably, base-model architectures demonstrated hierarchical performance: tree-based ensembles (Random Forest (RF)/ Extreme Gradient Boosting (XGB)) consistently outperformed shallow classifiers (Support Vector Machine (SVM)/Logistic Regression (LR)) (Supplementary Tables 2–5). Conversely, stacking ensembles revealed an inverse hierarchy: LR or SVM emerged as the optimal meta-learners. All the performance metrics of the best base models and stacking models are presented in Table 2.

**Table 2:** Performance metrics of the best-performing models for each physical function classification

Lower-Limb Endurance	task	Av Acc	Mean cv	AUC	PR	CK	LL	Precision		Recall		F1-score	
								*D*	*N*	*D*	*N*	*D*	*N*
RF	POS	0.90	0.91	0.91	0.94	0.79	0.36	0.92	0.87	0.89	0.91	0.90	0.89
RF	NEG	0.88	0.90	0.92	0.90	0.75	0.36	0.86	0.89	0.86	0.89	0.86	0.89
LR	C	0.95	0.90	0.95	0.94	0.89	0.26	0.93	0.97	0.97	0.92	0.95	0.95
**Balance**	**task**	**Av Acc**	**Mean cv**	**AUC**	**PR**	**CK**	**LL**	**Precision**		**Recall**		**F1-score**	
								***D***	***N***	***D***	***N***	***D***	***N***
RF	POS	0.87	0.91	0.92	0.89	0.74	0.38	0.86	0.88	0.91	0.82	0.88	0.85
RF	NEG	0.87	0.93	0.92	0.90	0.74	0.38	0.86	0.88	0.91	0.82	0.88	0.85
LR	C	0.93	0.92	0.95	0.92	0.89	0.28	0.84	0.83	0.83	0.84	0.83	0.83
**Fatigue**	**task**	**Av Acc**	**Mean cv**	**AUC**	**PR**	**Ck**	**LL**	**Precision**		**Recall**		**F1-score**	
								***D***	***N***	***D***	***N***	***D***	***N***
XGB	POS	0.82	0.81	0.82	0.92	0.63	0.41	0.87	0.76	0.79	0.85	0.83	0.80
XGB	NEG	0.82	0.80	0.89	0.81	0.63	0.46	0.74	0.90	0.88	0.76	0.81	0.83
LR	C	0.71	0.78	0.81	0.87	0.72	0.35	0.73	0.69	0.70	0.73	0.72	0.71
**Lower-Limb strength**	**task**	**Av Acc**	**Mean cv**	**AUC**	**PR**	**Ck**	**LL**	**Precision**		**Recall**		**F1-score**	
								***D***	***N***	***D***	***N***	***D***	***N***
XGB	POS	0.71	0.75	0.87	0.88	0.65	0.42	0.81	0.75	0.67	0.86	0.67	0.74
XGB	NEG	0.71	0.77	0.82	0.80	0.62	0.42	0.74	0.68	0.67	0.75	0.70	0.71
LR	C	0.70	0.75	0.74	0.68	0.67	0.37	0.75	0.72	0.66	0.81	0.75	0.70
**Grip strength**	**task**	**Av Acc**	**Mean cv**	**AUC**	**PR**	**Ck**	**LL**	**Precision**		**Recall**		**F1-score**	
								***D***	***N***	***D***	***N***	***D***	***N***
RF	POS	0.95	0.97	0.97	0.98	0.90	0.25	0.97	0.93	0.94	0.96	0.95	0.94
RF	NEG	0.96	0.94	0.98	0.98	0.93	0.26	0.97	0.96	0.97	0.96	0.96	0.97
SVM	C+	0.90	0.95	0.94	0.96	0.91	0.16	0.98	0.97	0.89	0.95	0.92	0.94
**ALM**	**task**	**Av Acc**	**Mean cv**	**AUC**	**PR**	**Ck**	**LL**	**Precision**		**Recall**		**F1-score**	
								***D***	***N***	***D***	***N***	***D***	***N***
RF	POS	0.85	0,85	0.87	0.89	0.69	0.43	0.86	0.83	0.86	0.83	0.86	0.83
RF	NEG	0.83	0,84	0.92	0.91	0.67	0.39	0.88	0.79	0.81	0.87	0.84	0.83
LR	C	0.81	0.82	0.88	0.89	0.63	0.42	0.91	0.73	0.72	0.92	0.81	0.81
**Mobility**	**task**	**Av Acc**	**Mean cv**	**AUC**	**PR**	**Ck**	**LL**	**Precision**		**Recall**		**F1-score**	
								***D***	***N***	***D***	***N***	***D***	***N***
RF	POS	0.93	0,93	0.96	0.98	0.92	0.20	0.92	0.94	0.94	0.91	0.93	0.93
RF	NEG	0.89	0,88	0.94	0.90	0.77	0.34	0.91	0.86	0.86	0.91	0.89	0.89
XGB	C	0.94	0.98	0.98	0.98	0.87	0.20	0.89	0.99	0.99	0.87	0.94	0.93
**Lower-limb Power**	**task**	**Av Acc**	**Mean cv**	**AUC**	**PR**	**Ck**	**LL**	**Precision**		**Recall**		**F1-score**	
								***D***	***N***	***D***	***N***	***D***	***N***
RF	POS	0.79	0,82	0.82	0.85	0.57	0.39	0.87	0.74	0.66	0.91	0.75	0.81
XGB	NEG	0.81	0,91	0.90	0.90	0.61	0.44	0.81	0.80	0.79	0.82	0.80	0.81
XGB	C	0.85	0.81	0.88	0.89	0.70	0.37	0.96	0.79	0.73	0.97	0.83	0.87
**Flexibility**	**task**	**Av Acc**	**Mean cv**	**AUC**	**PR**	**Ck**	**LL**	**Precision**		**Recall**		**F1-score**	
								***D***	***N***	***D***	***N***	***D***	***N***
RF	POS	0.90	0,91	0.93	0.95	0.80	0.33	0.96	0.82	0.88	0.93	0.92	0.88
RF	NEG	0.83	0,85	0.86	0.93	0.63	0.36	0.79	0.85	0.73	0.88	0.76	0.87
LR	C	0.90	0.93	0.95	0.97	0.87	0.16	0.98	0.87	0.89	0.95	0.92	0.94
**Gait speed**	**task**	**Av Acc**	**Mean cv**	**AUC**	**PR**	**Ck**	**LL**	**Precision**		**Recall**		**F1-score**	
								***D***	***N***	***D***	***N***	***D***	***N***
RF	POS	0.90	0,92	0.99	0.98	0.80	0.35	0.88	0.94	0.95	0.85	0.91	0.89
XGB	NEG	0.92	0,90	0.98	0.97	0.83	0.36	0.92	0.91	0.92	0.91	0.92	0.91
XGB	C	0.87	0.93	0.93	0.92	0.86	0.27	0.98	0.88	0.87	0.98	0.93	0.93

Physical task assessed; task: Speech condition; Precision: Precision; Recall: Recall; F1-score: F1-score.

*Av Acc* average accuracy, *Mean cv* mean cross-validated AUC, *AUC* area under the ROC curve, *PR* area under the precision-recall curve, *Ck* Cohen’s Kappa coefficient, *LL* log loss, *D*, deficient class, *N* normal class, *RF* random forest, *LR* logistic regression, *POS* Positive speech condition, *NEG* Negative speech condition, *C* combined speech data (POS + NEG).

For lower-limb muscular strength, Friedman tests showed significant differences in POS(Positive speech condition) (*χ*² = 19.18, *p* = 0.00072) and NEG (Negative speech condition) (*χ*² = 18.33, *p* = 0.00107) speech. Post hoc Nemenyi ranked XGB significantly above RF (POS: *P*(XGB > RF) = 0.99; NEG: P(XGB > RF) = 0.99) and LR (POS: *P* = 0.27; NEG: *P* = 0.18), with equivalence to SVM. Bayesian analysis confirmed decisive superiority of XGB over LR (*P* = 0.99) and SVM (*P* = 0.98) in POS. XGB presented slight superiority with RF in NEG (*P* = 0.72) and better test parameters. XGB is thus the most reliable model across affective states.

In the positive condition of lower-limb muscular power, Friedman’s test (*χ*² = 17.74, *p* = 0.0014) and Nemenyi post hoc ranked RF significantly above SVM and XGB. Bayesian analysis confirmed this (P(RF > SVM) = 1.0, *P*(RF > XGB) = 0.998). In the negative condition, RF and XGB did not differ significantly (Bayesian *P*(XGB > RF) = 0.585). XGB had marginally higher AUC (0.90 vs 0.86) but similar other test parameters. Thus XGB has marginally superior performance than RF.

For lower-limb muscular endurance in positive condition, Friedman’s test (*χ*² = 19.11, *p* = 0.00075) and Nemenyi post hoc ranked RF and XGB above SVM (*p* < 0.01), with no difference between RF and XGB (*p* = 0.99). Bayesian analysis confirmed equivalence (*P*(XGB > RF) = 0.53). RF is preferred due to slightly better test parameters. In the negative condition, XGB significantly outperformed RF (*p* = 0.046) and all others (*p* < 0.03). Bayesian results (*P*(XGB > RF) = 0.96) and highest test parameters support XGB as optimal classifier for NEG.

For handgrip strength, Friedman tests revealed significant differences in POS (χ² = 19.84, *p* = 0.00054) and NEG (*χ*² = 17.38, *p* = 0.0016) speech. Post hoc Nemenyi ranked RF significantly above LR (POS: *p* = 0.18; NEG: *P* = 0.27) and marginally above SVM and XGB (POS and NEG: *P* ≈ 0.99). Bayesian analysis confirmed RF’s strong superiority over LR (POS: *P* = 0.98), equivalence with SVM and XGB (*P* = 0.81) in POS, and marginal preference over XGB (NEG: *P* = 0.52). RF consistently achieved higher test parameters, marking it as the most robust classifier.

RF consistently outperformed all classifiers for flexibility classification in both POS and NEG speech. Friedman tests showed significant differences (POS: *χ*² = 11.11, *p* = 0.025; NEG: *χ*² = 12.47, *p* = 0.014). Post hoc comparisons indicated RF’s significant superiority over LR and SVM, supported by strong Bayesian evidence (*P* > 0.93). Although XGB showed competitive results, RF achieved the best generalization (POS Cohen’s Kappa coefficient (CK) = 0.80; NEG CK = 0.73), confirming it as the most robust model for classifying flexibility across affective speech conditions.

For postural balance classification, Friedman’s tests indicated significant differences in both POS (χ²=17.28, *p* = 0.0017) and NEG (*χ*² = 19.35, *p* = 0.00067) speech conditions. Post hoc Nemenyi tests ranked RF significantly above LR (POS: *P*(RF > LR) = 0.988) and marginally above SVM (P(RF > SVM) = 0.75) in the POS condition. In the NEG condition, RF showed equivalence with SVM (*P*(RF > SVM) = 0.55) but was significantly better than XGB (*P*(RF > XGB) = 0.99). Bayesian analyses confirmed RF as the most consistent and optimal classifier across affective states, with high test AUCs (POS: 0.92, NEG: 0.91).

RF was the top-performing model for walk speed prediction across POS (Friedman *χ*² = 17.76, *p* = 0.0014) and NEG (Friedman χ²=19.18, *p* = 0.0007) speech. In POS, RF outperformed LR (*P*(RF > LR) = 0.97), XGB (*P*(RF > XGB) = 0.84) and SVM (*P*(RF > SVM) = 0.86). In NEG, RF maintained its lead with strong Bayesian evidence versus LR (*P*(RF > LR) = 0.98), XGB (*P*(RF > XGB) = 0.82) and SVM (*P*(RF > SVM) = 0.96). These findings confirm RF’s robustness and reliability for predicting walk speed from speech features under varying affective states.

RF consistently outperformed other classifiers for mobility prediction in both POS (AUC = 0.97, F1 = 0.89) and NEG (AUC = 0.94, F1 = 0.91) speech. Friedman tests showed significant model differences (POS: *χ*² = 16.63, *p* = 0.0023; NEG: *χ*² = 14.65, *p* = 0.0055). Post hoc analysis found RF significantly superior to LR and SVM, with Bayesian evidence showing better performance of RF than XGB (POS: *P* = 0.81; NEG: *P* = 0.83). According to test parameters comparison, RF was confirmed as the most robust classifier for mobility assessment across affective states.

For ALM (Appendicular Lean Mass), Friedman tests indicated significant differences in POS (*χ*² = 16.77, *p* = 0.0021) and NEG (*χ*² = 18.72, *p* = 0.0009) speech. Post hoc Nemenyi ranked RF significantly above LR and SVM (*P* > 0.88) and practically equivalent to XGB (POS: *P* = 0.66; NEG: *P* = 0.12). Bayesian analysis supported RF’s superiority over LR and SVM and test parameters confirmed it as the most reliable classifier for ALM detection.

For fatigue detection, Friedman tests showed significant differences in POS (*χ*² = 17.25, *p* = 0.0017) and NEG (*χ*² = 18.67, *p* = 0.0009) speech. Post hoc Nemenyi ranked XGB significantly above LR and SVM in POS (*P* > 0.92), and above LR and RF in NEG, with equivalence to RF (POS) or SVM (NEG). Bayesian analysis confirmed XGB’s superiority over LR and SVM in POS, and equivalence with RF and SVM in NEG. XGB thus stands as the most reliable classifier across affective states.

### Stacking models enhance classification for specific motor phenotypes

Stacking ensembles integrating complementary affective speech representations (POS/NEG) significantly improved phenotypic classification over single-view models in 80% of physical domains, with task-dependent efficacy confirmed through rigorous statistical benchmarking. Statistical analyses via Friedman-Nemenyi tests and Bayesian superiority probabilities revealed task-dependent efficacy, with stacking demonstrating significant improvements over single-view classifiers in six domains while showing limited gains in four others. In addition to improving overall classification performance compared to individual base models, the stacking models substantially reduced false negatives on the independent test set, eliminating them entirely for two clinically critical outcomes: gait speed and handgrip strength^5^. This significant reduction in missed positive cases enhances the preventive utility of the classifiers, ensuring more accurate identification of early frailty-related deficits.

Multimodal stacking ensembles significantly outperformed the best single-view models for lower-limb endurance (*P*(LR>single-view RF) = 96.4%), postural balance (*P*(LR>single-view RF) = 0.86), and muscular strength (*P*(LR>single-view XGB) = 80.7%). For grip strength, SVM stacking achieved superior performance (*P*(SVM>single-view RF) = 99.26%). Flexibility classification similarly benefited from LR-based stacking (*P*(LR>single-view RF) = 96.11%). Mobility stacking also showed improvement over single-view RF classifiers (*P*(XGB>single-view RF) = 89.26%).

Logistic regression emerged as the optimal meta-classifier for endurance, balance, strength, and flexibility tasks, consistently outperforming alternative stacking configurations in Bayesian pairwise comparisons (*P* > 85% in 8/10 domains). Exceptions included grip strength where SVM proved superior (*χ*² = 19.43, *p* = 0.00065) and mobility where RF/XGB ensembles exceeded LR performance (*P* > 90%), underscoring the critical importance of task-specific meta-learner selection.

For fatigue classification, single-view RF models substantially outperformed all multimodal ensembles (*P*(RF POS > LR meta-model) = 99.7%). Similarly, ALM and Lower-Limb power classification exhibited negligible gains over single-view benchmarks (Bayesian superiority probabilities < 77%), with RF/XGB models achieving statistically indistinguishable accuracy (AUC 0.82–0.89). In walking speed, combined RF/XGB stacking attained high performance (AUC 0.93) but failed to surpass single-view models (*P* < 0.17).

### Multidimensional vocal signatures of motor phenotypes revealed by interpretable machine learning

**Fig. 1:**
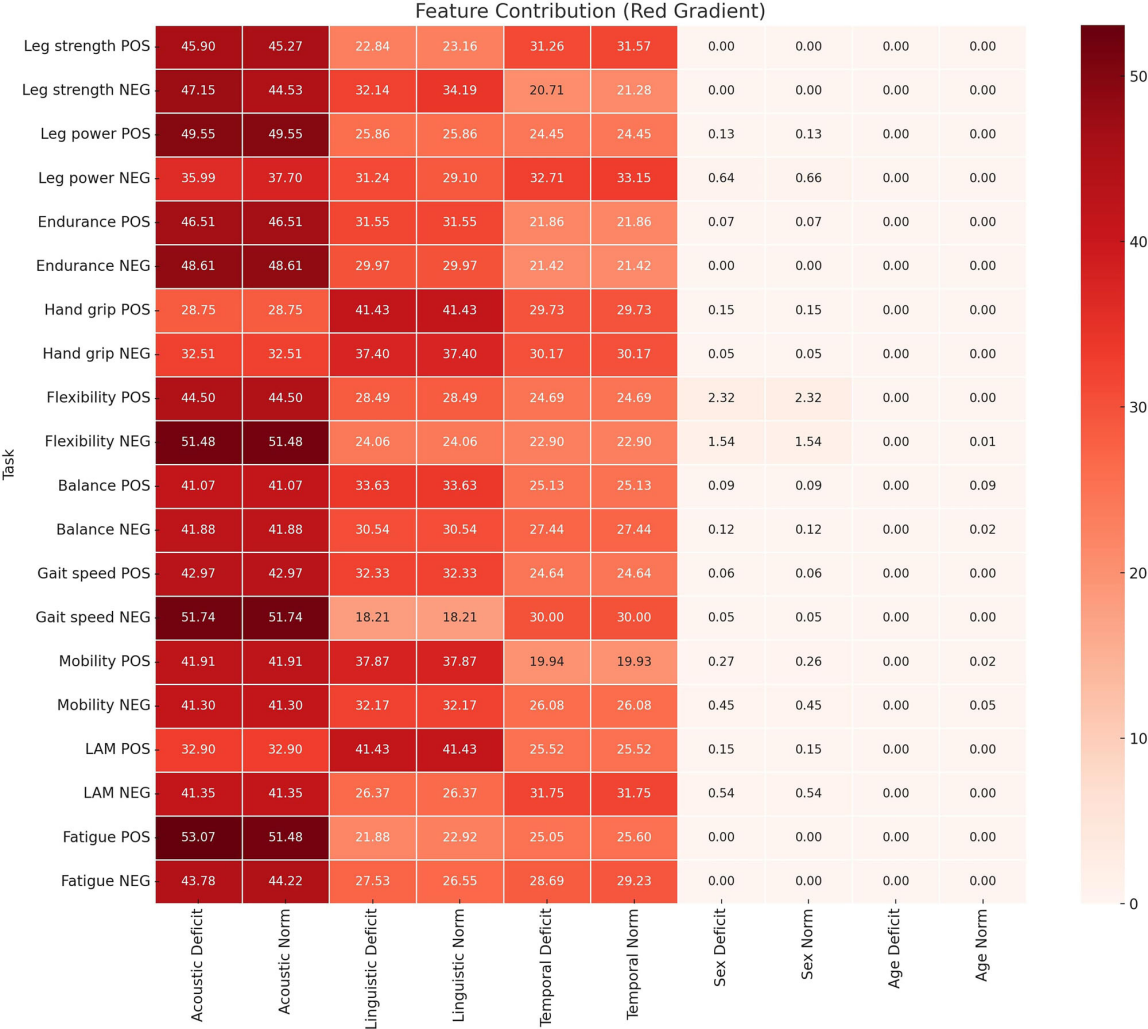
Relative contribution of acoustic, linguistic, temporal, and demographic features to the detection of physical function deficits. This heatmap displays the percentage contributions of different categories of features to the performance of classification models predicting physical function across multiple tasks. Each row corresponds to a specific binary classification task related to a physical function measure, separated by the task-trained model (POS = positive task, NEG = negative task). Columns represent the normalized contribution (in %) of distinct class labels categories used in the models : deficit or norm. Class categories labeled “Deficit” correspond to physical impairment; “Norm” refers to participants without impairment. The red color gradient reflects the relative importance of each feature group within each task, with darker shades indicating greater contribution. These results highlight distinct vocal signatures associated with specific types of physical functional deficits and underscore the proportional role of each marker type in model decision-making.

Interpretability analysis via SHAP (SHapley Additive exPlanations model) values illustrates how temporal, acoustic, and linguistic speech features appear to reflect phenotype-specific configurations across ten motor domains. These analyses seem to highlight triadic signatures that are consistent with distinct pathophysiological mechanisms underlying neuromuscular decline, as synthesized below. The feature importance hierarchy is presented in the Fig. 1; Analysis of feature importance proportions across ten motor domains reveals a consistent pattern: acoustic (28.75–53.07%) and temporal (19.93–33.15%) features constitute the primary discriminative signals. Linguistic features demonstrate variable but substantial contributions (18.21–41.43%). Notably, demographic factors show minimal influence: sex minimally contributes ≤2.3% (maximal in flexibility), while age effects are negligible ( ≤ 0.09%). This hierarchy confirms the robustness of these speech markers to demographic confounders.

**Fig. 2:**
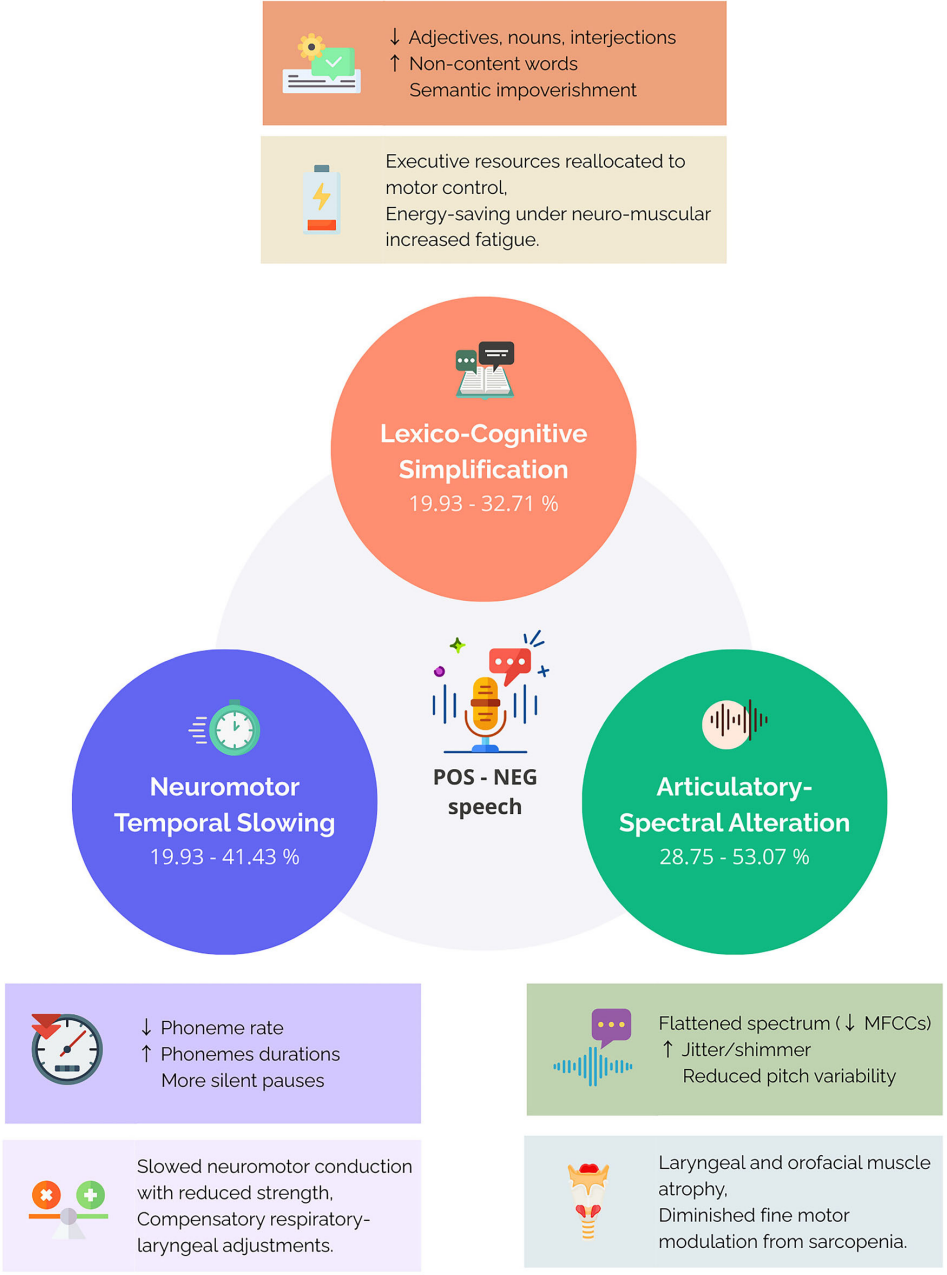
Hypothetical conclusions linking speech changes to physical function decline in aging. This conceptual model illustrates three dominant combinations of speech alterations associated with physical functional deficits in aging, as revealed by SHAP analyses conducted across 20 classification models. SHAP values quantified the contribution of each vocal feature to model decisions, enabling the identification of consistent patterns across tasks. Three main clusters of features emerged: lexico-cognitive simplification (e.g., reduced lexical diversity, simpler syntax), neuromotor and temporal slowing (e.g., slower speech rate, increased pauses, altered timing), and articulatory-spectral alteration (e.g., changes in formant frequencies, prosodic instability, reduced vocal energy). These combinations suggest distinct vocal signatures reflecting different physiological and cognitive mechanisms underlying physical function decline.

SHAP interpretation identifies distinct speech signatures across multiple physical domains. These results suggest potential associations and degradation pathways between speech and physical and motor functions summarized in Figs. 1, 2 and Table 3 and are detailed in Supplementary Fig. 1. The observed spectral and temporal compensations may be associated with locomotor muscle adaptations^21,22^. The observed acoustic instability (e.g., ↑shimmer) and articulatory slowing are consistent with the generalized neuromuscular decline of aging, which affects the laryngeal and respiratory systems essential for phonation^23^. Lower-limb muscular strength deficits feature motor instability ( ↑ shimmer)… accompanied by lexical simplification ( ↓ adjectives/nouns). This pattern mirrors the known age-related decline in both motor coordination and the retrieval of word forms, a process that relies on the efficient transmission of activation across phonological networks^23^. Lower-limb muscular power deficit classification exhibits a maintained lexical quality but a harmonic degradation (altered Fourier/pitch slope) and a temporal dysregulation ( ↑ fricative durations vs. accelerated phoneme/word rates). These modifications could also be interpreted as compensatory motor hyperactivity as indicated by the inverse relationship between temporal acceleration and spectral degradation. Such temporal and spectral dysregulation may be linked to broader age-related neural timing changes observed during language processing^24^. Lower-limb muscular endurance deficit manifests spectral fatigue ( ↓ MFCC2/5, Harmonics-to-Noise Ratio (HNR)) with compensatory articulatory adaptations ( ↑ plosive/fricative durations) and lexical restriction ( ↓ adjectives, ↑adverbs). A paradoxical POS profile with elevated pitch and reduced jitter, may be related to precise early compensation of presbyphonia, while NEG valence shows spectral degradation with Mel-Frequency Cepstral Coefficients (MFCC) 5/2/7 reduction consistent with energy management failure.

**Table 3:** SHAP-based ranking of the most impactful features from each domain across all physical function classification tasks

Motor Domain	Task	Dominant Temporal Features (rank)								Dominant Acoustic Features (rank)			Dominant Linguistic Features (rank)
Lower-Limb Strength	POS	↓ Longest silence (4) ↑ Word duration (5)				↓ Shimmer Local (1) ↑ Spectral contrast (2)							↑ Prepositions rate (19) ↓ Nouns rate (20)
	NEG	↑Silence duration (4) ↑Word duration (7)				↓ Pitch slope (1) ↓ Jitter Ppq5 (2)							↓ Adjectives ratio (5) ↓ Nouns ratio (6)
Lower-Limb Power	POS				↑ Words rate (5) ↓ Phonemes rate (3)					↓ 1st Fourier coefficient (1) ↓ Jitter Ppq5 (5)			↑ Pronouns ratio (13) ↓ Verbs rate (16)
	NEG				↑ Mean silence duration (1) ↓ Phonemes rate (2)					↓ Pitch slope (3) ↓ Jitter Ppq5 (5)			↓ Time references ratio (6) ↑ Pronouns ratio (9)
Muscular Endurance	POS	↓ Longest silence (1) ↑ Fricative duration (4)					↑ Mean pitch (2) ↓ MFCC2 (3)						↓ Adjective ratio (7) ↓ Narrator rate (8)
	NEG	↓ Word length (9) ↑ Word duration (15)					↓ MFCC5 (1) ↓ Jitter Local Absolute						↑ adverbs ratio (5) ↓ Adjectives ratio (12)
Grip Strength	POS			↑ Words rate (3) ↑ liquid duration (4)						↓ Pitch slope (8) ↓ 1st Fourier coefficient (11)			↓ Adjective rate (1) ↓ Name rate (2)
	NEG			↑ Words duration (5) ↑ Vowel duration (6)						↓ Pitch slope (2) ↓ Maximum intensity (10)			↓ Adjective rate (1) ↓ Adjectives ratio (3)
Flexibility	POS	↓ Longest word duration (4) ↓ Vowel duration (7)						↓ Mean Pitch (1) ↑ MFCC7 (2)					↑ Verbs rate (12) ↑ Positive emotion references (17)
	NEG	↓ Word duration (9) ↓ Longest word duration (10)						↓ Median Pitch (1) ↓ Mean Pitch (2)					↓ Interjections rate (13) ↓ Interjections ratio (16)
Postural Balance	POS	↓ Phoneme rate (3) ↓ Silence duration (12)								↓ MFCC6 (1) ↑ Zero crossing rate (2)			↑ Spatial references ratio (5) ↓ Positive emotion references ratio (6)
	NEG	↓ Word length (4) ↓ Liquid duration (5)								↓ MFCC5 (1) ↓ MFCC6 (3)			↑ Narrator references ratio (2) ↑ Time references ratio (15)
Walk Speed	POS	↑ Total silence duration (4) ↓ Speech duration (7)							↑ MFCC4 (2) ↑ MFCC5 (3)			↓ Negative emotion references (1) ↓ Adjectives rate (10)	
	NEG	↓ Speech duration (4) ↑ Silence total duration (9)							↑ Jitter Ppq5 (1) ↑ MFCC5 (2)			↑ Conjunctions ratio (34) ↓ Verbs ratio (35)	
Mobility	POS		↑ Longest word duration (3) ↑ Liquid duration (7)								↓ Minimum intensity (1) ↑ Spectral contrast (2)		↑ Pronouns rate (5) ↑ Adverbs rate (6)
	NEG		↑ Silence count (6) ↓ Speech duration (13)								↑ Spectral contrast (1) ↓ Minimum intensity (2)		↓ Time references ratio (4) ↑ Prepositions rate (5)
Lean Mass	POS	↓ Longest word duration (2) ↓ Phonemes rate (3)								↑ Jitter Ppq5 (1) ↓ Mean Pitch (2)			↑ Articles ratio (5) ↑ Articles rate (6)
	NEG	↓ Phonemes rate (1) ↑ Silence count (3)								↓ Maximum Pitch (2) ↑ Jitter Ppq5 (4)			↑ Prepositions ratio (5) ↓ Nouns ratio (9)
Fatigue	POS	↓ Silence count (3) ↓ Word rate (4)								↓ MFCC4 (1) ↑ Maximum Pitch (2)			↓ Positive emotion ratio (6) ↓ Interjections ratio (9)
	NEG	↑ Longest silence (2) ↓ Phonemes rate (3)								↓ MFCC4 (1) ↑ Pitch slope (4)			↑ Narrator rate (7) ↑ Pronoun ratio (14)

Task: Physical task assessed; Dominant Temporal Features (rank): Most important time-related features (importance ranking); Dominant Acoustic Features (rank): Most important sound-related features (importance ranking); Dominant Linguistic Features (rank): Most important language-related features (importance ranking).

*POS* Positive speech condition, *NEG* Negative speech condition, *↓* Decreased in deficit group, *↑* Increased in deficit group.

Motor control integration shows distinct speech-kinematic coupling. Handgrip strength deficit presents temporal slowing ( ↑ phoneme duration) and prosodic reduction ( ↓ pitch slope) alongside linguistic reorganization ( ↑ adjectives/nouns). These observed temporal slowing and prosodic reduction in grip strength deficits suggest a shared pathway with the neurobiological changes that affect the speed and fluidity of language production in healthy older adults^25^. Flexibility deficits exhibit spectral alterations (lower pitch and intensity with increased MFCC), disrupted temporal coordination (POS: prolonged words/pauses; NEG: truncated words/diminished periods) and attenuated contrast dynamics (increased jitter and low intensity). Postural balance features spectral impoverishment ( ↓ MFCC1/4/6) and linguistic simplification ( ↓ conjunctions, ↑pronouns), distinguished by stable phoneme rate despite articulation deficits ( ↑ shimmer/Zero Crossing Rate (ZCR)).

Mobility degradation reflects systemic neuromuscular compromise. Walk speed deficits manifest temporal fragmentation ( ↑ silences, ↓speech rate) with spectral flattening ( ↓ pitch contrast) and lexical depletion ( ↓ adjectives, ↑pronouns), preserving phoneme rate. Increased ZCR and shimmer are consistent with compromised motor-speech integration. Mobility impairments reveal respiratory-vocal compromise ( ↓ intensity modulation), generalized spectral erosion, and narrative reduction ( ↓ spatiotemporal markers), with motor-timing alterations ( ↑ plosive/liquid durations).

Systemic neuromuscular degradation emerges through vocal fold and energy management pathways reflected in endurance alterations. ALM deficits feature laryngeal instability potentially ( ↑ jitter/shimmer, ↓pitch) and speech motor slowing ( ↓ phoneme rate, ↑pauses), coupled with syntactic simplification ( ↓ nouns)^26^. These acoustic features are hallmark indicators of presbyphonia, which involves age-related vocal fold atrophy and reduced respiratory support^23^. The coupling with syntactic simplification further suggests a cognitive-linguistic adaptation to compensate for increased vocal effort. Elevated MFCC5 with shortened plosives also suggest articulatory precision weakness. Fatigue classification shows associations with speech flow disruption ( ↑ silences), spectral collapse ( ↓ MFCC4, ↑jitter/shimmer), and affective blunting ( ↓ interjections), patterns that seem to parallel neuromuscular exhaustion in NEG contexts.

Distinct multimodal speech signatures differentiate deficit types (e.g., stable phoneme rate in balance vs. slowing in grip strength). This speech metrics analysis presents a first understanding of links between physical decline and speech variations that necessitate further future validation.

## Discussion

Confirming our hypothesis, this study establishes spontaneous speech as a non-invasive biomarker of motor decline in aging, where machine learning-derived vocal signatures encode specific early mechanisms of physical deterioration. By leveraging multimodal speech analysis of acoustic, linguistic, and temporal features, we demonstrate robust classification across ten critical physical domains through an accessible, equipment-free methodology. This approach enables accessible population screening particularly through simple smartphone/tablet implementation, facilitating early detection of physical decline before adverse outcomes manifest^13^. This tool holds unique public health value for rural and low-resource settings by delivering a low-cost solution with minimal technical requirements and clinical practitioners training^11^. Indeed, longitudinal voice monitoring provides a scalable solution for tracking physical trajectories without hospital infrastructure. Beyond enabling precise monitoring, speech assessment also minimizes the burden of resource-intensive clinical evaluations for patients with limited physical reserve. In addition, the precision of our models (mean AUC = 0.91 ± 0.04) supports personalized intervention strategies aligned with precision gerontology principles. Deviations from individualized speech baselines would enable pre-symptomatic intervention. This therapeutic window allows the implementation of precision rehabilitation protocols which would improve physical robustness. The simplicity of a 1-min recording opens avenues for home-based screening in underserved or remote populations, with minimal technical training. This framework addresses urgent world public health challenges posed by aging populations by: Enabling decentralized monitoring through consumer-grade technology, preventing avoidable institutionalization through early intervention, optimizing resource allocation via automated risk stratification^2^. With the increase of the world population over 65 years estimated by 2050^1,2^, speech-based monitoring could reduce physical dependency through preemptive interventions targeting acoustic signatures of decline.

Indeed, speech signatures appear to quantify multidimensional physiological decline. Vocal signatures, specific to each physical phenotype, show convergence around three physiological principles as described in Fig. 2. Firstly, the relation observed between neuromotor and speech performance may manifest in a slowing of phonemic articulation, characteristic in the model orientation to deficits of endurance, power and strength. This phenomenon is particularly shown by the prolongation of consonants, potentially reflecting simultaneous movement slowing of the phonatory and peripheral muscles, in a mechanism analogous to that initially documented in Parkinson’s models^27,28^. However, while Parkinson’s disease involves degeneration of the central synchronization circuits, consonant lengthening associated with physical aging appear to represent a compensatory adaptation to neuromuscular constraints^29^. These temporal alterations are consistent with the general neurobiological slowing of processing speed and motor timing that occurs with age, which affects both speech and limb movements^30,31^. Then, speech rhythm alterations appear representative of physical adaptations rather than distinct pathological symptoms. These limitations appear linked to the modified articulatory process. Indeed, our results suggest that articulatory alterations are even more observable through spectral degradation analyses. The reduction in MFCCs is directly related to lean appendicular mass and motor strength deficit models classification (Table 3). The observed spectral decline and articulatory instability (e.g., increased jitter, shimmer, and MFCC alterations) align with the established pathophysiology of presbyphonia, which includes age-related vocal fold atrophy, reduced respiratory support, and weakening of laryngeal and articulatory muscles^32,33^. Spectral degradation and articulatory imprecisions have already been described as quantifiers of aging voice in correlation with tongue and lips strength and speed decrease^34^. Therefore, we can hypothesize that muscle aging evolves with spontaneous speech through movement slowness and spectral degradation. Speech rate and acoustic features mirror a speed and precision decline in physical domains.

Secondly, a potential reallocation of cognitive resources may manifest in characteristic linguistic simplification: the reduction of adjectives in grip strength deficits could suggest that executive functions might prioritize vocal motor stability at the expense of lexical enrichment. This phenomenon is consistent with an executive and energy economy pattern, where linguistic selection dynamically adjusts to the available physical reserve^35^. This cognitive-linguistic adaptation may reflect increased cognitive load demands required to compensate for underlying presbyphonic changes, consistent with age-related difficulties in lexical retrieval and syntactic processing that occur even in healthy aging^23,24^. Future analysis of narrative macrostructures could clarify how discourse organization reflects underlying energy allocation. Nonetheless, the combined tasks analysis suggests how emotional valence may modulate compensatory strategies. The differential patterns between positive and negative speech tasks could provide crucial clinical insights: we hypothesize that the negative condition, being more cognitively and emotionally demanding, might overwhelm available physiological resources, potentially unmasking more profound spectral and temporal degradation that could reflect neuromotor exhaustion. Conversely, the positive condition might facilitate better compensation, potentially making strategic linguistic simplification a more prominent biomarker of underlying physical strain. This interpretation is supported by models of emotional aging, such as the “positivity effect,” which posit that processing negative information can be more cognitively demanding for older adults^36^. This valence-dependent expression pattern is illustrated in fatigue and endurance classifications, where compensatory strategies (impoverished interjections and affective neutralization) are notable in positive tasks but appear less evident during negative tasks, where decreased vocal intensity and F0 decay may signal more direct neuromotor challenges.

Hence, our analysis reveals phenotype-specific vocal signatures reflecting distinct neuromuscular and cognitive mechanisms. Endurance and strength impairments were associated with slowed articulation and spectral degradation, consistent with motor unit loss and reduced respiratory-laryngeal coordination. Conversely, grip strength and fatigue were marked by lexical simplification and prosodic flattening, suggesting cognitive prioritization and energy-saving strategies. Then, speech measures appear associated to physical age, distinctly from chronological time, due to the dominant contribution of speech characteristics (acoustic/temporal/linguistic >20% variance per deficit) relative to minimal demographic effects (age/gender ≤2.3%) in the classification of physical deficits. Crucially, our models appear to capture the functional impact of presbyphonic changes, such as reduced respiratory capacity and laryngeal stability, which are themselves manifestations of systemic physiological decline^23,37^. This could position speech as a potential new physiological aging clock, alongside the epigenetic markers of aging already presented^38–40^. However, speech markers would have a unique advantage: real-time monitoring of domain-specific decline. This quantification of physical age through speech is consistent with recent advances in modeling aging using clinical data^41^. For example, the maintenance of phonemic rate in balance deficits contrasts with the significant slowing of grip strength classification, revealing differential neuromotor pathways that demographic models cannot discern. Our analysis therefore hypothesizes that physiological aging may follow unified pathways for different phenotypes that are reflected in speech patterns. This convergence, if validated, could position speech analysis as a promising tool for integrated phenotyping of physiological aging, where vocal biomarkers could potentially reflect physical rather than chronological decline.

While this study establishes vocal biomarkers as sensitive indicators of specific physical decline in aging, several methodological considerations must be mentioned. First, the interpretability of speech-based digital phenotyping requires careful consideration of potential cognitive and affective confounders, given the established sensitivity of voice to neuropsychiatric conditions^42^. In this cohort, MMSE scores showed no significant differences across physical deficit classifications, and our post-hoc analysis revealed no association between screening positive for psychological risk (ICOPE) and classification into any physical deficit group (Supplementary Table 1). This suggests that the captured vocal signatures reflect a somatic, physiologically-grounded dimension distinct from mood-related symptomatology. This aligns with evidence suggesting that physical frailty is a robust, independent predictor of poorer emotional health^36^. This comorbidity underscores the necessity, as demonstrated in our study, to account for affective state when interpreting vocal biomarkers of physical decline. Future studies incorporating comprehensive cognitive and affective batteries will enable the development of multimodal models. Such models could disentangle and concurrently quantify the distinct, yet often co-occurring, signatures of physical, cognitive, and psychiatric decline within a unified speech analysis framework, advancing toward an accessible and precise geriatric assessment. Second, the generalizability of our findings across different languages requires careful consideration. Our study was conducted exclusively with French speakers, and specific linguistic structures (e.g., syntax, phonology) may influence the extracted features, particularly those in the linguistic and certain temporal domains. While this specificity may pose an initial limitation for direct cross-linguistic application, emerging evidence suggests that fundamental acoustic markers of physiological decline may be conserved across languages. A recent study in a Mandarin-speaking population, despite stark linguistic differences, successfully identified vocal biomarkers of physical frailty, highlighting the potential universality of acoustic features such as jitter, shimmer, and spectral measures^20^. Therefore, we hypothesize that while language-specific models might yield the highest accuracy, the core physiological principles linking neuromotor decline to vocal acoustics are likely to be universal. Third, our binary classification framework simplifies the multidimensional continuum of aging decline, a limitation compounded by the absence of standardized clinical thresholds for age-related physical deficits. Recruitment challenges also yielded class imbalances that required synthetic minority oversampling, introducing potential spectral artifacts in underrepresented phenotypes. These constraints reflect the exploratory innovation inherent in developing the voice-based aging physical decline assessment.

To address these limitations, future research should prioritize three advances. First, implementing multimodal deep learning architectures would enhance phenotypic discrimination through joint modeling of acoustic-linguistic-temporal dynamics while enabling multiclass deficit staging. Second, expanding speech protocols to include combinatorial tasks such as spontaneous speech paired with cognitively loaded sentence repetition or phoneme maximum phonation time could better detect the interplay between motor execution and reduction of muscular capacities^20^. Third, establishing international cohorts through multicenter collaborations would validate universal biomarkers while reducing synthetic data dependencies through enhanced sampling diversity, directly addressing the linguistic generalizability question raised above. Thus, while methodological refinements remain necessary, vocal phenotyping represents a paradigm-shifting approach to continuous, non-invasive health monitoring across the disease spectrum. Beyond aging applications, these speech biomarkers also show translational potential for monitoring acquired physical pathologies. Preliminary evidence suggests utility in tracking acquired or neurodegenerative deficits neurorehabilitation trajectories^43^. By capturing subtle biomechanical alterations in speech, this non-invasive surveillance paradigm could transform physical assessment across healthcare settings while conserving patient and provider resources.

In conclusion, this work establishes voice analysis as an accessible, high-resolution representation of physiological aging. Notably, the minimal influence of chronological age and sex on model performance supports the potential of speech to serve as a physical aging clock, that is, as a dynamic indicator of physiological aging distinct from time-based metrics. This “clock” would provide real-time insights into individual health trajectories, enabling timely and personalized interventions before overt frailty or disability emerge. While further validation is needed across languages, cultures, and clinical settings, the scalability, sensitivity, and interpretability of speech-based phenotyping offer immediate opportunities to democratize precise geriatric assessment, personalize preventive strategies, and redefine how we monitor and understand aging.

## Methods

### Ethics and participants

All procedures were approved by the regional ethics committee and conducted in accordance with the Declaration of Helsinki and relevant French regulations. This study was approved by the *Comité de protection des personnes Est II* (2021-A02986-35) and the protocol was registered (NCT05343286 first submitted on 2022-04-22). All participants provided written informed consent. A total of 302 geriatric adults residing across the Alpes-Maritimes department in France were enrolled through the Geriatric Department of Nice University Hospital. Each participant provided written informed consent and completed a full single 90-minute multimodal physical evaluation and a speech recording protocol (Table 1). Eligible individuals were aged over 60 years old, registered with French social insurance, and able to provide informed consent. They had to speak French fluently. We did not include anyone with a neurocognitive disorder precluding consent, those under legal guardianship or curatorship (per Article L. 1121-16 of the French Public Health Code). We excluded anyone who did not complete all the evaluation steps (*n* = 31).

### Physical evaluation protocol

Participants completed a standardized multimodal assessment battery evaluating ten specific physical capacity domains: walk speed, lower-limb strength, grip strength, lower-limb power, lower-limb endurance, mobility, balance, flexibility, lean appendicular mass, and fatigue. This evaluation was completed by anthropometric measurements (height, weight, Body Mass Index (BMI)), speech recordings and cognitive test with Mini-Mental State Examination (MMSE)^44^. Cognitive assessment with the Mini-Mental State Examination was included specifically to characterize the cohort’s cognitive profile and verify that any identified physical functional deficits were not confounded by cognitive differences between groups. Potential risk of psychiatric symptoms were screened using the two-item psychological well-being scale from the WHO ICOPE tool^45^. Participants were asked the following yes/no questions: (1) *“During the last two weeks, have you felt down, depressed or hopeless?”* (*“Au cours des deux dernières semaines, avez-vous ressenti un sentiment de déprime ou de désespoir?”*) and (2) *“During the last two weeks, have you felt little interest or pleasure in doing things?”* (*“Au cours des deux dernières semaines, avez-vous ressenti une perte d’intérêt ou de plaisir à faire des choses?”*).The order of the ten physical tests, cognitive screening, and speech recordings was randomized for each participant to mitigate potential order effects, learning bias, or fatigue. Physical tests were assessed by an MSc-trained adapted physical activity specialist. Concurrently, questionnaires and speech recordings were conducted by a speech-language pathologist. All assessors received standardized protocol training.

During the assessment, bilateral handgrip strength was measured using a calibrated dynamometer with participants seated, elbow flexed at 90° and shoulder adducted. Maximal voluntary contraction of five seconds was performed for each hand^5,46^. The grip strength of the dominant hand was used for classification. Knee extensor and flexor function, including maximal strength, power output, and muscular endurance, was evaluated on a Biodex System 4 Pro isokinetic dynamometer^25^. Participants were stabilized on the device with the dynamometer’s axis aligned to the anatomical knee axis and the leg stabilized by straps. Following a standardized warm-up and familiarization at submaximal intensities (50%, 75%, 100% perceived effort), five concentric maximal repetitions were performed to determine peak torque (Nm) to measure the maximum strength. Then, an all-out endurance challenge of 35 consecutive concentric repetitions was completed to quantify total work (J) and mean power. Rest intervals were provided prior to and after each velocity condition. Walking endurance was characterized by the 6-Minute Walk Test (6MWT) over a 10 m out–and-back course instrumented with an OptoGait system^47,48^. Participants were instructed to cover as much distance as possible in six minutes without external assistance. Total distance (m) was extracted for analysis. Gait speed was assessed previously on the same 10 m course: two trials were performed and the fastest time was used for analyses^49^. Participants were instructed to walk at a self-selected comfortable walking speed, reflecting their habitual gait. Balance was assessed via single-leg stance on a firm surface with eyes open. Participants alternately lifted each foot and maintained the position for as long as possible, up to 60 s^50^. Flexibility of the posterior kinetic chain was assessed using a modified seated Schober test. With legs fully extended and knees straight, participants were instructed to reach forward and push a graduated sliding ruler placed between their feet. The forward excursion distance (in centimeters), ranging from –30 to +30 cm, provided an index of hamstring and lumbar spine flexibility. Perceived fatigue was quantified using the validated French version of the MFI(Multidimensional Fatigue Inventory)^51^. Each item was scored 1 to 5 and aggregated into five subscale scores (general, physical, and mental fatigue, reduced activity, and reduced motivation). The global final score was used for analyses. Appendicular lean mass (ALM) was assessed using dual-energy X-ray absorptiometry (DEXA), with participants positioned supine, arms resting alongside the body, and all metallic objects removed. ALM was calculated as the sum of lean mass in all four limbs and normalized to height squared to obtain the appendicular lean mass index (kg/m²)^52^.

The ten evaluated physical performances were dichotomized into “Norm” or “Deficit” classes based exclusively on physical performance thresholds with age and sex stratification. These physical thresholds were derived from international consensus guidelines^52,53^, large-scale population-based cohorts^47,48^, and manufacturer technical specifications (e.g. Biodex). A specific physical deficit was defined as performance falling 1 standard deviation below the age- and sex-adjusted normative mean, a cut-off commonly associated with a mild deficit with increased risk of adverse outcomes such as frailty, disability, and falls^8,54^. The normative values and calculation methods are detailed in Supplementary Table 6.

### Speech protocol

Two distinct speech recording tasks were conducted using a tablet in an isolated quiet room. The tasks consisted of emotional autobiographical speech. The selection of emotional speech tasks was motivated by their capacity to elicit ecologically valid spontaneous speech while minimizing cognitive constraints. Unlike highly structured tasks, autobiographical recall requires minimal instruction, is universally accessible across literacy and cognitive levels, and captures natural speech patterns under varying emotional contexts, enhancing clinical applicability for physical deficit screening. This approach further supports the scalability of voice-based monitoring, as it leverages conversational speech that could be seamlessly integrated into routine clinical interactions or remote monitoring platforms. Participants were instructed to speak for one minute about a positive and negative event that had occurred in their lives. For the negative speech task (NEG), participants were explicitly instructed: ‘Please speak for one minute about a negative event that occurred in your life’ For the positive speech task (POS), the instruction was: ‘Please speak for one minute about a positive event that occurred in your life’ (French version : *Je vous demande de parler pendant une minute de quelquechose de négatif/positif qui s’est passé dans votre vie*.) Participants were informed in advance of the emotional nature of the speech task and were free to decline or stop the task at any time without consequence. Audio capture began immediately after the instruction to promote spontaneous speech production. To ensure standardized feature extraction across all participants, the recording duration was fixed at 60 s. The microphone continued recording for the full duration, even during periods of participant silence, to provide a consistent and ecologically valid sample that includes natural hesitation and planning pauses. This allowed for the computation of robust temporal features (e.g., speech rate, pause patterns) which are central to our analysis. Recordings exceeding 60 s were truncated, while those shorter than 55 s were excluded from analysis to ensure data quality. No records lasted less than 55 s.

Then, speech features were extracted using a multi-step Python (version 3.12) pipeline integrating audio signal analysis, temporal alignment, and linguistic processing (Supplementary Table 7). Each of the two 60-second emotional speech recordings (positive and negative) was processed as a continuous file without manual segmentation, preserving the natural flow of spontaneous speech. The automated pipeline processed complete recordings through sequential stages: initial transcription using Whisper (version 3), followed by parallel processing along three complementary axes. Features were extracted separately for each emotional context recording, maintaining the distinct characteristics of each task while enabling comparative analysis. Acoustic features were extracted with the Librosa library (version 0.10.2), which allowed for the quantification of key properties of the audio. These features captured the prosodic and phonatory components of speech. Temporal features were derived using Montreal Forced Aligner (MFA; version 3.2.1), which enabled precise alignment between the audio waveform and the transcribed text. This alignment produced detailed timing information at the level of words and phonemes, making it possible to compute silence, phonemic and word temporal measurements. Linguistic features, including grammatical, syntactic and semantic data, were extracted using the natural language processing library spaCy (version 3.8.2). This allowed for a structured assessment of the linguistic content and complexity of each speech sample.

We hypothesized that this tripartite feature categorization would capture distinct pathophysiological pathways of physical functional decline^55^. Specifically, acoustic features such as jitter, shimmer, harmonic-to-noise ratio, and spectral measures have been shown to worsen with age due to laryngeal muscle atrophy, reduced respiratory support, and mucosal changes^32,33,56^, supporting the idea that they reflect physiological integrity of the vocal apparatus and are sensitive to decline in muscular strength and power. Temporal features, including speech rate, articulation rate, and pause duration, are well documented to decrease and become more fragmented in older adults^57^, consistent with neurobiological slowing of processing speed and impaired temporal predictive motor coding with greater slowing under unpredictable timing^30,31^. Finally, linguistic features such as lexical diversity, syntactic complexity, and semantic content may serve as markers of compensatory cognitive adaptation: under physical impairment, older speakers may simplify their language, both lexically and syntactically, to reduce cognitive load or stabilize communication, a phenomenon aligned with findings in dual-task speech-motor studies^55^ and systematic reviews of cognition–speech relations in aging, as well as with cognitive simplification operations observed in NLP research^58^. Moreover, voice biomarkers have already been linked to physical frailty in older adults, strengthening the conceptual bridge between vocal acoustics and overall physical functional decline^19^. This analytical framework allows us to test these specific mechanistic pathways and determine the relative contribution of each domain to classifying distinct physical deficit phenotypes.

All resulting features were compiled into a unified dataset for subsequent classification models, enabling testing of specific mechanistic pathways and determining the relative contribution of each domain to classifying distinct physical deficit phenotypes.

### Machine Learning methodology

All collected data were reviewed for consistency and completeness prior to analysis. Speech, cognitive, and physical performance datasets were integrated using unique anonymous participant identifiers. Only participants with complete datasets for all modalities were retained for machine learning analysis. Data storage and processing complied with the European General Data Protection Regulation (GDPR, EU 2016/679). Automated transcriptions generated by Whisper underwent rigorous clinical validation through a two-stage independent verification protocol. In the initial phase, MSc research speech therapy trainees produced manual transcriptions for 50 randomly selected recordings encompassing both positive and negative speech tasks, working without access to automated outputs. Subsequently, a certified MSc Speech-Language Pathologist (ED) conducted differential verification by systematically comparing Whisper outputs against these human-generated transcriptions. Accuracy was quantified using Word Error Rate (WER), calculated as the sum of insertions, deletions, and substitutions divided by total reference words. This process demonstrated Whisper model reliability with an overall WER of 1.7% (SD = 0.9%), while clinically significant discrepancies occurred in only 0.2% of cases. The implied inter-rater agreement exceeded 98%, confirming suitability for diagnostic applications in geriatric medicine.

**Fig. 3:**
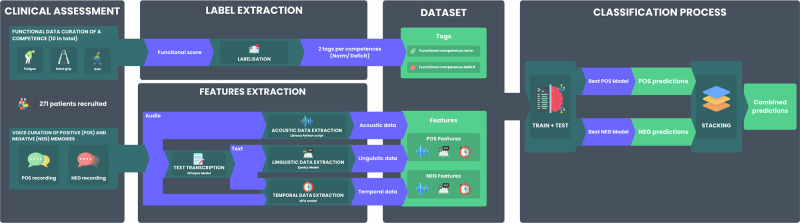
Overview of the methodological process for the development and evaluation of classification models for physical function deficits. This schematic illustrates the sequential steps used to construct and assess machine learning models for detecting physical function deficits from emotional speech recordings. The comprehensive workflow spans data preprocessing, feature engineering, base model development with explainability analysis, and stacking ensemble construction with rigorous validation. Detailed methodological schematics of each phase are provided in Supplementary Figs. 2.

All features were standardized (mean = 0, variance = 1) using Scikit-learn’s StandardScaler to ensure comparability across variables. The dataset was partitioned via participant-level stratified sampling, allocating 80% of participants to training and 20% to testing with a fixed random state (42) to ensure reproducibility. This participant-level split ensured that all recordings from the same individual were exclusively in either training or test sets, preventing data leakage. To prevent data leakage, standardization parameters were derived from the training set only before being applied to the test set. To address class imbalance, we implemented the Synthetic Minority Over-sampling Technique (SMOTE) with the following specific protocol: SMOTE was applied exclusively within each training fold during the 5 × 5 repeated cross-validation procedure, generating synthetic examples based on 5-nearest neighbors in the standardized feature space after acoustic, temporal and linguistic feature extraction. As detailed in Supplementary Table 1, we conducted comprehensive comparisons of SMOTE against class-weighting and no balancing approaches across all physical domains, confirming that feature space interpolation maintained discriminative patterns while effectively addressing class imbalance. SMOTE was never applied to the test set or during the final model evaluation. This approach ensured that synthetic data generation occurred only during model training while preserving the natural distribution of the independent test set for unbiased performance assessment. To verify the statistical power of the population for classification models development, the power analysis targeted an AUC of 0.80 Cohen’s transformation of AUC values in Python (statsmodels version 0.14.1). Even using the maximal class imbalance (Flexibility, Norm:Deficit = 1:2.93), statistical power exceeded 96% (*α* = 0.05). All the methodological process developed for the classification models is described in Fig. 3 and Supplementary Fig. 2.

We applied a supervised learning methodology using several classification algorithms : Logistic Regression (LR), Support Vector Machine (SVM), Random Forest (RF), and Extreme Gradient Boosting (XGB). These models were initially trained and evaluated separately on a dataset of positive task and then negative task features. As a point of reference, we included a random baseline model that produced predictions by randomly assigning class labels with uniform probability. To leverage complementary information from emotional speech tasks, we implemented a two-level stacking ensemble with rigorous safeguards against data leakage. The first level consisted of the best-performing POS and NEG base models for each physical domain, identified through our statistical benchmarking procedure. Their output prediction probabilities (for both ‘deficit’ and ‘norm’ classes) were processed using Platt scaling for SVM and isotonic regression for tree-based models to ensure probability calibration across different algorithm families. These calibrated probabilities were then combined to form the meta-feature matrix for the second level. We implemented a strict nested cross-validation approach where meta-features were generated using 5-fold cross-validation within the training set only. Specifically, for each of the 5 folds in the outer training set, base models were trained on 4 folds and generated predictions on the held-out fold. These out-of-fold predictions were concatenated to form the training meta-features, ensuring the meta-learner was never exposed to data used in base model training.Five meta-learners were evaluated : Random (performance baseline), RF, XGB, SVM, and LR. The optimal meta-learner was selected separately for each physical domain based on cross-validation performance within the training set.

Within the training set, a repeated stratified k-fold cross-validation procedure was implemented, comprising 5 repetitions of 5 folds each, ensuring robust performance estimation while maintaining the hierarchical structure of the data. This repeated cross-validation approach was specifically designed to account for variability in model performance across different data partitions. Hyperparameter optimization was conducted via randomized grid search nested within this cross-validation framework, with all optimization procedures strictly confined to the training folds to prevent any information leakage from the test set. Model performance was finally assessed by calculating the AUROC (Area Under the Receiver Operating Characteristic Curve) across the 25 iterations. Crucially, after hyperparameter tuning and model selection, a final evaluation was performed on the completely independent test set (20% of the participants) that was excluded from all previous steps including feature selection, hyperparameter optimization, and model training. Finally, after hyperparameter tuning, a final evaluation was performed on an independent test set that was not used during training or cross-validation. For each model was calculated : accuracy, AUROC, Ck (Cohen’s Kappa coefficient), LL (Logarithmic Loss), and precision, Recall and F1-score for each specific class (norm and deficit).

The same procedure was done for the stacking meta-model validation and test. The combined model demonstrating the best average AUROC performance during cross-validation was selected for final training on the full training set. It was subsequently evaluated on an independent test set using multiple metrics such as accuracy, AUC-ROC, LL, average precision, and Ck coefficient to ensure a robust and comprehensive assessment of predictive quality.

### Data analysis

Group differences in clinical and demographic characteristics were assessed between participants classified as “Norm” vs “Deficit” based on performance thresholds for each physical domain. Mann–Whitney U tests were used to compare continuous variables due to non-normal distributions. Analyses were conducted globally and stratified by sex. Results are presented as means and standard deviations, with *p*-values indicating between-group differences in Table 1. A significance threshold of *p* < 0.05 was applied. To assess and compare model performance across multiple metrics and tasks, we followed a two-stage approach combining frequentist and Bayesian statistical methodologies. We first applied the Friedman test to evaluate overall performance differences across experimental conditions, followed by post-hoc Nemenyi tests for pairwise comparisons, which control the family-wise error rate for multiple comparisons. To complement these frequentist approaches, we employed Bayesian correlated t-tests, which provide direct probabilistic interpretations of model superiority and allow for equivalence testing capabilities not afforded by standard null-hypothesis significance testing. We defined a Region of Practical Equivalence (ROPE) of ±0.01 for performance AUROC metrics bounded between 0 and 1, following conventions in the literature^59,60^. Probabilities were interpreted as follows: a model was considered superior if the posterior probability of its performance being better than another exceeded 0.95, and practically equivalent if the posterior mass within the ROPE exceeded 0.95. For statistically indistinguishable models (ROPE overlap >95%), we applied a pre-specified test set metric hierarchy: AUC, Ck, LL, accuracy. This analytic process flow prioritized discrimination robustness over raw classification rates. Principal performance metrics were reported with bootstrapped 95% confidence intervals in Supplementary Table 1, providing comprehensive uncertainty quantification for our model evaluations. In addition, we applied SHapley Additive exPlanations to the top-performing classifiers (POS, NEG, Stacking) to interpret feature contributions with analysis conducted on the independent test set. Different model-specific explainers were used according to the model architecture: TreeExplainer (XGB/RF), LinearExplainer (LR), and KernelExplainer (SVM). SHAP values were standardized across all features to compute percentage contributions of feature groups (acoustic, temporal, linguistic, demographic) to predictions detailed in Fig. 1.

## Data Availability

The de-identified data that support the findings of this study are available from the corresponding author upon reasonable request and subject to approval by the Ethics Committee, the relevant Comité de Protection des Personnes (CPP), in order to ensure compliance with participant confidentiality. All processed statistical datasets are provided in the Supplementary Table file. The underlying code for this study is not publicly available but can be made available to qualified researchers on reasonable request from the corresponding author.

## Appendix

Supplementary Figures
