# Supplementary material for: Spontaneous speech enables scalable digital phenotyping of physical functional deficits in aging
Source: NPJ Aging. 2026 Feb 28;12(1):52. doi: 10.1038/s41514-026-00343-3 (PMC13066492; doi:10.1038/s41514-026-00343-3)
Supplement: Supplementary file 1 — Supplementary Figures [file 41514_2026_343_MOESM1_ESM.pdf]

Supplementary Figures

Supplementary Figure 1 : SHAP summary plot: Top 10 predictive features for models deficit output 2

Supplementary Figure 2: Detailed Methodological Schematics of the Machine Learning Pipeline for Physical Deficit Classification from Emotional Speech 7

Supplementary Figure 2a: Data Preprocessing and Feature Engineering Pipeline 7

Supplementary Figure 2b: Base Model Development and Explainability Analysis 8

Supplementary Figure 2c: Stacking Ensemble Construction and Final Evaluation 9

Supplementary Figure 1 : SHAP summary plot: Top 10 predictive features for models deficit output

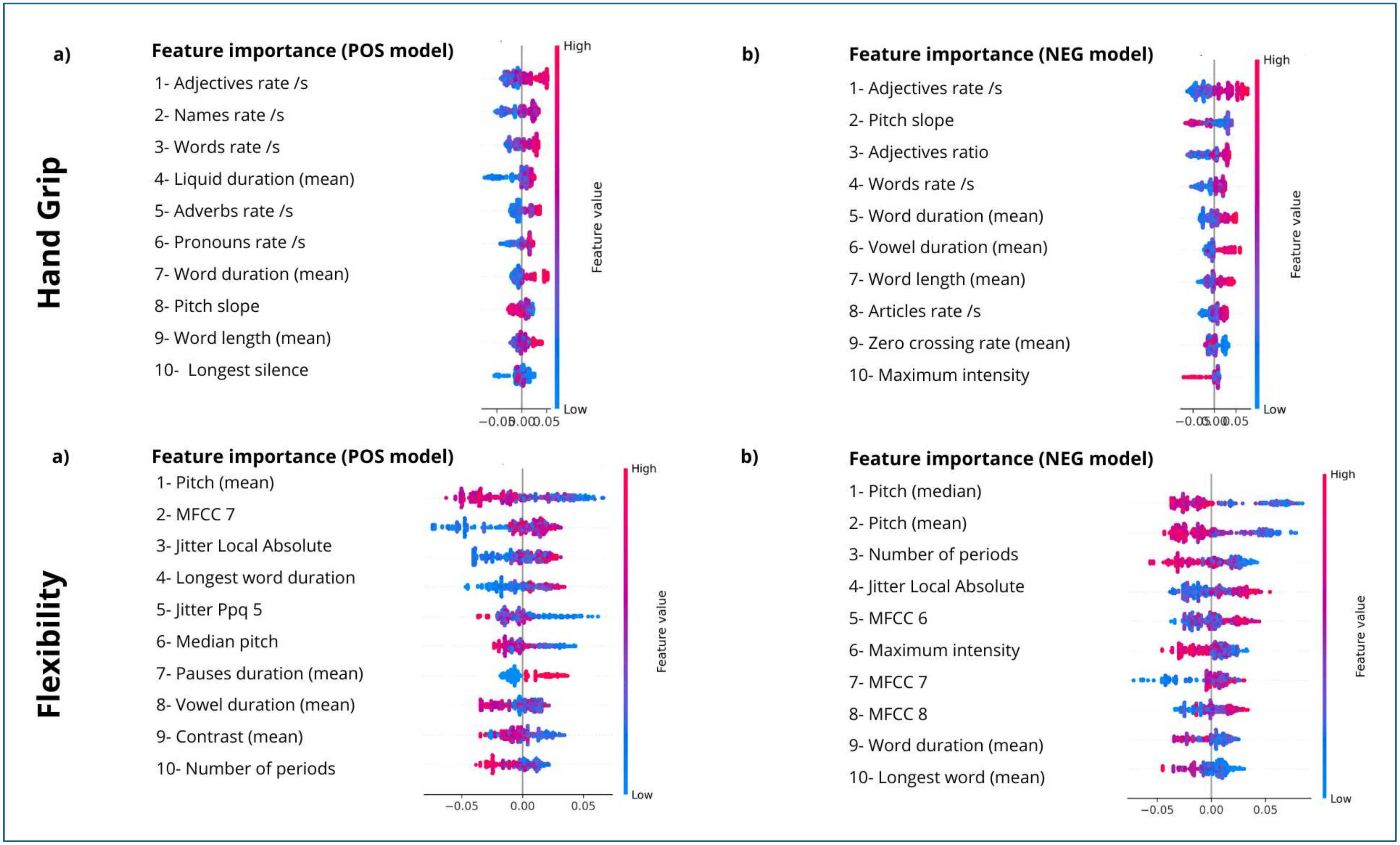

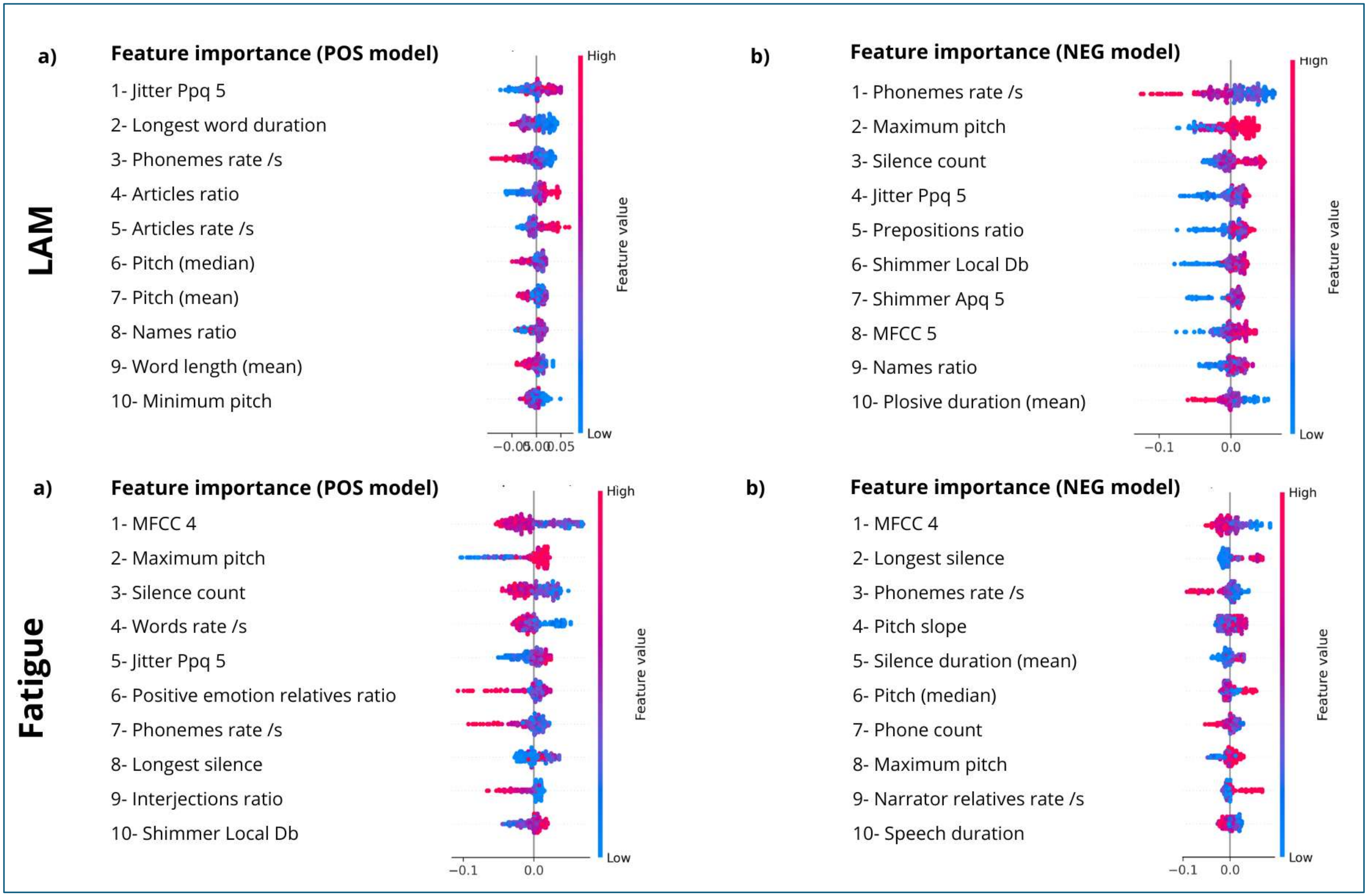
LAM
a)
Feature importance (POS model)
1- Jitter Ppq 5
2- Longest word duration
3- Phonemes rate /s
4- Articles ratio
5- Articles rate /s
6- Pitch (median)
7- Pitch (mean)
8- Names ratio
9- Word length (mean)
10- Minimum pitch
High
Low
Feature value
b)
Feature importance (NEG model)
1- Phonemes rate /s
2- Maximum pitch
3- Silence count
4- Jitter Ppq 5
5- Prepositions ratio
6- Shimmer Local Db
7- Shimmer Apq 5
8- MFCC 5
9- Names ratio
10- Plosive duration (mean)
High
Low
Feature value
−0.1
0.0
Fatigue
a)
Feature importance (POS model)
1- MFCC 4
2- Maximum pitch
3- Silence count
4- Words rate /s
5- Jitter Ppq 5
6- Positive emotion relatives ratio
7- Phonemes rate /s
8- Longest silence
9- Interjections ratio
10- Shimmer Local Db
High
Low
Feature value
−0.1
0.0
b)
Feature importance (NEG model)
1- MFCC 4
2- Longest silence
3- Phonemes rate /s
4- Pitch slope
5- Silence duration (mean)
6- Pitch (median)
7- Phone count
8- Maximum pitch
9- Narrator relatives rate /s
10- Speech duration
High
Low
Feature value
−0.1
0.0

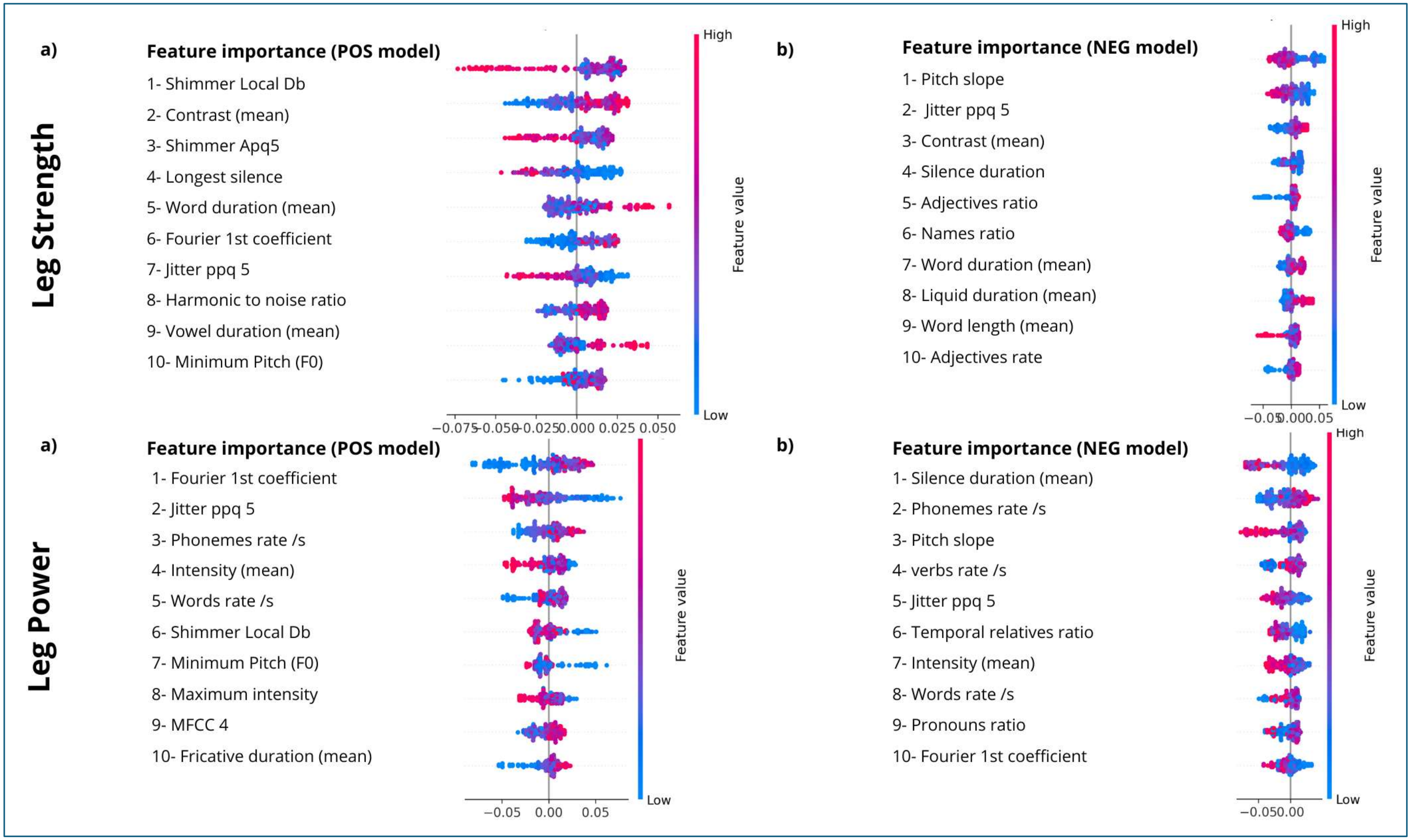

Leg Strength
a)
Feature importance (POS model)
1- Shimmer Local Db
2- Contrast (mean)
3- Shimmer Apq5
4- Longest silence
5- Word duration (mean)
6- Fourier 1st coefficient
7- Jitter ppq 5
8- Harmonic to noise ratio
9- Vowel duration (mean)
10- Minimum Pitch (F0)
High
Feature value
Low
b)
Feature importance (NEG model)
1- Pitch slope
2- Jitter ppq 5
3- Contrast (mean)
4- Silence duration
5- Adjectives ratio
6- Names ratio
7- Word duration (mean)
8- Liquid duration (mean)
9- Word length (mean)
10- Adjectives rate
High
Feature value
Low
Leg Power
a)
Feature importance (POS model)
1- Fourier 1st coefficient
2- Jitter ppq 5
3- Phonemes rate /s
4- Intensity (mean)
5- Words rate /s
6- Shimmer Local Db
7- Minimum Pitch (F0)
8- Maximum intensity
9- MFCC 4
10- Fricative duration (mean)
−0.05 0.00 0.05
High
Feature value
Low
b)
Feature importance (NEG model)
1- Silence duration (mean)
2- Phonemes rate /s
3- Pitch slope
4- verbs rate /s
5- Jitter ppq 5
6- Temporal relatives ratio
7- Intensity (mean)
8- Words rate /s
9- Pronouns ratio
10- Fourier 1st coefficient
High
Feature value
Low

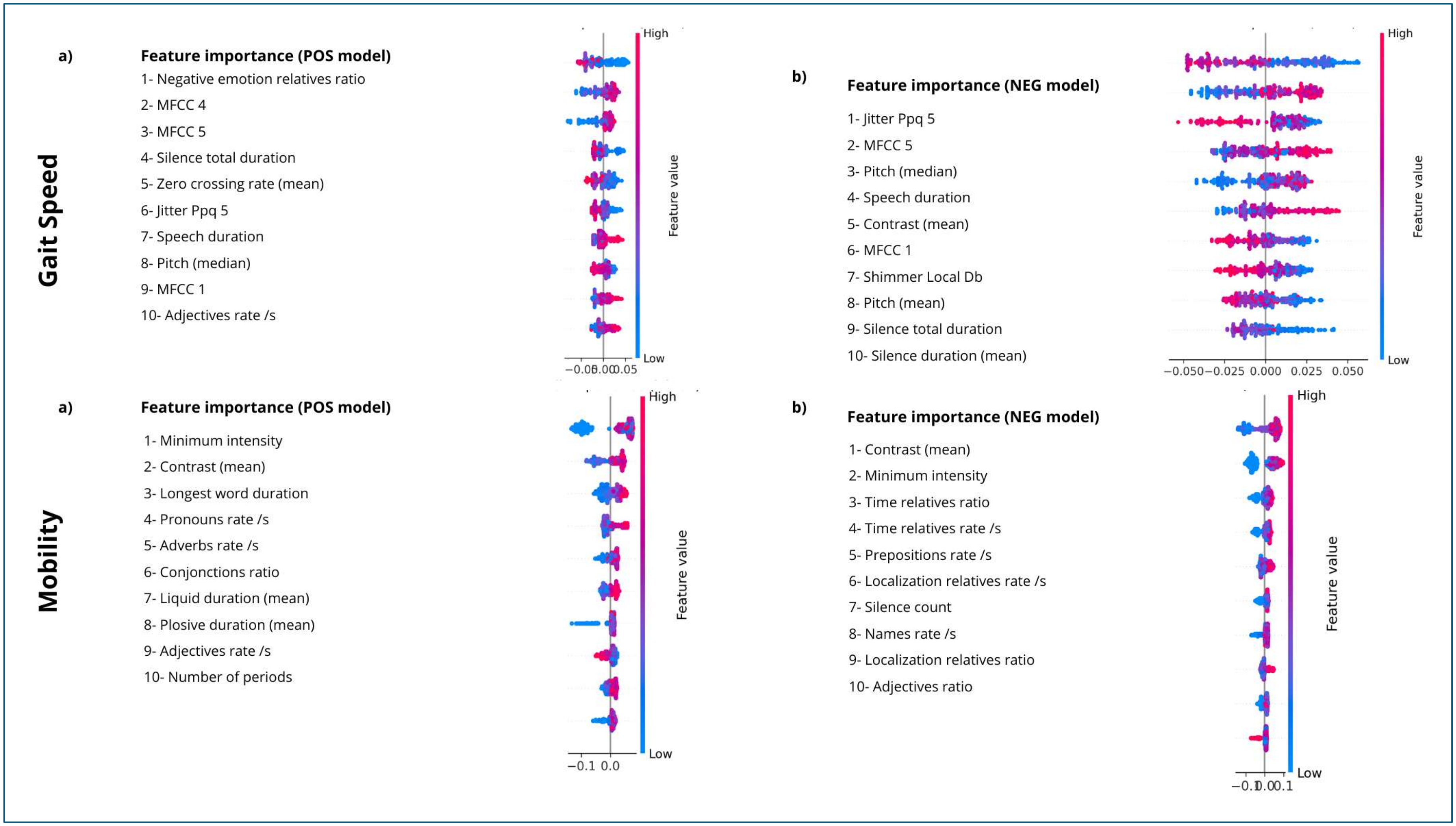
Gait Speed
a)
Feature importance (POS model)
1- Negative emotion relatives ratio
2- MFCC 4
3- MFCC 5
4- Silence total duration
5- Zero crossing rate (mean)
6- Jitter Ppq 5
7- Speech duration
8- Pitch (median)
9- MFCC 1
10- Adjectives rate /s
High
Feature value
Low
−0.05.000.05
b)
Feature importance (NEG model)
1- Jitter Ppq 5
2- MFCC 5
3- Pitch (median)
4- Speech duration
5- Contrast (mean)
6- MFCC 1
7- Shimmer Local Db
8- Pitch (mean)
9- Silence total duration
10- Silence duration (mean)
High
Feature value
Low
−0.050 −0.025 0.000 0.025 0.050
Mobility
a)
Feature importance (POS model)
1- Minimum intensity
2- Contrast (mean)
3- Longest word duration
4- Pronouns rate /s
5- Adverbs rate /s
6- Conjonctions ratio
7- Liquid duration (mean)
8- Plosive duration (mean)
9- Adjectives rate /s
10- Number of periods
High
Feature value
Low
−0.1 0.0
b)
Feature importance (NEG model)
1- Contrast (mean)
2- Minimum intensity
3- Time relatives ratio
4- Time relatives rate /s
5- Prepositions rate /s
6- Localization relatives rate /s
7- Silence count
8- Names rate /s
9- Localization relatives ratio
10- Adjectives ratio
High
Feature value
Low
−0.10.00.1

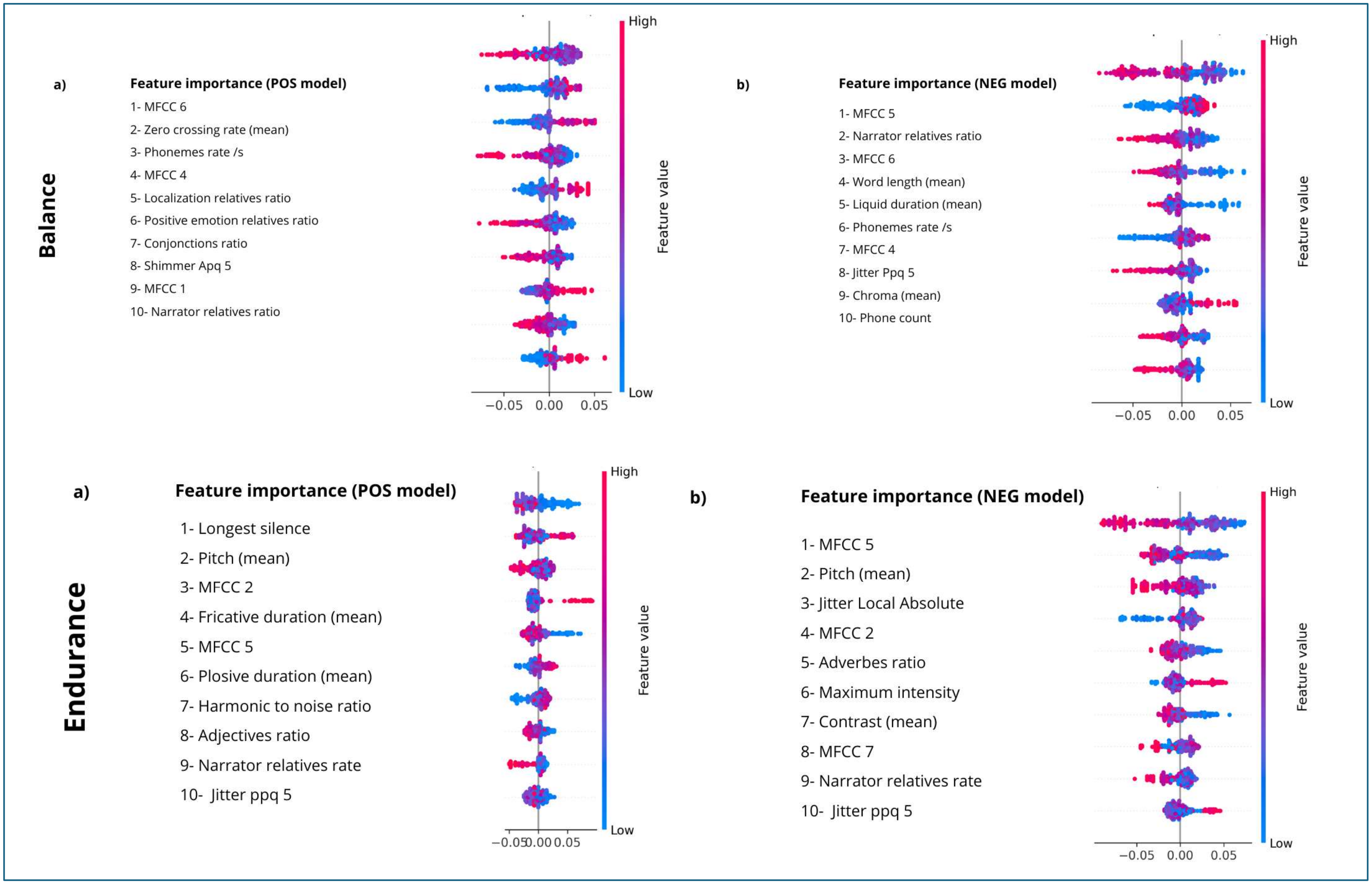

(a) best base Model for positive emotional speech (POS). (b) best base Model for negative emotional speech (NEG). For each model, features are ranked vertically by their mean absolute SHAP value. Each point represents a single participant. The horizontal position indicates the feature's effect on the model's output for the deficit class: a positive SHAP value (right side) means the feature value increased the predicted probability of a deficit for that participant, while a negative value (left side) decreased it. Color represents the original feature value from low (blue) to high (red). SHAP: Shapley Additive exPlanations.

Supplementary Figure 2: Detailed Methodological Schematics of the Machine Learning Pipeline for Physical Deficit Classification from Emotional Speech

Supplementary Figure 2a: Data Preprocessing and Feature Engineering Pipeline

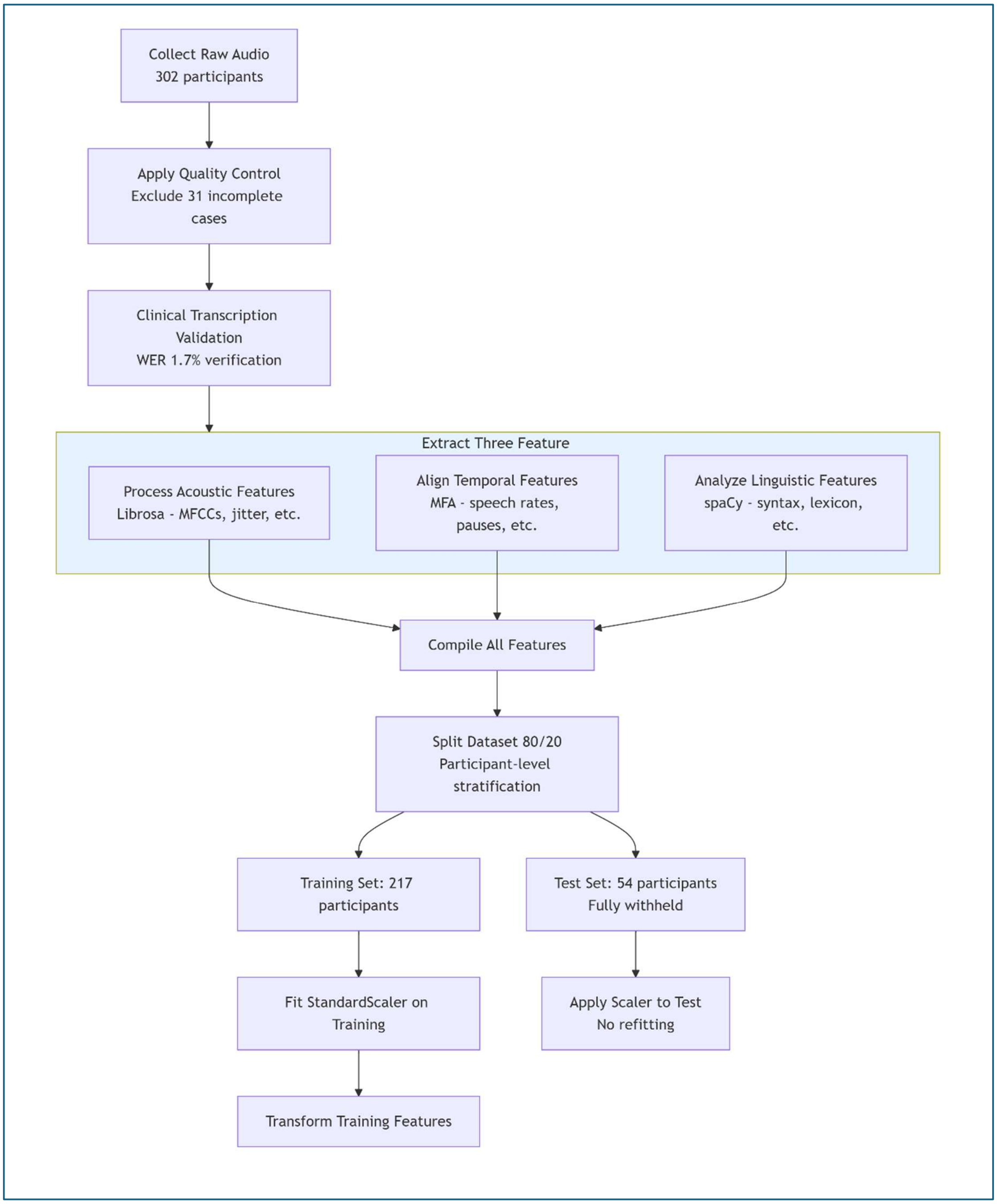

Detailed flowchart of the data preparation workflow from raw audio collection to standardized feature matrices, including quality control, tripartite feature extraction, and participant-level train-test splitting to prevent data leakage.

Supplementary Figure 2b: Base Model Development and Explainability Analysis

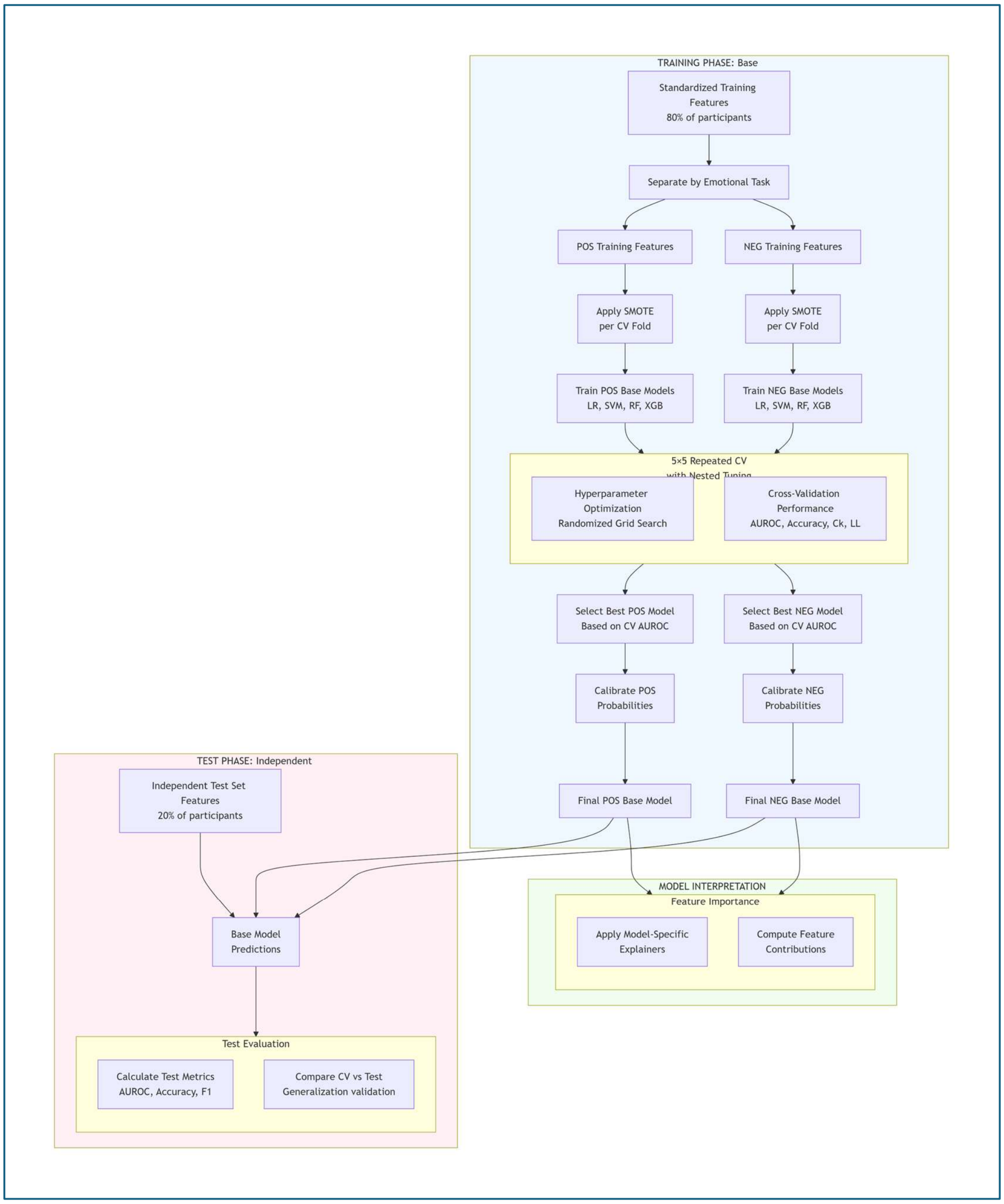

Schematic of the dual-task base modeling approach showing separate training for emotional speech tasks with repeated cross-validation, probability calibration, and SHAP-based interpretation using model-specific explainers.

Supplementary Figure 2c: Stacking Ensemble Construction and Final Evaluation

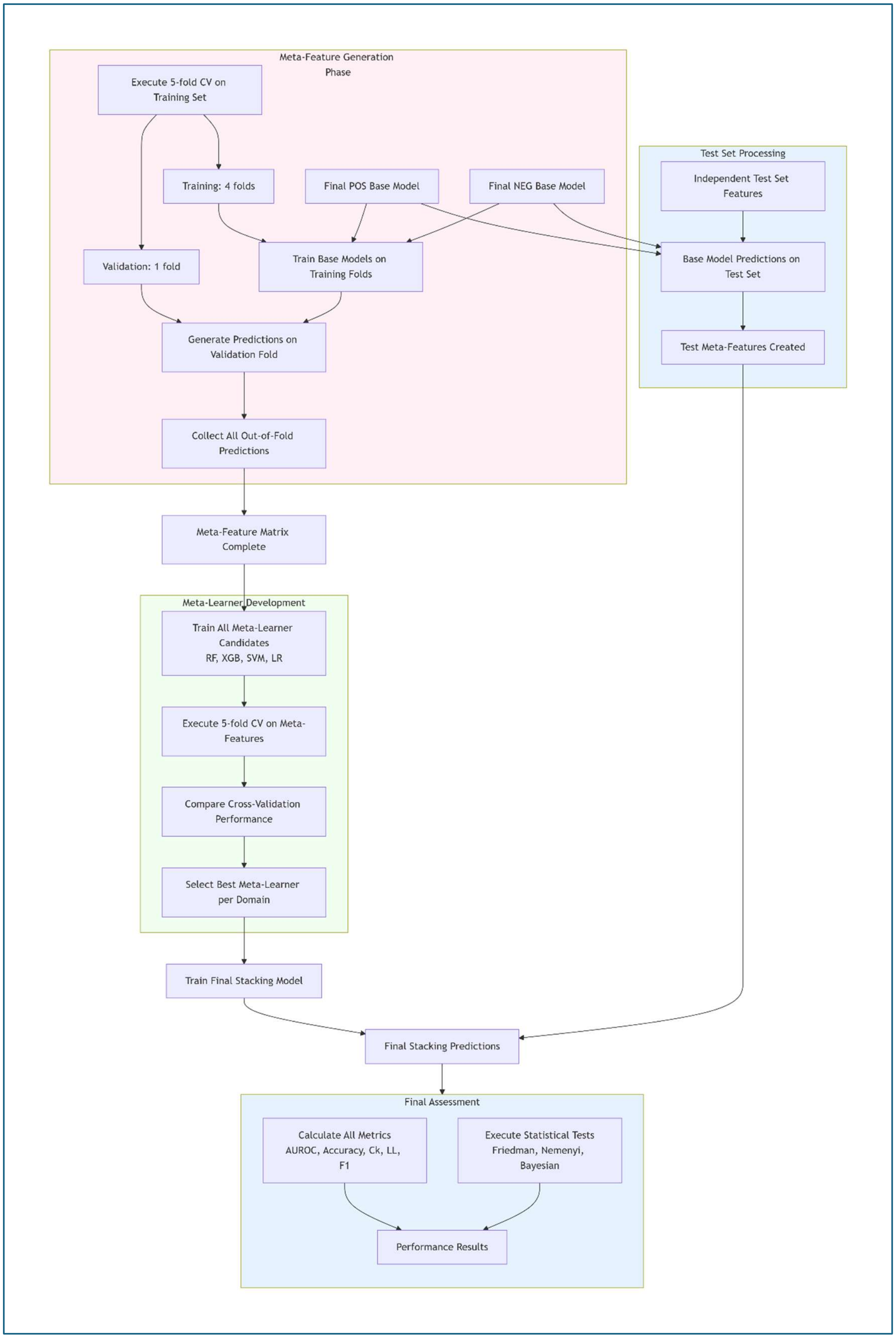

Workflow diagram of the two-level stacking ensemble methodology with strict nested cross-validation for meta-feature generation and comprehensive performance assessment on the independent test set.

Supplementary Tables

Supplementary Table 1 : Association between ICOPE psychological symptom screening and physical deficit classification 2
Supplementary Table 2 : Performance Metrics of Base Models for Physical Deficit Classification 3
Supplementary Table 3 : Synthesis of combined models parameters 7
Supplementary Table 4 : Bayesian comparisons of classification accuracies across Machine Learning models 9
Lower-Limb Endurance 9
Lower-limb strength 10
Lower-limb power 12
Grip strength 13
Walk speed 15
Mobility 17
Lean Appendicular Mass 18
Fatigue 20
Flexibility 21
Postural Balance 23
Supplementary Table 5 : Post Hoc pairwise comparisons of model accuracies (Nemenyi Test) 25
Lower-Limb Endurance 25
Lower-Limb Power 25
Lower-Limb Strength 26
Grip Strength 26
Walk speed 27
Mobility 27
Lean Appendicular Mass 27
Fatigue 28
Flexibility 28
Postural Balance 29
Supplementary Table 6 : Normative ranges and impairment thresholds for physical function measures 30
References 31
Supplementary Table 7 : Acoustic (a), Linguistic (b), and Temporal (c) speech markers with related extraction parameters 32
Acoustic Features 32
Temporal Features 32
Linguistic Features 33

Supplementary Table 1 : Association between ICOPE psychological symptom screening and physical deficit classification

Physical Domain	ICOPE Item	% Positive in Deficit	% Positive in Norm	p-value	Effect size (φ)
**Lower-Limb Endurance**	Feeling Depressed	26.6%	30.4%	0.625	φ=0.030
**Lower-Limb Endurance**	Loss of Interest	22.4%	29.1%	0.310	φ=0.062
**Balance**	Feeling Depressed	26.6%	30.6%	0.629	φ=0.029
**Balance**	Loss of Interest	25.1%	22.2%	0.740	φ=0.020
**Fatigue**	Feeling Depressed	29.8%	26.0%	0.583	φ=0.033
**Fatigue**	Loss of Interest	29.8%	20.0%	0.086	φ=0.104
**Lower-Limb Strength**	Feeling Depressed	27.3%	28.1%	0.984	φ=0.001
**Lower-Limb Strength**	Loss of Interest	22.4%	26.6%	0.510	φ=0.040
**Grip Strength**	Feeling Depressed	28.0%	25.6%	0.910	φ=0.007
**Grip Strength**	Loss of Interest	24.6%	23.1%	1.000	φ=0.000
**Lean Mass**	Feeling Depressed	29.6%	26.4%	0.655	φ=0.027
**Lean Mass**	Loss of Interest	27.8%	22.1%	0.355	φ=0.056
**Mobility**	Feeling Depressed	25.2%	23.2%	0.375	φ=0.013
**Mobility**	Loss of Interest	24.8%	21.6%	0.833	φ=0.013
**Lower-Limb Power**	Feeling Depressed	28.8%	26.9%	0.841	φ=0.012
**Lower-Limb Power**	Loss of Interest	21.2%	26.3%	0.410	φ=0.050
**Flexibility**	Feeling Depressed	25.6%	33.3%	0.289	φ=0.065
**Flexibility**	Loss of Interest	22.2%	31.8%	0.156	φ=0.086
**Gait Speed**	Feeling Depressed	29.3%	26.8%	0.766	φ=0.018
**Gait Speed**	Loss of Interest	26.1%	23.5%	0.744	φ=0.020

The table presents the statistical analysis of the association between positive screening for depressive symptoms (low mood and loss of interest, via the WHO ICOPE tool) and classification into the physical deficit group for each of the ten functional domains. For each domain and ICOPE item, the percentage of participants screening positive within the deficit and normal groups is shown. Associations were tested using Chi-square tests of independence. The phi coefficient (φ) is reported as the standard measure of effect size, where values approaching 0 indicate no association. All p-values are two-sided. None of the associations reached statistical significance at the $p < 0.05$ threshold.

Supplementary Table 2 : Performance Metrics of Base Models for Physical Deficit Classification

Model	Method	Acc	Acc_std	Accuracy_CI	AUC	AUC_std	AUC_CI	Av_Precision	CV_AUC_Mean	CV_AUC_Std	CV_AUC_Variance
**Lower-Limb Endurance NEG**	**oversample**	**0,88**	**0,03**	**[0,8125, 0,9375]**	**0,92**	**0,04**	**[0,8382, 0,9737]**	**0,87**	**0,91**	**0,03**	**0,00**
	classweight	0,76	0,06	[0,7026, 0,8088]	0,79	0,08	[0,7140, 0,8234]	0,72	0,87	0,07	0,01
	no_balancing	0,76	0,06	[0,6882, 0,7941]	0,76	0,08	[0,7050, 0,8032]	0,79	0,87	0,09	0,01
**Lower-Limb Endurance POS**	**oversample**	**0,90**	**0,03**	**[0,8438, 0,9583]**	**0,91**	**0,04**	**[0,8313, 0,9719]**	**0,87**	**0,91**	**0,03**	**0,00**
	classweight	0,79	0,06	[0,6882, 0,8088]	0,76	0,07	[0,6722, 0,8457]	0,80	0,77	0,02	0,00
	no_balancing	0,73	0,06	[0,7147, 0,8353]	0,79	0,08	[0,7462, 0,8395]	0,74	0,73	0,03	0,00
**Balance NEG**	**oversample**	**0,87**	**0,03**	**[0,8000, 0,9300]**	**0,92**	**0,03**	**[0,8540, 0,9722]**	**0,91**	**0,93**	**0,03**	**0,00**
	classweight	0,74	0,05	[0,6324, 0,8382]	0,70	0,07	[0,6635, 0,7535]	0,32	0,47	0,09	0,01
	no_balancing	0,72	0,05	[0,6176, 0,8235]	0,80	0,08	[0,7647, 0,8651]	0,33	0,54	0,12	0,01
**Balance POS**	**oversample**	**0,87**	**0,03**	**[0,8000, 0,9300]**	**0,92**	**0,04**	**[0,8310, 0,9779]**	**0,94**	**0,91**	**0,03**	**0,00**
	classweight	0,74	0,05	[0,6324, 0,8382]	0,79	0,09	[0,6990, 0,7536]	0,81	0,77	0,05	0,00
	no_balancing	0,72	0,05	[0,6176, 0,8235]	0,79	0,08	[0,7175, 0,8376]	0,76	0,73	0,12	0,02
**Fatigue NEG**	**oversample**	**0,82**	**0,06**	**[0,7262, 0,8885]**	**0,89**	**0,07**	**[0,8182, 0,9028]**	**0,84**	**0,80**	**0,08**	**0,01**
	classweight	0,76	0,06	[0,7235, 0,7735]	0,74	0,07	[0,7038, 0,7791]	0,72	0,80	0,10	0,01

	no_balancing	0,77	0,06	[0,7559, 0,7912]	0,78	0,07	[0,7501, 0,8319]	0,74	0,73	0,14	0,02
**Fatigue POS**	**oversample**	**0,82**	**0,06**	**[0,7574, 0,8869]**	**0,82**	**0,06**	**[0,7882, 0,9333]**	**0,82**	**0,80**	**0,04**	**0,00**
	classweight	0,54	0,06	[0,4265, 0,6618]	0,54	0,07	[0,3910, 0,6745]	0,60	0,51	0,05	0,00
	no_balancing	0,63	0,06	[0,5147, 0,7500]	0,60	0,07	[0,4600, 0,7511]	0,60	0,48	0,02	0,00
**Lower-Limb strength NEG**	**oversample**	**0,71**	**0,06**	**[0,5556, 0,7778]**	**0,82**	**0,07**	**[0,6879, 0,8686]**	**0,83**	**0,77**	**0,11**	**0,01**
	classweight	0,69	0,06	[0,5971, 0,8324]	0,70	0,07	[0,6526, 0,7325]	0,71	0,75	0,11	0,01
	no_balancing	0,67	0,06	[0,5824, 0,7176]	0,71	0,07	[0,6749, 0,7439]	0,72	0,73	0,12	0,01
**Lower-Limb strength POS**	**oversample**	**0,71**	**0,06**	**[0,5833, 0,8056]**	**0,87**	**0,05**	**[0,7474, 0,9259]**	**0,87**	**0,75**	**0,04**	**0,00**
	classweight	0,69	0,06	[0,6118, 0,7474]	0,78	0,07	[0,6515, 0,8198]	0,68	0,66	0,08	0,01
	no_balancing	0,70	0,06	[0,5824, 0,8176]	0,80	0,07	[0,6589, 0,8478]	0,69	0,69	0,07	0,01
**Grip strength NEG**	**oversample**	**0,96**	**0,02**	**[0,9138, 0,9914]**	**0,99**	**0,01**	**[0,9572, 1,0000]**	**0,99**	**0,98**	**0,02**	**0,00**
	classweight	0,85	0,04	[0,7647, 0,9265]	0,81	0,10	[0,7010, 0,8062]	0,80	0,71	0,09	0,01
	no_balancing	0,85	0,04	[0,7647, 0,9265]	0,88	0,10	[0,8891, 0,9810]	0,83	0,70	0,11	0,01
**Grip strength POS**	**oversample**	**0,95**	**0,02**	**[0,9052, 0,9828]**	**0,97**	**0,02**	**[0,9201, 1,0000]**	**0,98**	**0,99**	**0,02**	**0,00**
	classweight	0,85	0,04	[0,7647, 0,9265]	0,78	0,12	[0,7534, 0,8173]	0,72	0,78	0,15	0,02
	no_balancing	0,85	0,04	[0,7647, 0,9265]	0,74	0,09	[0,6712, 0,8125]	0,84	0,75	0,15	0,02
**LAM NEG**	**oversample**	**0,85**	**0,05**	**[0,7829, 0,9537]**	**0,92**	**0,05**	**[0,8560, 0,9475]**	**0,89**	**0,84**	**0,08**	**0,01**

	classweight	0,69	0,06	[0,6706, 0,7059]	0,70	0,07	[0,6810, 0,7411]	0,74	0,73	0,04	0,00
	no_balancing	0,74	0,06	[0,6265, 0,7618]	0,72	0,07	[0,6905, 0,7629]	0,72	0,71	0,06	0,00
**LAM POS**	**oversample**	**0,83**	**0,04**	**[0,8195, 0,8902]**	**0,87**	**0,05**	**[0,8457, 0,9369]**	**0,90**	**0,85**	**0,09**	**0,01**
	classweight	0,70	0,06	[0,4853, 0,7062]	0,69	0,08	[0,6468, 0,7464]	0,70	0,71	0,07	0,00
	no_balancing	0,70	0,06	[0,4853, 0,7062]	0,71	0,07	[0,6652, 0,7522]	0,70	0,78	0,04	0,00
**Mobility NEG**	**oversample**	**0,89**	**0,02**	**[0,9060, 0,9829]**	**0,94**	**0,00**	**[0,8972, 1,0000]**	**0,91**	**0,88**	**0,02**	**0,00**
	classweight	0,87	0,04	[0,7794, 0,9412]	0,83	0,13	[0,8486, 0,8718]	0,86	0,87	0,10	0,01
	no_balancing	0,85	0,04	[0,7647, 0,9265]	0,90	0,10	[0,7896, 0,9254]	0,88	0,87	0,05	0,00
**Mobility POS**	**oversample**	**0,93**	**0,01**	**[0,9573, 1,0000]**	**0,96**	**0,00**	**[0,8970, 1,0000]**	**0,93**	**0,93**	**0,02**	**0,00**
	classweight	0,84	0,04	[0,7500, 0,9265]	0,85	0,10	[0,7640, 0,8638]	0,81	0,88	0,16	0,03
	no_balancing	0,84	0,05	[0,7500, 0,9265]	0,87	0,09	[0,8021, 0,9462]	0,81	0,85	0,13	0,02
**Lower-limb Power NEG**	**oversample**	**0,81**	**0,05**	**[0,6190, 0,8095]**	**0,90**	**0,05**	**[0,8073, 0,988]**	**0,78**	**0,85**	**0,07**	**0,00**
	classweight	0,63	0,06	[0,5000, 0,7500]	0,71	0,06	[0,5781, 0,8126]	0,80	0,50	0,05	0,00
	no_balancing	0,69	0,06	[0,5882, 0,7941]	0,74	0,06	[0,6098, 0,8515]	0,80	0,51	0,07	0,01
**Lower-limb Power POS**	**oversample**	**0,79**	**0,05**	**[0,6786, 0,8690]**	**0,82**	**0,04**	**[0,7413, 0,9161]**	**0,87**	**0,82**	**0,06**	**0,00**
	classweight	0,60	0,06	[0,5824, 0,7176]	0,73	0,07	[0,6976, 0,7721]	0,65	0,64	0,04	0,00
	no_balancing	0,63	0,06	[0,6118, 0,6471]	0,73	0,07	[0,6864, 0,7669]	0,64	0,65	0,03	0,00

**Flexibility NEG**	**oversample**	**0,82**	**0,04**	**[0,7333, 0,9000]**	**0,95**	**0,03**	**[0,8935, 0,9911]**	**0,97**	**0,87**	**0,03**	**0,00**
	classweight	0,65	0,06	[0,5294, 0,7504]	0,62	0,07	[0,5780, 0,7583]	0,78	0,72	0,08	0,01
	no_balancing	0,68	0,06	[0,5735, 0,7794]	0,61	0,08	[0,5527, 0,7624]	0,71	0,72	0,09	0,01
**Flexibility POS**	**oversample**	**0,89**	**0,04**	**[0,8194, 0,9583]**	**0,93**	**0,03**	**[0,8634, 0,9865]**	**0,93**	**0,86**	**0,04**	**0,00**
	classweight	0,60	0,06	[0,5727, 0,7273]	0,78	0,08	[0,6314, 0,8325]	0,86	0,76	0,07	0,01
	no_balancing	0,64	0,06	[0,5091, 0,7636]	0,80	0,08	[0,7414, 0,8600]	0,75	0,76	0,08	0,01
**Gait speed NEG**	**oversample**	**0,92**	**0,04**	**[0,8194, 0,9583]**	**0,98**	**0,03**	**[0,9634, 0,9965]**	**0,93**	**0,90**	**0,04**	**0,00**
	classweight	0,60	0,06	[0,5727, 0,7273]	0,58	0,08	[0,4314, 0,7325]	0,69	0,59	0,07	0,01
	no_balancing	0,64	0,06	[0,5091, 0,7636]	0,60	0,08	[0,4414, 0,7600]	0,72	0,61	0,08	0,01
**Gait speed POS**	**oversample**	**0,90**	**0,04**	**[0,8333, 0,9500]**	**0,99**	**0,03**	**[0,9935, 0,9991]**	**0,97**	**0,92**	**0,03**	**0,00**
	classweight	0,65	0,06	[0,5294, 0,7504]	0,62	0,07	[0,4780, 0,7583]	0,79	0,62	0,08	0,01
	no_balancing	0,68	0,06	[0,5735, 0,7794]	0,61	0,08	[0,4527, 0,7624]	0,61	0,62	0,09	0,01

Performance comparison of base machine learning models for classifying physical deficits using negative (NEG) and positive (POS) emotional speech tasks. Metrics include accuracy (Acc), area under the ROC curve (AUC), average precision, and cross-validation performance with 95% confidence intervals. Three class balancing strategies were evaluated: oversampling (SMOTE), class weighting, and no balancing.

Supplementary Table 3 : Synthesis of combined models parameters

Comparative performance evaluation of stacking ensemble models with different meta-learners (Logistic Regression, Support Vector Machine, Random Forest, XGBoost) across ten physical function domains. Metrics include accuracy, cross-validated AUC (CV-AUC), test set AUC, precision-recall AUC (PR), Cohen's Kappa (CK), logarithmic loss (LL), and class-specific precision, recall, and F1-scores for deficit (D) and normal (N) classes. Random baseline performance provided for reference.

**Lower-limb endurance**	**Accuracy**	**CV-AUC**	**AUC**	**PR**	**CK**	**LL**	**Precision**		**Recall**		**F1-score**	
							**D**	**N**	**D**	**N**	**D**	**N**
**Random**	0.52	0.50	0.50	0.51	0.052	0.89	0.47	0.58	0.58	0.47	0.52	0.52
**LR**	0.95	0.90	0.95	0.94	0.89	0.26	0.93	0.97	0.97	0.92	0.95	0.95
**SVM**	0.96	0.91	0.91	0.95	0.91	0.18	0.91	0.99	0.99	0.91	0.96	0.95
**RF**	0.96	0.90	0.97	0.95	0.90	0.38	0.93	0.98	0.99	0.92	0.95	0.95
**XGB**	0.96	0.90	0.97	0.95	0.91	0.16	0.93	0.98	0.99	0.92	0.96	0.95
**Balance**	**Accuracy**	**CV-AUC**	**AUC**	**PR**	**CK**	**LL**	**Precision**		**Recall**		**F1-score**	
							**D**	**N**	**D**	**N**	**D**	**N**
**Random**	0.33	0.50	0.50	0.50	0.32	0.72	0.36	0.30	0.29	0.38	0.32	0.34
**LR**	0.93	0.92	0.95	0.92	0.89	0.28	0.84	0.83	0.83	0.84	0.83	0.83
**SVM**	0.94	0.90	0.94	0.93	0.88	0.32	0.84	0.83	0.83	0.84	0.83	0.83
**RF**	0.91	0.89	0.83	0.85	0.89	0.27	0.84	0.83	0.83	0.84	0.83	0.83
**XGB**	0.90	0.89	0.92	0.89	0.87	0.34	0.84	0.83	0.83	0.84	0.83	0.83
**Fatigue**	**Accuracy**	**CV-AUC**	**AUC**	**PR**	**CK**	**LL**	**Precision**		**Recall**		**F1-score**	
							**D**	**N**	**D**	**N**	**D**	**N**
**Random**	0.56	0.50	0.50	0.51	0.12	0.90	0.50	0.62	0.58	0.55	0.54	0.58
**LR**	0.71	0.78	0.81	0.87	0.72	0.35	0.73	0.69	0.70	0.73	0.72	0.71
**SVM**	0.77	0.76	0.79	0.86	0.75	0.28	0.91	0.70	0.63	0.73	0.74	0.80
**RF**	0.67	0.74	0.75	0.81	0.73	0.38	0.68	0.65	0.66	0.67	0.67	0.66
**XGB**	0.71	0.75	0.77	0.84	0.61	0.26	0.73	0.69	0.68	0.73	0.70	0.71
**Lower-limb strength**	**Accuracy**	**CV-AUC**	**AUC**	**PR**	**CK**	**LL**	**Precision**		**Recall**		**F1-score**	
							**D**	**N**	**D**	**N**	**D**	**N**
**Random**	0.47	0.50	0.50	0.49	0.05	0.98	0.49	0.46	0.46	0.49	0.47	0.47
**LR**	0.70	0.75	0.74	0.68	0.67	0.37	0.75	0.72	0.66	0.81	0.75	0.70
**SVM**	0.70	0.71	0.76	0.68	0.51	0.41	0.96	0.61	0.85	0.98	0.72	0.75
**RF**	0.72	0.73	0.75	0.75	0.60	0.45	0.91	0.73	0.73	0.94	0.77	0.76
**XGB**	0.71	0.73	0.74	0.64	0.56	0.43	0.88	0.63	0.53	0.92	0.66	0.75
**Grip strenght**	**Accuracy**	**CV-AUC**	**AUC**	**PR**	**CK**	**LL**	**Precision**		**Recall**		**F1-score**	
							**D**	**N**	**D**	**N**	**D**	**N**
**Random**	0.40	0.50	0.50	0.49	0.19	0.92	0.44	0.37	0.33	0.48	0.38	0.42
**LR**	0.77	0.76	0.79	0.86	0.65	0.38	0.81	0.70	0.63	0.73	0.74	0.80
**SVM**	0.90	0.95	0.94	0.96	0.91	0.16	0.98	0.97	0.89	0.95	0.92	0.94
**RF**	0.91	0.89	0.83	0.85	0.89	0.27	0.84	0.83	0.83	0.84	0.83	0.83
**XGB**	0.90	0.89	0.92	0.89	0.87	0.34	0.84	0.83	0.83	0.84	0.83	0.83
**LAM**	**Accuracy**	**CV-AUC**	**AUC**	**PR**	**CK**	**LL**	**Precision**		**Recall**		**F1-score**	
							**D**	**N**	**D**	**N**	**D**	**N**

**Random**	0.58	0.50	0.50	0.50	0.26	0.72	0.63	0.54	0.57	0.60	0.60	0.57
**LR**	0.81	0.82	0.88	0.89	0.63	0.42	0.91	0.73	0.72	0.92	0.81	0.81
**SVM**	0.80	0.80	0.84	0.86	0.69	0.37	0.98	0.74	0.71	0.98	0.83	0.85
**RF**	0.81	0.82	0.83	0.80	0.61	0.47	0.89	0.73	0.72	0.89	0.80	0.80
**XGB**	0.81	0.81	0.82	0.85	0.62	0.42	0.90	0.74	0.74	0.89	0.81	0.81
**Mobility**	**Accuracy**	**CV-AUC**	**AUC**	**PR**	**CK**	**LL**	**Precision**		**Recall**		**F1-score**	
							**D**	**N**	**D**	**N**	**D**	**N**
**Random**	0.52	0.50	0.50	0.51	0.06	0.96	0.54	0.51	0.53	0.53	0.54	0.52
**LR**	0.59	0.57	0.60	0.62	0.52	0.48	0.55	0.77	0.73	0.62	0.73	0.72
**SVM**	0.62	0.65	0.62	0.63	0.57	0.48	0.55	0.77	0.73	0.62	0.73	0.72
**RF**	0.92	0.95	0.97	0.96	0.82	0.22	0.95	0.89	0.87	0.97	0.86	0.88
**XGB**	0.94	0.98	0.98	0.98	0.87	0.20	0.89	0.99	0.99	0.87	0.94	0.93
**Lower-limb power**	**Accuracy**	**CV-AUC**	**AUC**	**PR**	**CK**	**LL**	**Precision**		**Recall**		**F1-score**	
							**D**	**N**	**D**	**N**	**D**	**N**
**Random**	0.46	0.50	0.50	0.49	0.07	0.98	0.45	0.48	0.44	0.49	0.44	0.48
**LR**	0.87	0.85	0.89	0.90	0.73	0.39	0.96	0.81	0.76	0.97	0.84	0.88
**SVM**	0.85	0.90	0.83	0.90	0.70	0.34	0.98	0.77	0.72	0.99	0.83	0.87
**RF**	0.85	0.81	0.87	0.83	0.70	0.31	0.96	0.79	0.72	097	0.83	0.87
**XGB**	0.85	0.81	0.88	0.89	0.70	0.37	0.96	0.79	0.73	0.97	0.83	0.87
**Flexibility**	**Accuracy**	**CV-AUC**	**AUC**	**PR**	**CK**	**LL**	**Precision**		**Recall**		**F1-score**	
							**D**	**N**	**D**	**N**	**D**	**N**
**Random**	0.48	0.50	0.50	0.49	0.06	0.94	0.33	0.60	0.43	0.50	0.38	0.55
**LR**	0.90	0.93	0.95	0.97	0.87	0.16	0.98	0.87	0.89	0.95	0.92	0.94
**SVM**	0.89	0.90	0.93	0.92	0.77	0.31	0.96	0.79	0.72	097	0.83	0.87
**RF**	0.87	0.89	0.92	0.90	0.63	0.48	0.85	0.77	0.73	0.62	0.73	0.72
**XGB**	0.88	0.90	0.92	0.93	0.79	0.27	0.84	0.83	0.83	0.84	0.83	0.83
**Walk speed**	**Accuracy**	**CV-AUC**	**AUC**	**PR**	**CK**	**LL**	**Precision**		**Recall**		**F1-score**	
							**D**	**N**	**D**	**N**	**D**	**N**
**Random**	0.52	0.50	0.50	0.50	0.04	0.96	0.51	0.53	0.50	0.54	0.51	0.54
**LR**	0.88	0.89	0.94	0.89	0.78	0.32	0.89	0.89	0.89	0.89	0.89	0.89
**SVM**	0.86	0.92	0.92	0.87	0.84	0.24	0.98	0.88	0.86	0.98	0.92	0.93
**RF**	0.89	0.86	0.93	0.90	0.79	0.51	0.91	0.88	0.88	0.91	0.89	0.90
**XGB**	0.87	0.93	0.93	0.92	0.86	0.27	0.98	0.88	0.87	0.98	0.93	0.93

task: Physical task assessed; Accuracy: Overall accuracy; CV-AUC: Area under the ROC curve via cross-validation; AUC: Overall area under the ROC curve; PR: Area under the Precision-Recall curve; CK: Cohen's Kappa coefficient; LL: Log Loss (Logarithmic loss); Precision: Precision; Recall: Recall; F1-score: F1-score (harmonic mean of precision and recall); D: Metrics for Deficient class; N: Metrics for Normal class; POS: Positive speech condition; NEG: Negative speech condition; LR: Logistic Regression; SVM: Support Vector Machines; RF: Random Forests; XGB: Extreme Gradient Boosting; Balance: Postural balance task

Supplementary Table 4 : Bayesian comparisons of classification accuracies across Machine Learning models

Bayesian pairwise comparisons of classification performance between machine learning models across physical function domains. Results present posterior probabilities for model superiority, practical equivalence, or inferiority based on Region of Practical Equivalence (ROPE) analysis. Values indicate the probability that the row model outperforms the column model, with ROPE-defined equivalence zones accounting for negligible performance differences.

Lower-Limb Endurance

Condition	Model_1	Model_2	P(Model_1 > Model_2)	P(Equal)	P(Model_2 > Model_1)
**POS**	Random	LR	0,00	0,00	1,00
**POS**	Random	SVM	0,00	0,00	1,00
**POS**	Random	RF	0,00	0,00	1,00
**POS**	Random	XGB	0,00	0,00	1,00
**POS**	LR	SVM	0,00	0,00	1,00
**POS**	LR	RF	0,00	0,00	1,00
**POS**	LR	XGB	0,00	0,00	1,00
**POS**	SVM	RF	0,06	0,07	0,87
**POS**	SVM	XGB	0,09	0,11	0,80
**POS**	RF	XGB	0,50	0,46	0,04
**NEG**	Random	LR	0,00	0,00	1,00
**NEG**	Random	SVM	0,00	0,00	1,00
**NEG**	Random	RF	0,00	0,00	1,00
**NEG**	Random	XGB	0,00	0,00	1,00
**NEG**	LR	SVM	0,00	0,00	1,00
**NEG**	LR	RF	0,00	0,00	1,00
**NEG**	LR	XGB	0,00	0,00	1,00
**NEG**	SVM	RF	0,08	0,13	0,79
**NEG**	SVM	XGB	0,21	0,11	0,68
**NEG**	RF	XGB	0,37	0,19	0,44
**Combined**	Random	LR	0,00	0,00	1,00
**Combined**	Random	SVM	0,00	0,00	1,00
**Combined**	Random	RF	0,00	0,00	1,00
**Combined**	Random	XGB	0,00	0,00	1,00
**Combined**	LR	SVM	0,86	0,09	0,05
**Combined**	LR	RF	0,96	0,02	0,01
**Combined**	LR	XGB	0,90	0,06	0,04
**Combined**	SVM	RF	0,59	0,30	0,11
**Combined**	SVM	XGB	0,50	0,28	0,22
**Combined**	RF	XGB	0,16	0,52	0,32
**POS_vs_NEG**	LR (POS)	LR (NEG)	0,01	0,02	0,97
**POS_vs_NEG**	SVM (POS)	SVM (NEG)	0,39	0,15	0,45

**POS_vs_NEG**	XGB (POS)	XGB (NEG)	0,36	0,23	0,41
**POS_vs_NEG**	RF (POS)	RF (NEG)	0,54	0,31	0,14
**POS_vs_NEG**	Random (POS)	Random (NEG)	0,00	1,00	0,00
**Combined_vs_POS**	LR (Combined)	LR (POS)	1,00	0,00	0,00
**Combined_vs_POS**	SVM (Combined)	SVM (POS)	0,87	0,07	0,07
**Combined_vs_POS**	XGB (Combined)	XGB (POS)	0,44	0,30	0,27
**Combined_vs_POS**	RF (Combined)	RF (POS)	0,30	0,23	0,48
**Combined_vs_POS**	Random (Combined)	Random (POS)	0,00	1,00	0,00
**Combined_vs_NEG**	LR (Combined)	LR (NEG)	1,00	0,00	0,00
**Combined_vs_NEG**	SVM (Combined)	SVM (NEG)	0,95	0,03	0,02
**Combined_vs_NEG**	XGB (Combined)	XGB (NEG)	0,44	0,19	0,37
**Combined_vs_NEG**	RF (Combined)	RF (NEG)	0,38	0,38	0,24
**Combined_vs_NEG**	Random (Combined)	Random (NEG)	0,00	1,00	0,00
**POS_vs_NEG_top_models**	RF (POS)	XGB (NEG)	0,47	0,24	0,29
**POS_vs_NEG_top_models**	RF (POS)	RF (NEG)	0,54	0,31	0,14
**POS_vs_NEG_top_models**	XGB (POS)	XGB (NEG)	0,36	0,23	0,41
**POS_vs_NEG_top_models**	XGB (POS)	RF (NEG)	0,25	0,58	0,17
**POS_vs_Combined_top_models**	RF (POS)	LR (Combined)	0,04	0,07	0,89
**POS_vs_Combined_top_models**	RF (POS)	SVM (Combined)	0,31	0,23	0,45
**POS_vs_Combined_top_models**	XGB (POS)	LR (Combined)	0,01	0,02	0,96
**POS_vs_Combined_top_models**	XGB (POS)	SVM (Combined)	0,17	0,24	0,59
**NEG_vs_Combined_top_models**	XGB (NEG)	LR (Combined)	0,07	0,07	0,86
**NEG_vs_Combined_top_models**	XGB (NEG)	SVM (Combined)	0,30	0,17	0,54
**NEG_vs_Combined_top_models**	RF (NEG)	LR (Combined)	0,00	0,01	0,99
**NEG_vs_Combined_top_models**	RF (NEG)	SVM (Combined)	0,11	0,24	0,65

Lower-limb strength

**Condition**	**Model_1**	**Model_2**	**P(Model_1 > Model_2)**	**P(Equal)**	**P(Model_2 > Model_1)**
**POS**	Random	LR	0,03	0,03	0,94
**POS**	Random	SVM	0,00	0,00	1,00
**POS**	Random	RF	0,00	0,00	1,00
**POS**	Random	XGB	0,00	0,00	1,00
**POS**	LR	SVM	0,03	0,02	0,95
**POS**	LR	RF	0,00	0,00	1,00

**POS**	LR	XGB	0,01	0,00	0,99
**POS**	SVM	RF	0,04	0,04	0,93
**POS**	SVM	XGB	0,01	0,01	0,98
**POS**	RF	XGB	0,47	0,28	0,25
**NEG**	Random	LR	0,00	0,00	1,00
**NEG**	Random	SVM	0,00	0,00	1,00
**NEG**	Random	RF	0,00	0,00	1,00
**NEG**	Random	XGB	0,00	0,00	1,00
**NEG**	LR	SVM	0,07	0,04	0,89
**NEG**	LR	RF	0,00	0,00	0,99
**NEG**	LR	XGB	0,00	0,00	1,00
**NEG**	SVM	RF	0,01	0,01	0,98
**NEG**	SVM	XGB	0,09	0,05	0,86
**NEG**	RF	XGB	0,47	0,25	0,28
**Combined**	Random	LR	0,00	0,00	1,00
**Combined**	Random	SVM	0,00	0,00	1,00
**Combined**	Random	RF	0,00	0,00	1,00
**Combined**	Random	XGB	0,00	0,00	1,00
**Combined**	LR	SVM	0,87	0,08	0,05
**Combined**	LR	RF	0,71	0,17	0,13
**Combined**	LR	XGB	0,64	0,16	0,21
**Combined**	SVM	RF	0,14	0,25	0,61
**Combined**	SVM	XGB	0,14	0,23	0,63
**Combined**	RF	XGB	0,22	0,49	0,30
**POS_vs_NEG**	XGB (POS)	XGB (NEG)	0,12	0,21	0,66
**POS_vs_NEG**	LR (POS)	LR (NEG)	0,19	0,11	0,69
**POS_vs_NEG**	Random (POS)	Random (NEG)	0,00	1,00	0,00
**POS_vs_NEG**	RF (POS)	RF (NEG)	0,04	0,20	0,75
**POS_vs_NEG**	SVM (POS)	SVM (NEG)	0,35	0,15	0,50
**Combined_vs_POS**	XGB (Combined)	XGB (POS)	0,19	0,16	0,65
**Combined_vs_POS**	LR (Combined)	LR (POS)	0,99	0,00	0,01
**Combined_vs_POS**	Random (Combined)	Random (POS)	0,00	1,00	0,00
**Combined_vs_POS**	RF (Combined)	RF (POS)	0,02	0,07	0,90
**Combined_vs_POS**	SVM (Combined)	SVM (POS)	0,60	0,20	0,19
**Combined_vs_NEG**	XGB (Combined)	XGB (NEG)	0,05	0,07	0,88
**Combined_vs_NEG**	LR (Combined)	LR (NEG)	0,99	0,00	0,00
**Combined_vs_NEG**	Random (Combined)	Random (NEG)	0,00	1,00	0,00
**Combined_vs_NEG**	RF (Combined)	RF (NEG)	0,01	0,02	0,96
**Combined_vs_NEG**	SVM (Combined)	SVM (NEG)	0,48	0,16	0,36
**POS_vs_NEG_top_models**	RF (POS)	RF (NEG)	0,04	0,20	0,75
**POS_vs_NEG_top_models**	RF (POS)	XGB (NEG)	0,19	0,28	0,53

**POS_vs_NEG_top_models**	XGB (POS)	RF (NEG)	0,08	0,15	0,77
**POS_vs_NEG_top_models**	XGB (POS)	XGB (NEG)	0,12	0,21	0,66
**POS_vs_Combined_top_models**	RF (POS)	LR (Combined)	0,47	0,25	0,27
**POS_vs_Combined_top_models**	RF (POS)	XGB (Combined)	0,77	0,13	0,10
**POS_vs_Combined_top_models**	XGB (POS)	LR (Combined)	0,35	0,29	0,35
**POS_vs_Combined_top_models**	XGB (POS)	XGB (Combined)	0,65	0,16	0,19
**NEG_vs_Combined_top_models**	RF (NEG)	LR (Combined)	0,79	0,14	0,06
**NEG_vs_Combined_top_models**	RF (NEG)	XGB (Combined)	0,87	0,07	0,06
**NEG_vs_Combined_top_models**	XGB (NEG)	LR (Combined)	0,63	0,20	0,17
**NEG_vs_Combined_top_models**	XGB (NEG)	XGB (Combined)	0,88	0,07	0,05

Lower-limb power

Condition	Model_1	Model_2	P(Model_1 > Model_2)	P(Equal)	P(Model_2 > Model_1)
**POS**	Random	LR	0,00	0,00	1,00
**POS**	Random	SVM	0,00	0,00	1,00
**POS**	Random	RF	0,00	0,00	1,00
**POS**	Random	XGB	0,00	0,00	1,00
**POS**	LR	SVM	0,02	0,04	0,95
**POS**	LR	RF	0,89	0,07	0,04
**POS**	LR	XGB	0,90	0,06	0,04
**POS**	SVM	RF	1,00	0,00	0,00
**POS**	SVM	XGB	1,00	0,00	0,00
**POS**	RF	XGB	0,31	0,45	0,24
**NEG**	Random	LR	0,00	0,00	1,00
**NEG**	Random	SVM	0,00	0,00	1,00
**NEG**	Random	RF	0,00	0,00	1,00
**NEG**	Random	XGB	0,00	0,00	1,00
**NEG**	LR	SVM	0,53	0,24	0,23
**NEG**	LR	RF	0,93	0,05	0,01
**NEG**	LR	XGB	0,88	0,11	0,01
**NEG**	SVM	RF	0,64	0,16	0,20
**NEG**	SVM	XGB	0,50	0,21	0,29
**NEG**	RF	XGB	0,12	0,29	0,58
**Combined**	Random	LR	0,10	0,01	0,88
**Combined**	Random	SVM	0,04	0,01	0,95
**Combined**	Random	RF	0,05	0,01	0,94
**Combined**	Random	XGB	0,04	0,01	0,96
**Combined**	LR	SVM	0,30	0,09	0,60
**Combined**	LR	RF	0,09	0,05	0,86

**Combined**	LR	XGB	0,10	0,04	0,87
**Combined**	SVM	RF	0,10	0,09	0,81
**Combined**	SVM	XGB	0,06	0,05	0,89
**Combined**	RF	XGB	0,13	0,18	0,69
**POS_vs_NEG**	SVM (POS)	SVM (NEG)	0,81	0,06	0,12
**POS_vs_NEG**	Random (POS)	Random (NEG)	0,00	1,00	0,00
**POS_vs_NEG**	LR (POS)	LR (NEG)	0,47	0,15	0,38
**POS_vs_NEG**	XGB (POS)	XGB (NEG)	0,30	0,15	0,55
**POS_vs_NEG**	RF (POS)	RF (NEG)	0,42	0,17	0,42
**Combined_vs_POS**	SVM (Combined)	SVM (POS)	0,01	0,00	0,98
**Combined_vs_POS**	Random (Combined)	Random (POS)	0,22	0,04	0,73
**Combined_vs_POS**	LR (Combined)	LR (POS)	0,10	0,02	0,88
**Combined_vs_POS**	XGB (Combined)	XGB (POS)	0,36	0,08	0,55
**Combined_vs_POS**	RF (Combined)	RF (POS)	0,26	0,07	0,67
**Combined_vs_NEG**	SVM (Combined)	SVM (NEG)	0,08	0,03	0,89
**Combined_vs_NEG**	Random (Combined)	Random (NEG)	0,22	0,04	0,73
**Combined_vs_NEG**	LR (Combined)	LR (NEG)	0,15	0,03	0,83
**Combined_vs_NEG**	XGB (Combined)	XGB (NEG)	0,33	0,07	0,60
**Combined_vs_NEG**	RF (Combined)	RF (NEG)	0,31	0,06	0,63
**POS_vs_NEG_top_models**	SVM (POS)	LR (NEG)	0,80	0,08	0,12
**POS_vs_NEG_top_models**	SVM (POS)	SVM (NEG)	0,81	0,06	0,12
**POS_vs_NEG_top_models**	LR (POS)	LR (NEG)	0,47	0,15	0,38
**POS_vs_NEG_top_models**	LR (POS)	SVM (NEG)	0,56	0,14	0,30
**POS_vs_Combined_top_models**	SVM (POS)	XGB (Combined)	0,86	0,03	0,11
**POS_vs_Combined_top_models**	SVM (POS)	RF (Combined)	0,90	0,02	0,07
**POS_vs_Combined_top_models**	LR (POS)	XGB (Combined)	0,72	0,06	0,22
**POS_vs_Combined_top_models**	LR (POS)	RF (Combined)	0,81	0,05	0,15
**NEG_vs_Combined_top_models**	LR (NEG)	XGB (Combined)	0,67	0,06	0,27
**NEG_vs_Combined_top_models**	LR (NEG)	RF (Combined)	0,74	0,05	0,22
**NEG_vs_Combined_top_models**	SVM (NEG)	XGB (Combined)	0,62	0,06	0,32
**NEG_vs_Combined_top_models**	SVM (NEG)	RF (Combined)	0,70	0,05	0,25

Grip strength

**Condition**	**Model_1**	**Model_2**	**P(Model_1 > Model_2)**	**P(Equal)**	**P(Model_2 > Model_1)**

**POS**	Random	LR	0,00	0,00	1,00
**POS**	Random	SVM	0,00	0,00	1,00
**POS**	Random	RF	0,00	0,00	1,00
**POS**	Random	XGB	0,00	0,00	1,00
**POS**	LR	SVM	0,00	0,00	1,00
**POS**	LR	RF	0,00	0,00	1,00
**POS**	LR	XGB	0,00	0,00	1,00
**POS**	SVM	RF	0,01	0,02	0,97
**POS**	SVM	XGB	0,03	0,06	0,90
**POS**	RF	XGB	0,98	0,02	0,00
**NEG**	Random	LR	0,00	0,00	1,00
**NEG**	Random	SVM	0,00	0,00	1,00
**NEG**	Random	RF	0,00	0,00	1,00
**NEG**	Random	XGB	0,00	0,00	1,00
**NEG**	LR	SVM	0,01	0,00	0,99
**NEG**	LR	RF	0,00	0,00	1,00
**NEG**	LR	XGB	0,00	0,00	1,00
**NEG**	SVM	RF	0,16	0,13	0,71
**NEG**	SVM	XGB	0,15	0,21	0,64
**NEG**	RF	XGB	0,52	0,20	0,28
**Combined**	Random	LR	0,00	0,00	1,00
**Combined**	Random	SVM	0,00	0,00	1,00
**Combined**	Random	RF	0,00	0,00	1,00
**Combined**	Random	XGB	0,00	0,00	1,00
**Combined**	LR	SVM	0,00	0,00	1,00
**Combined**	LR	RF	0,02	0,01	0,97
**Combined**	LR	XGB	0,02	0,01	0,97
**Combined**	SVM	RF	0,99	0,01	0,00
**Combined**	SVM	XGB	0,99	0,00	0,00
**Combined**	RF	XGB	0,24	0,53	0,24
**POS_vs_NEG**	Random (POS)	Random (NEG)	0,00	1,00	0,00
**POS_vs_NEG**	SVM (POS)	SVM (NEG)	0,36	0,35	0,29
**POS_vs_NEG**	LR (POS)	LR (NEG)	0,65	0,12	0,23
**POS_vs_NEG**	RF (POS)	RF (NEG)	0,83	0,09	0,08
**POS_vs_NEG**	XGB (POS)	XGB (NEG)	0,69	0,18	0,13
**Combined_vs_POS**	Random (Combined)	Random (POS)	0,00	1,00	0,00
**Combined_vs_POS**	SVM (Combined)	SVM (POS)	0,85	0,08	0,07
**Combined_vs_POS**	LR (Combined)	LR (POS)	0,59	0,13	0,28
**Combined_vs_POS**	RF (Combined)	RF (POS)	0,00	0,00	0,99
**Combined_vs_POS**	XGB (Combined)	XGB (POS)	0,02	0,04	0,94
**Combined_vs_NEG**	Random (Combined)	Random (NEG)	0,00	1,00	0,00
**Combined_vs_NEG**	SVM (Combined)	SVM (NEG)	0,98	0,01	0,00
**Combined_vs_NEG**	LR (Combined)	LR (NEG)	0,70	0,08	0,23

**Combined_vs_NEG**	RF (Combined)	RF (NEG)	0,14	0,11	0,74
**Combined_vs_NEG**	XGB (Combined)	XGB (NEG)	0,12	0,17	0,72
**POS_vs_NEG_top_models**	RF (POS)	RF (NEG)	0,83	0,09	0,08
**POS_vs_NEG_top_models**	RF (POS)	XGB (NEG)	0,94	0,04	0,02
**POS_vs_NEG_top_models**	XGB (POS)	RF (NEG)	0,52	0,21	0,27
**POS_vs_NEG_top_models**	XGB (POS)	XGB (NEG)	0,69	0,18	0,13
**POS_vs_Combined_top_models**	RF (POS)	SVM (Combined)	0,71	0,18	0,11
**POS_vs_Combined_top_models**	RF (POS)	RF (Combined)	0,99	0,00	0,00
**POS_vs_Combined_top_models**	XGB (POS)	SVM (Combined)	0,28	0,28	0,44
**POS_vs_Combined_top_models**	XGB (POS)	RF (Combined)	0,97	0,02	0,01
**NEG_vs_Combined_top_models**	RF (NEG)	SVM (Combined)	0,26	0,17	0,58
**NEG_vs_Combined_top_models**	RF (NEG)	RF (Combined)	0,74	0,11	0,14
**NEG_vs_Combined_top_models**	XGB (NEG)	SVM (Combined)	0,14	0,14	0,71
**NEG_vs_Combined_top_models**	XGB (NEG)	RF (Combined)	0,69	0,16	0,14

Walk speed

**Condition**	**Model_1**	**Model_2**	**P(Model_1 > Model_2)**	**P(Equal)**	**P(Model_2 > Model_1)**
**POS**	Random	LR	0,00	0,00	0,99
**POS**	Random	SVM	0,00	0,00	1,00
**POS**	Random	RF	0,00	0,00	1,00
**POS**	Random	XGB	0,00	0,00	1,00
**POS**	LR	SVM	0,20	0,05	0,75
**POS**	LR	RF	0,02	0,01	0,97
**POS**	LR	XGB	0,01	0,01	0,98
**POS**	SVM	RF	0,09	0,05	0,86
**POS**	SVM	XGB	0,18	0,07	0,75
**POS**	RF	XGB	0,58	0,29	0,13
**NEG**	Random	LR	0,01	0,00	0,99
**NEG**	Random	SVM	0,00	0,00	1,00
**NEG**	Random	RF	0,00	0,00	1,00
**NEG**	Random	XGB	0,00	0,00	1,00
**NEG**	LR	SVM	0,23	0,06	0,71
**NEG**	LR	RF	0,01	0,00	0,99
**NEG**	LR	XGB	0,02	0,01	0,97
**NEG**	SVM	RF	0,03	0,01	0,96
**NEG**	SVM	XGB	0,02	0,01	0,96
**NEG**	RF	XGB	0,72	0,22	0,06

**Combined**	Random	LR	0,00	0,00	1,00
**Combined**	Random	SVM	0,00	0,00	1,00
**Combined**	Random	RF	0,00	0,00	1,00
**Combined**	Random	XGB	0,00	0,00	1,00
**Combined**	LR	SVM	0,03	0,03	0,94
**Combined**	LR	RF	0,00	0,00	1,00
**Combined**	LR	XGB	0,00	0,00	0,99
**Combined**	SVM	RF	0,00	0,00	1,00
**Combined**	SVM	XGB	0,00	0,00	0,99
**Combined**	RF	XGB	0,63	0,27	0,10
**POS_vs_NEG**	SVM (POS)	SVM (NEG)	0,72	0,05	0,22
**POS_vs_NEG**	LR (POS)	LR (NEG)	0,67	0,07	0,27
**POS_vs_NEG**	Random (POS)	Random (NEG)	0,00	1,00	0,00
**POS_vs_NEG**	RF (POS)	RF (NEG)	0,63	0,36	0,01
**POS_vs_NEG**	XGB (POS)	XGB (NEG)	0,69	0,24	0,07
**Combined_vs_POS**	SVM (Combined)	SVM (POS)	0,20	0,11	0,69
**Combined_vs_POS**	LR (Combined)	LR (POS)	0,24	0,13	0,63
**Combined_vs_POS**	Random (Combined)	Random (POS)	0,00	1,00	0,00
**Combined_vs_POS**	RF (Combined)	RF (POS)	0,01	0,83	0,17
**Combined_vs_POS**	XGB (Combined)	XGB (POS)	0,05	0,53	0,42
**Combined_vs_NEG**	SVM (Combined)	SVM (NEG)	0,70	0,11	0,20
**Combined_vs_NEG**	LR (Combined)	LR (NEG)	0,63	0,14	0,23
**Combined_vs_NEG**	Random (Combined)	Random (NEG)	0,00	1,00	0,00
**Combined_vs_NEG**	RF (Combined)	RF (NEG)	0,17	0,83	0,01
**Combined_vs_NEG**	XGB (Combined)	XGB (NEG)	0,50	0,48	0,02
**POS_vs_NEG_top_models**	RF (POS)	RF (NEG)	0,63	0,36	0,01
**POS_vs_NEG_top_models**	RF (POS)	XGB (NEG)	0,84	0,11	0,05
**POS_vs_NEG_top_models**	XGB (POS)	RF (NEG)	0,26	0,42	0,33
**POS_vs_NEG_top_models**	XGB (POS)	XGB (NEG)	0,69	0,24	0,07
**POS_vs_Combined_top_models**	RF (POS)	RF (Combined)	0,17	0,83	0,01
**POS_vs_Combined_top_models**	RF (POS)	XGB (Combined)	0,74	0,19	0,07
**POS_vs_Combined_top_models**	XGB (POS)	RF (Combined)	0,19	0,35	0,46
**POS_vs_Combined_top_models**	XGB (POS)	XGB (Combined)	0,42	0,53	0,05
**NEG_vs_Combined_top_models**	RF (NEG)	RF (Combined)	0,01	0,83	0,17
**NEG_vs_Combined_top_models**	RF (NEG)	XGB (Combined)	0,50	0,38	0,12
**NEG_vs_Combined_top_models**	XGB (NEG)	RF (Combined)	0,06	0,15	0,79

**NEG_vs_Combined_top_models**	XGB (NEG)	XGB (Combined)	0,02	0,48	0,50

Mobility

Condition	Model_1	Model_2	P(Model_1 > Model_2)	P(Equal)	P(Model_2 > Model_1)
**POS**	Random	LR	0,00	0,00	1,00
**POS**	Random	SVM	0,00	0,00	1,00
**POS**	Random	RF	0,00	0,00	1,00
**POS**	Random	XGB	0,00	0,00	1,00
**POS**	LR	SVM	0,02	0,02	0,95
**POS**	LR	RF	0,04	0,05	0,91
**POS**	LR	XGB	0,04	0,02	0,94
**POS**	SVM	RF	0,77	0,18	0,05
**POS**	SVM	XGB	0,19	0,14	0,67
**POS**	RF	XGB	0,11	0,08	0,81
**NEG**	Random	LR	0,00	0,00	1,00
**NEG**	Random	SVM	0,00	0,00	1,00
**NEG**	Random	RF	0,00	0,00	1,00
**NEG**	Random	XGB	0,00	0,00	1,00
**NEG**	LR	SVM	0,04	0,08	0,89
**NEG**	LR	RF	0,01	0,04	0,94
**NEG**	LR	XGB	0,03	0,02	0,95
**NEG**	SVM	RF	0,12	0,67	0,21
**NEG**	SVM	XGB	0,12	0,07	0,81
**NEG**	RF	XGB	0,10	0,07	0,83
**Combined**	Random	LR	0,00	0,00	0,99
**Combined**	Random	SVM	0,00	0,00	1,00
**Combined**	Random	RF	0,00	0,00	1,00
**Combined**	Random	XGB	0,00	0,00	1,00
**Combined**	LR	SVM	0,04	0,03	0,94
**Combined**	LR	RF	0,00	0,00	1,00
**Combined**	LR	XGB	0,00	0,00	1,00
**Combined**	SVM	RF	0,00	0,00	1,00
**Combined**	SVM	XGB	0,00	0,00	1,00
**Combined**	RF	XGB	0,01	0,08	0,91
**POS_vs_NEG**	SVM (POS)	SVM (NEG)	0,85	0,11	0,04
**POS_vs_NEG**	LR (POS)	LR (NEG)	0,10	0,55	0,36
**POS_vs_NEG**	RF (POS)	RF (NEG)	0,46	0,35	0,19
**POS_vs_NEG**	Random (POS)	Random (NEG)	0,00	1,00	0,00
**POS_vs_NEG**	XGB (POS)	XGB (NEG)	0,12	0,67	0,21
**Combined_vs_POS**	SVM (Combined)	SVM (POS)	0,00	0,00	1,00
**Combined_vs_POS**	LR (Combined)	LR (POS)	0,00	0,00	1,00
**Combined_vs_POS**	RF (Combined)	RF (POS)	0,87	0,10	0,03

**Combined_vs_POS**	Random (Combined)	Random (POS)	0,00	1,00	0,00
**Combined_vs_POS**	XGB (Combined)	XGB (POS)	0,41	0,28	0,31
**Combined_vs_NEG**	SVM (Combined)	SVM (NEG)	0,00	0,00	1,00
**Combined_vs_NEG**	LR (Combined)	LR (NEG)	0,00	0,00	1,00
**Combined_vs_NEG**	RF (Combined)	RF (NEG)	0,80	0,10	0,10
**Combined_vs_NEG**	Random (Combined)	Random (NEG)	0,00	1,00	0,00
**Combined_vs_NEG**	XGB (Combined)	XGB (NEG)	0,35	0,37	0,28
**POS_vs_NEG_top_models**	XGB (POS)	XGB (NEG)	0,12	0,67	0,21
**POS_vs_NEG_top_models**	XGB (POS)	RF (NEG)	0,81	0,07	0,12
**POS_vs_NEG_top_models**	SVM (POS)	XGB (NEG)	0,17	0,14	0,69
**POS_vs_NEG_top_models**	SVM (POS)	RF (NEG)	0,91	0,08	0,02
**POS_vs_Combined_top_models**	XGB (POS)	XGB (Combined)	0,31	0,28	0,41
**POS_vs_Combined_top_models**	XGB (POS)	RF (Combined)	0,61	0,21	0,18
**POS_vs_Combined_top_models**	SVM (POS)	XGB (Combined)	0,11	0,13	0,75
**POS_vs_Combined_top_models**	SVM (POS)	RF (Combined)	0,23	0,27	0,50
**NEG_vs_Combined_top_models**	XGB (NEG)	XGB (Combined)	0,28	0,37	0,35
**NEG_vs_Combined_top_models**	XGB (NEG)	RF (Combined)	0,66	0,21	0,13
**NEG_vs_Combined_top_models**	RF (NEG)	XGB (Combined)	0,06	0,05	0,89
**NEG_vs_Combined_top_models**	RF (NEG)	RF (Combined)	0,10	0,10	0,80

Lean Appendicular Mass

**Condition**	**Model_1**	**Model_2**	**P(Model_1 > Model_2)**	**P(Equal)**	**P(Model_2 > Model_1)**
**POS**	Random	LR	0,01	0,00	0,99
**POS**	Random	SVM	0,00	0,00	1,00
**POS**	Random	RF	0,00	0,00	1,00
**POS**	Random	XGB	0,00	0,00	1,00
**POS**	LR	SVM	0,40	0,07	0,52
**POS**	LR	RF	0,01	0,01	0,99
**POS**	LR	XGB	0,09	0,04	0,88
**POS**	SVM	RF	0,07	0,03	0,90
**POS**	SVM	XGB	0,03	0,02	0,94
**POS**	RF	XGB	0,66	0,11	0,22
**NEG**	Random	LR	0,02	0,01	0,97
**NEG**	Random	SVM	0,01	0,00	0,99
**NEG**	Random	RF	0,00	0,00	1,00
**NEG**	Random	XGB	0,00	0,00	1,00

**NEG**	LR	SVM	0,13	0,05	0,81
**NEG**	LR	RF	0,00	0,00	1,00
**NEG**	LR	XGB	0,00	0,00	1,00
**NEG**	SVM	RF	0,08	0,03	0,89
**NEG**	SVM	XGB	0,08	0,04	0,88
**NEG**	RF	XGB	0,71	0,17	0,12
**Combined**	Random	LR	0,00	0,00	1,00
**Combined**	Random	SVM	0,00	0,00	1,00
**Combined**	Random	RF	0,00	0,00	1,00
**Combined**	Random	XGB	0,00	0,00	1,00
**Combined**	LR	SVM	0,66	0,21	0,12
**Combined**	LR	RF	0,34	0,32	0,34
**Combined**	LR	XGB	0,56	0,24	0,20
**Combined**	SVM	RF	0,02	0,16	0,82
**Combined**	SVM	XGB	0,05	0,63	0,31
**Combined**	RF	XGB	0,59	0,30	0,11
**POS_vs_NEG**	XGB (POS)	XGB (NEG)	0,50	0,13	0,37
**POS_vs_NEG**	RF (POS)	RF (NEG)	0,63	0,23	0,14
**POS_vs_NEG**	SVM (POS)	SVM (NEG)	0,51	0,09	0,40
**POS_vs_NEG**	Random (POS)	Random (NEG)	0,00	1,00	0,00
**POS_vs_NEG**	LR (POS)	LR (NEG)	0,94	0,03	0,03
**Combined_vs_POS**	XGB (Combined)	XGB (POS)	0,34	0,15	0,52
**Combined_vs_POS**	RF (Combined)	RF (POS)	0,16	0,13	0,71
**Combined_vs_POS**	SVM (Combined)	SVM (POS)	0,85	0,06	0,09
**Combined_vs_POS**	Random (Combined)	Random (POS)	0,00	1,00	0,00
**Combined_vs_POS**	LR (Combined)	LR (POS)	0,91	0,03	0,06
**Combined_vs_NEG**	XGB (Combined)	XGB (NEG)	0,33	0,29	0,38
**Combined_vs_NEG**	RF (Combined)	RF (NEG)	0,28	0,18	0,54
**Combined_vs_NEG**	SVM (Combined)	SVM (NEG)	0,88	0,04	0,08
**Combined_vs_NEG**	Random (Combined)	Random (NEG)	0,00	1,00	0,00
**Combined_vs_NEG**	LR (Combined)	LR (NEG)	0,98	0,00	0,01
**POS_vs_NEG_top_models**	RF (POS)	RF (NEG)	0,63	0,23	0,14
**POS_vs_NEG_top_models**	RF (POS)	XGB (NEG)	0,98	0,02	0,01
**POS_vs_NEG_top_models**	XGB (POS)	RF (NEG)	0,33	0,13	0,54
**POS_vs_NEG_top_models**	XGB (POS)	XGB (NEG)	0,50	0,13	0,37
**POS_vs_Combined_top_models**	RF (POS)	LR (Combined)	0,77	0,13	0,10
**POS_vs_Combined_top_models**	RF (POS)	RF (Combined)	0,71	0,13	0,16
**POS_vs_Combined_top_models**	XGB (POS)	LR (Combined)	0,37	0,22	0,41

**POS_vs_Combined_top_ models**	XGB (POS)	RF (Combined)	0,37	0,22	0,41
**NEG_vs_Combined_top_ models**	RF (NEG)	LR (Combined)	0,54	0,18	0,28
**NEG_vs_Combined_top_ models**	RF (NEG)	RF (Combined)	0,54	0,18	0,28
**NEG_vs_Combined_top_ models**	XGB (NEG)	LR (Combined)	0,21	0,26	0,53
**NEG_vs_Combined_top_ models**	XGB (NEG)	RF (Combined)	0,28	0,20	0,52

Fatigue

Condition	Model_1	Model_2	P(Model_1 > Model_2)	P(Equ al)	P(Model_2 > Model_1)
**POS**	Random	LR	0,00	0,00	1,00
**POS**	Random	SVM	0,00	0,00	1,00
**POS**	Random	RF	0,00	0,00	1,00
**POS**	Random	XGB	0,00	0,00	1,00
**POS**	LR	SVM	0,00	0,00	1,00
**POS**	LR	RF	0,00	0,00	0,99
**POS**	LR	XGB	0,00	0,00	1,00
**POS**	SVM	RF	0,63	0,27	0,10
**POS**	SVM	XGB	0,71	0,22	0,07
**POS**	RF	XGB	0,37	0,40	0,23
**NEG**	Random	LR	0,00	0,00	1,00
**NEG**	Random	SVM	0,00	0,00	1,00
**NEG**	Random	RF	0,00	0,00	1,00
**NEG**	Random	XGB	0,00	0,00	1,00
**NEG**	LR	SVM	0,00	0,00	1,00
**NEG**	LR	RF	0,00	0,00	1,00
**NEG**	LR	XGB	0,00	0,00	1,00
**NEG**	SVM	RF	0,02	0,04	0,94
**NEG**	SVM	XGB	0,65	0,21	0,15
**NEG**	RF	XGB	0,92	0,04	0,04
**Combined**	Random	LR	0,00	0,00	1,00
**Combined**	Random	SVM	0,00	0,00	1,00
**Combined**	Random	RF	0,00	0,00	1,00
**Combined**	Random	XGB	0,00	0,00	1,00
**Combined**	LR	SVM	0,94	0,05	0,01
**Combined**	LR	RF	1,00	0,00	0,00
**Combined**	LR	XGB	0,96	0,03	0,01
**Combined**	SVM	RF	0,43	0,48	0,09
**Combined**	SVM	XGB	0,36	0,35	0,29
**Combined**	RF	XGB	0,10	0,55	0,36
**POS_vs_NEG**	LR (POS)	LR (NEG)	0,81	0,13	0,06
**POS_vs_NEG**	RF (POS)	RF (NEG)	0,06	0,06	0,88
**POS_vs_NEG**	Random (POS)	Random (NEG)	0,00	1,00	0,00

**POS_vs_NEG**	XGB (POS)	XGB (NEG)	0,41	0,39	0,20
**POS_vs_NEG**	SVM (POS)	SVM (NEG)	0,40	0,43	0,17
**Combined_vs_POS**	LR (Combined)	LR (POS)	1,00	0,00	0,00
**Combined_vs_POS**	RF (Combined)	RF (POS)	0,00	0,00	1,00
**Combined_vs_POS**	Random (Combined)	Random (POS)	0,00	1,00	0,00
**Combined_vs_POS**	XGB (Combined)	XGB (POS)	0,00	0,00	1,00
**Combined_vs_POS**	SVM (Combined)	SVM (POS)	0,01	0,01	0,98
**Combined_vs_NEG**	LR (Combined)	LR (NEG)	1,00	0,00	0,00
**Combined_vs_NEG**	RF (Combined)	RF (NEG)	0,00	0,00	0,99
**Combined_vs_NEG**	Random (Combined)	Random (NEG)	0,00	1,00	0,00
**Combined_vs_NEG**	XGB (Combined)	XGB (NEG)	0,03	0,07	0,91
**Combined_vs_NEG**	SVM (Combined)	SVM (NEG)	0,04	0,05	0,92
**POS_vs_NEG_top_models**	SVM (POS)	RF (NEG)	0,02	0,05	0,94
**POS_vs_NEG_top_models**	SVM (POS)	SVM (NEG)	0,40	0,43	0,17
**POS_vs_NEG_top_models**	RF (POS)	RF (NEG)	0,06	0,06	0,88
**POS_vs_NEG_top_models**	RF (POS)	SVM (NEG)	0,23	0,27	0,50
**POS_vs_Combined_top_models**	SVM (POS)	LR (Combined)	0,96	0,03	0,00
**POS_vs_Combined_top_models**	SVM (POS)	SVM (Combined)	0,98	0,01	0,01
**POS_vs_Combined_top_models**	RF (POS)	LR (Combined)	0,66	0,31	0,03
**POS_vs_Combined_top_models**	RF (POS)	SVM (Combined)	1,00	0,00	0,00
**NEG_vs_Combined_top_models**	RF (NEG)	LR (Combined)	0,97	0,02	0,01
**NEG_vs_Combined_top_models**	RF (NEG)	SVM (Combined)	0,97	0,01	0,01
**NEG_vs_Combined_top_models**	SVM (NEG)	LR (Combined)	0,77	0,17	0,06
**NEG_vs_Combined_top_models**	SVM (NEG)	SVM (Combined)	0,92	0,05	0,04

Flexibility

**Condition**	**Model_1**	**Model_2**	**P(Model_1 > Model_2)**	**P(Equal)**	**P(Model_2 > Model_1)**
**POS**	Random	LR	0,02	0,00	0,98
**POS**	Random	SVM	0,00	0,00	1,00
**POS**	Random	RF	0,00	0,00	1,00
**POS**	Random	XGB	0,00	0,00	1,00
**POS**	LR	SVM	0,25	0,07	0,68
**POS**	LR	RF	0,25	0,05	0,69
**POS**	LR	XGB	0,20	0,05	0,75

**POS**	SVM	RF	0,17	0,22	0,61
**POS**	SVM	XGB	0,07	0,12	0,81
**POS**	RF	XGB	0,14	0,26	0,61
**NEG**	Random	LR	0,01	0,00	0,99
**NEG**	Random	SVM	0,00	0,00	1,00
**NEG**	Random	RF	0,00	0,00	1,00
**NEG**	Random	XGB	0,00	0,00	1,00
**NEG**	LR	SVM	0,29	0,08	0,62
**NEG**	LR	RF	0,31	0,08	0,61
**NEG**	LR	XGB	0,23	0,06	0,71
**NEG**	SVM	RF	0,11	0,84	0,05
**NEG**	SVM	XGB	0,05	0,14	0,81
**NEG**	RF	XGB	0,06	0,13	0,81
**Combined**	Random	LR	0,00	0,00	1,00
**Combined**	Random	SVM	0,00	0,00	1,00
**Combined**	Random	RF	0,00	0,00	1,00
**Combined**	Random	XGB	0,00	0,00	1,00
**Combined**	LR	SVM	0,99	0,01	0,00
**Combined**	LR	RF	1,00	0,00	0,00
**Combined**	LR	XGB	0,42	0,53	0,05
**Combined**	SVM	RF	1,00	0,00	0,00
**Combined**	SVM	XGB	0,03	0,16	0,81
**Combined**	RF	XGB	0,01	0,06	0,93
**POS_vs_NEG**	Random (POS)	Random (NEG)	0,00	1,00	0,00
**POS_vs_NEG**	LR (POS)	LR (NEG)	0,32	0,12	0,56
**POS_vs_NEG**	RF (POS)	RF (NEG)	0,64	0,24	0,12
**POS_vs_NEG**	SVM (POS)	SVM (NEG)	0,29	0,36	0,35
**POS_vs_NEG**	XGB (POS)	XGB (NEG)	0,36	0,43	0,21
**Combined_vs_POS**	Random (Combined)	Random (POS)	0,00	1,00	0,00
**Combined_vs_POS**	LR (Combined)	LR (POS)	0,76	0,04	0,19
**Combined_vs_POS**	RF (Combined)	RF (POS)	0,05	0,32	0,63
**Combined_vs_POS**	SVM (Combined)	SVM (POS)	0,55	0,23	0,22
**Combined_vs_POS**	XGB (Combined)	XGB (POS)	0,17	0,74	0,09
**Combined_vs_NEG**	Random (Combined)	Random (NEG)	0,00	1,00	0,00
**Combined_vs_NEG**	LR (Combined)	LR (NEG)	0,74	0,05	0,21
**Combined_vs_NEG**	RF (Combined)	RF (NEG)	0,40	0,32	0,28
**Combined_vs_NEG**	SVM (Combined)	SVM (NEG)	0,53	0,28	0,19
**Combined_vs_NEG**	XGB (Combined)	XGB (NEG)	0,34	0,59	0,08
**POS_vs_NEG_top_models**	XGB (POS)	XGB (NEG)	0,36	0,43	0,21
**POS_vs_NEG_top_models**	XGB (POS)	SVM (NEG)	0,99	0,01	0,00
**POS_vs_NEG_top_models**	RF (POS)	XGB (NEG)	0,14	0,31	0,54

**POS_vs_NEG_top_models**	RF (POS)	SVM (NEG)	0,60	0,25	0,15
**POS_vs_Combined_top_models**	XGB (POS)	LR (Combined)	0,13	0,37	0,50
**POS_vs_Combined_top_models**	XGB (POS)	XGB (Combined)	0,09	0,74	0,17
**POS_vs_Combined_top_models**	RF (POS)	LR (Combined)	0,02	0,10	0,89
**POS_vs_Combined_top_models**	RF (POS)	XGB (Combined)	0,07	0,24	0,69
**NEG_vs_Combined_top_models**	XGB (NEG)	LR (Combined)	0,03	0,31	0,66
**NEG_vs_Combined_top_models**	XGB (NEG)	XGB (Combined)	0,08	0,59	0,34
**NEG_vs_Combined_top_models**	SVM (NEG)	LR (Combined)	0,02	0,06	0,92
**NEG_vs_Combined_top_models**	SVM (NEG)	XGB (Combined)	0,01	0,03	0,96

Postural Balance

**Condition**	**Model_1**	**Model_2**	**P(Model_1 > Model_2)**	**P(Equal)**	**P(Model_2 > Model_1)**
**POS**	Random	LR	0,00	0,00	1,00
**POS**	Random	SVM	0,00	0,00	1,00
**POS**	Random	RF	0,00	0,00	1,00
**POS**	Random	XGB	0,00	0,00	1,00
**POS**	LR	SVM	0,06	0,03	0,91
**POS**	LR	RF	0,03	0,01	0,96
**POS**	LR	XGB	0,01	0,01	0,98
**POS**	SVM	RF	0,06	0,12	0,82
**POS**	SVM	XGB	0,01	0,01	0,98
**POS**	RF	XGB	0,14	0,19	0,66
**NEG**	Random	LR	0,01	0,00	0,99
**NEG**	Random	SVM	0,00	0,00	1,00
**NEG**	Random	RF	0,00	0,00	1,00
**NEG**	Random	XGB	0,00	0,00	1,00
**NEG**	LR	SVM	0,02	0,01	0,96
**NEG**	LR	RF	0,01	0,01	0,98
**NEG**	LR	XGB	0,01	0,00	0,99
**NEG**	SVM	RF	0,16	0,22	0,62
**NEG**	SVM	XGB	0,01	0,01	0,98
**NEG**	RF	XGB	0,03	0,05	0,92
**Combined**	Random	LR	0,00	0,00	1,00
**Combined**	Random	SVM	0,00	0,00	1,00
**Combined**	Random	RF	0,00	0,00	1,00
**Combined**	Random	XGB	0,00	0,00	1,00
**Combined**	LR	SVM	0,58	0,29	0,13
**Combined**	LR	RF	0,86	0,11	0,03
**Combined**	LR	XGB	0,80	0,16	0,04

**Combined**	SVM	RF	0,64	0,32	0,04
**Combined**	SVM	XGB	0,50	0,43	0,07
**Combined**	RF	XGB	0,01	0,90	0,09
**POS_vs_NEG**	LR (POS)	LR (NEG)	0,75	0,09	0,17
**POS_vs_NEG**	SVM (POS)	SVM (NEG)	0,61	0,21	0,18
**POS_vs_NEG**	RF (POS)	RF (NEG)	0,94	0,05	0,01
**POS_vs_NEG**	Random (POS)	Random (NEG)	0,00	1,00	0,00
**POS_vs_NEG**	XGB (POS)	XGB (NEG)	0,46	0,34	0,20
**Combined_vs_POS**	LR (Combined)	LR (POS)	1,00	0,00	0,00
**Combined_vs_POS**	SVM (Combined)	SVM (POS)	0,86	0,10	0,03
**Combined_vs_POS**	RF (Combined)	RF (POS)	0,16	0,24	0,60
**Combined_vs_POS**	Random (Combined)	Random (POS)	0,00	1,00	0,00
**Combined_vs_POS**	XGB (Combined)	XGB (POS)	0,01	0,03	0,96
**Combined_vs_NEG**	LR (Combined)	LR (NEG)	0,99	0,00	0,01
**Combined_vs_NEG**	SVM (Combined)	SVM (NEG)	0,89	0,06	0,05
**Combined_vs_NEG**	RF (Combined)	RF (NEG)	0,64	0,27	0,09
**Combined_vs_NEG**	Random (Combined)	Random (NEG)	0,00	1,00	0,00
**Combined_vs_NEG**	XGB (Combined)	XGB (NEG)	0,04	0,14	0,82
**POS_vs_NEG_top_models**	XGB (POS)	XGB (NEG)	0,46	0,34	0,20
**POS_vs_NEG_top_models**	XGB (POS)	RF (NEG)	0,96	0,03	0,01
**POS_vs_NEG_top_models**	RF (POS)	XGB (NEG)	0,15	0,28	0,57
**POS_vs_NEG_top_models**	RF (POS)	RF (NEG)	0,94	0,05	0,01
**POS_vs_Combined_top_models**	XGB (POS)	LR (Combined)	0,50	0,31	0,19
**POS_vs_Combined_top_models**	XGB (POS)	SVM (Combined)	0,83	0,14	0,03
**POS_vs_Combined_top_models**	RF (POS)	LR (Combined)	0,25	0,23	0,53
**POS_vs_Combined_top_models**	RF (POS)	SVM (Combined)	0,34	0,39	0,27
**NEG_vs_Combined_top_models**	XGB (NEG)	LR (Combined)	0,28	0,51	0,20
**NEG_vs_Combined_top_models**	XGB (NEG)	SVM (Combined)	0,65	0,28	0,08
**NEG_vs_Combined_top_models**	RF (NEG)	LR (Combined)	0,06	0,08	0,86
**NEG_vs_Combined_top_models**	RF (NEG)	SVM (Combined)	0,02	0,08	0,91

Model_1: First model in comparison; Model_2: Second model in comparison; P(Model_1 > Model_2): Probability that Model_1 outperforms Model_2; P(Equal): Probability of equal performance between models; P(Model_2 > Model_1): Probability that Model_2 outperforms Model_1; POS: Positive speech condition; NEG: Negative speech condition; Combined: Combined speech data (POS+NEG); POS_vs_NEG: Performance comparison between affective conditions; Combined_vs_POS: Comparison between combined model and POS-only model; Random: Random classifier baseline; LR: Logistic Regression; SVM: Support Vector Machine; RF: Random Forest; XGB: Extreme Gradient Boosting

Supplementary Table 5 : Post Hoc pairwise comparisons of model accuracies (Nemenyi Test)

Post-hoc Nemenyi test results for pairwise comparisons of machine learning model performances across physical function domains. This non-parametric statistical test, conducted following significant Friedman tests, identifies specific model pairs with statistically significant performance differences while controlling for family-wise error rate in multiple comparisons.

Lower-Limb Endurance

POS	Random	LR	SVM	RF	XGB
**Random**	1,00	0,86	0,14	0,00	0,02
**LR**	0,86	1,00	0,69	0,05	0,27
**SVM**	0,14	0,69	1,00	0,63	0,96
**RF**	0,00	0,05	0,63	1,00	0,96
**XGB**	0,02	0,27	0,96	0,96	1,00
**NEG**	**Random**	**LR**	**SVM**	**RF**	**XGB**
**Random**	1,00	0,86	0,07	0,01	0,02
**LR**	0,86	1,00	0,50	0,12	0,27
**SVM**	0,07	0,50	1,00	0,93	0,99
**RF**	0,01	0,12	0,93	1,00	0,99
**XGB**	0,02	0,27	0,99	0,99	1,00
**Combined**	**Random**	**LR**	**SVM**	**RF**	**XGB**
**Random**	1,00	0,00	0,22	0,37	0,22
**LR**	0,00	1,00	0,32	0,18	0,32
**SVM**	0,22	0,32	1,00	1,00	1,00
**RF**	0,37	0,18	1,00	1,00	1,00
**XGB**	0,22	0,32	1,00	1,00	1,00

Lower-Limb Power

POS	Random	LR	SVM	RF	XGB
**Random**	1,00	0,05	0,00	0,37	0,56
**LR**	0,05	1,00	0,69	0,90	0,75
**SVM**	0,00	0,69	1,00	0,18	0,09
**RF**	0,04	0,90	0,18	1,00	1,00
**XGB**	0,06	0,75	0,09	1,00	1,00
**NEG**	**Random**	**LR**	**SVM**	**RF**	**XGB**
**Random**	1,00	0,00	0,09	0,43	0,22
**LR**	0,00	1,00	0,75	0,27	0,50
**SVM**	0,09	0,75	1,00	0,93	0,99
**RF**	0,43	0,27	0,93	1,00	0,99
**XGB**	0,22	0,50	0,99	0,99	1,00
**Combined**	**Random**	**LR**	**SVM**	**RF**	**XGB**
**Random**	1,00	0,56	0,56	0,04	0,01
**LR**	0,56	1,00	1,00	0,69	0,43

**SVM**	0,56	1,00	1,00	0,69	0,43
**RF**	0,04	0,69	0,69	1,00	0,99
**XGB**	0,01	0,43	0,43	0,99	1,00

Lower-Limb Strength

**POS**	**Random**	**LR**	**SVM**	**RF**	**XGB**
**Random**	1,00	0,81	0,32	0,00	0,01
**LR**	0,81	1,00	0,93	0,07	0,18
**SVM**	0,32	0,93	1,00	0,37	0,63
**RF**	0,00	0,07	0,37	1,00	0,99
**XGB**	0,01	0,18	0,63	0,99	1,00
**NEG**	**Random**	**LR**	**SVM**	**RF**	**XGB**
**Random**	1,00	0,75	0,32	0,00	0,01
**LR**	0,75	1,00	0,96	0,09	0,27
**SVM**	0,32	0,96	1,00	0,37	0,69
**RF**	0,00	0,09	0,37	1,00	0,99
**XGB**	0,01	0,27	0,69	0,99	1,00
**Combined**	**Random**	**LR**	**SVM**	**RF**	**XGB**
**Random**	1,00	0,01	0,37	0,22	0,03
**LR**	0,01	1,00	0,63	0,81	1,00
**SVM**	0,37	0,63	1,00	1,00	0,81
**RF**	0,22	0,81	1,00	1,00	0,93
**XGB**	0,03	1,00	0,81	0,93	1,00

Grip Strength

**POS**	**Random**	**LR**	**SVM**	**RF**	**XGB**
**Random**	1,00	0,86	0,22	0,00	0,03
**LR**	0,86	1,00	0,81	0,02	0,32
**SVM**	0,22	0,81	1,00	0,32	0,93
**RF**	0,00	0,02	0,32	1,00	0,81
**XGB**	0,03	0,32	0,93	0,81	1,00
**NEG**	**Random**	**LR**	**SVM**	**RF**	**XGB**
**Random**	1,00	0,86	0,04	0,01	0,02
**LR**	0,86	1,00	0,37	0,18	0,27
**SVM**	0,04	0,37	1,00	0,99	1,00
**RF**	0,01	0,18	0,99	1,00	1,00
**XGB**	0,02	0,27	1,00	1,00	1,00
**Combined**	**Random**	**LR**	**SVM**	**RF**	**XGB**
**Random**	1,00	0,86	0,00	0,12	0,07
**LR**	0,86	1,00	0,02	0,63	0,50
**SVM**	0,00	0,02	1,00	0,50	0,63
**RF**	0,12	0,63	0,50	1,00	1,00
**XGB**	0,07	0,50	0,63	1,00	1,00

Walk speed

POS	Random	LR	SVM	RF	XGB
**Random**	1,00	0,75	0,27	0,00	0,02
**LR**	0,75	1,00	0,93	0,07	0,37
**SVM**	0,27	0,93	1,00	0,37	0,86
**RF**	0,00	0,07	0,37	1,00	0,93
**XGB**	0,02	0,37	0,86	0,93	1,00
**NEG**	**Random**	**LR**	**SVM**	**RF**	**XGB**
**Random**	1,00	0,69	0,43	0,00	0,02
**LR**	0,69	1,00	0,99	0,07	0,37
**SVM**	0,43	0,99	1,00	0,18	0,63
**RF**	0,00	0,07	0,18	1,00	0,93
**XGB**	0,02	0,37	0,63	0,93	1,00
**Combined**	**Random**	**LR**	**SVM**	**RF**	**XGB**
**Random**	1,00	0,86	0,27	0,00	0,01
**LR**	0,86	1,00	0,86	0,04	0,18
**SVM**	0,27	0,86	1,00	0,37	0,75
**RF**	0,00	0,04	0,37	1,00	0,98
**XGB**	0,01	0,18	0,75	0,98	1,00

Mobility

POS	Random	LR	SVM	RF	XGB
**Random**	1,00	0,75	0,02	0,14	0,01
**LR**	0,75	1,00	0,32	0,81	0,18
**SVM**	0,02	0,32	1,00	0,93	1,00
**RF**	0,14	0,81	0,93	1,00	0,81
**XGB**	0,01	0,18	1,00	0,81	1,00
**NEG**	**Random**	**LR**	**SVM**	**RF**	**XGB**
**Random**	1,00	0,69	0,09	0,02	0,01
**LR**	0,69	1,00	0,75	0,43	0,32
**SVM**	0,09	0,75	1,00	0,99	0,96
**RF**	0,02	0,43	0,99	1,00	1,00
**XGB**	0,01	0,32	0,96	1,00	1,00
**Combined**	**Random**	**LR**	**SVM**	**RF**	**XGB**
**Random**	1,00	0,86	0,27	0,02	0,00
**LR**	0,86	1,00	0,86	0,27	0,02
**SVM**	0,27	0,86	1,00	0,86	0,27
**RF**	0,02	0,27	0,86	1,00	0,86
**XGB**	0,00	0,02	0,27	0,86	1,00

Lean Appendicular Mass

POS	Random	LR	SVM	RF	XGB
**Random**	1,00	0,43	0,50	0,00	0,03
**LR**	0,43	1,00	1,00	0,22	0,75
**SVM**	0,50	1,00	1,00	0,18	0,69

RF	0,00	0,22	0,18	1,00	0,90
**XGB**	0,03	0,75	0,69	0,90	1,00
**NEG**	**Random**	**LR**	**SVM**	**RF**	**XGB**
**Random**	1,00	0,75	0,37	0,00	0,01
**LR**	0,75	1,00	0,98	0,07	0,27
**SVM**	0,37	0,98	1,00	0,27	0,63
**RF**	0,00	0,07	0,27	1,00	0,98
**XGB**	0,01	0,27	0,63	0,98	1,00
**Combined**	**Random**	**LR**	**SVM**	**RF**	**XGB**
**Random**	1,00	0,07	0,37	0,01	0,14
**LR**	0,07	1,00	0,93	0,96	1,00
**SVM**	0,37	0,93	1,00	0,56	0,99
**RF**	0,01	0,96	0,56	1,00	0,86
**XGB**	0,14	1,00	0,99	0,86	1,00

Fatigue

POS	Random	LR	SVM	RF	XGB
**Random**	1,00	0,86	0,00	0,05	0,05
**LR**	0,86	1,00	0,07	0,43	0,43
**SVM**	0,00	0,07	1,00	0,90	0,90
**RF**	0,05	0,43	0,90	1,00	1,00
**XGB**	0,05	0,43	0,90	1,00	1,00
**NEG**	**Random**	**LR**	**SVM**	**RF**	**XGB**
**Random**	1,00	0,86	0,07	0,00	0,07
**LR**	0,86	1,00	0,50	0,04	0,50
**SVM**	0,07	0,50	1,00	0,75	1,00
**RF**	0,00	0,04	0,75	1,00	0,75
**XGB**	0,07	0,50	1,00	0,75	1,00
**Combined**	**Random**	**LR**	**SVM**	**RF**	**XGB**
**Random**	1,00	0,00	0,18	0,37	0,27
**LR**	0,00	1,00	0,37	0,18	0,27
**SVM**	0,18	0,37	1,00	0,99	1,00
**RF**	0,37	0,18	0,99	1,00	1,00
**XGB**	0,27	0,27	1,00	1,00	1,00

Flexibility

POS	Random	LR	SVM	RF	XGB
**Random**	1,00	0,04	0,18	0,22	0,03
**LR**	0,04	1,00	0,98	0,96	1,00
**SVM**	0,18	0,98	1,00	1,00	0,96
**RF**	0,22	0,96	1,00	1,00	0,93
**XGB**	0,03	1,00	0,96	0,93	1,00
**NEG**	**Random**	**LR**	**SVM**	**RF**	**XGB**
**Random**	1,00	0,04	0,22	0,27	0,02
**LR**	0,04	1,00	0,96	0,93	1,00

**SVM**	0,22	0,96	1,00	1,00	0,86
**RF**	0,27	0,93	1,00	1,00	0,81
**XGB**	0,02	1,00	0,86	0,81	1,00
**Combined**	**Random**	**LR**	**SVM**	**RF**	**XGB**
**Random**	1,00	0,00	0,18	0,81	0,02
**LR**	0,00	1,00	0,56	0,07	0,96
**SVM**	0,18	0,56	1,00	0,81	0,93
**RF**	0,81	0,07	0,81	1,00	0,32
**XGB**	0,02	0,96	0,93	0,32	1,00

Postural Balance

**POS**	**Random**	**LR**	**SVM**	**RF**	**XGB**
**Random**	1,00	0,75	0,27	0,01	0,00
**LR**	0,75	1,00	0,93	0,27	0,12
**SVM**	0,27	0,93	1,00	0,75	0,50
**RF**	0,01	0,27	0,75	1,00	0,99
**XGB**	0,00	0,12	0,50	0,99	1,00
**NEG**	**Random**	**LR**	**SVM**	**RF**	**XGB**
**Random**	1,00	0,86	0,14	0,05	0,00
**LR**	0,86	1,00	0,69	0,43	0,02
**SVM**	0,14	0,69	1,00	0,99	0,43
**RF**	0,05	0,43	0,99	1,00	0,69
**XGB**	0,00	0,02	0,43	0,69	1,00
**Combined**	**Random**	**LR**	**SVM**	**RF**	**XGB**
**Random**	1,00	0,01	0,04	0,50	0,18
**LR**	0,01	1,00	0,98	0,37	0,75
**SVM**	0,04	0,98	1,00	0,75	0,98
**RF**	0,50	0,37	0,75	1,00	0,98
**XGB**	0,18	0,75	0,98	0,98	1,00

POS: Positive speech condition; NEG: Negative speech condition; Combined: Combined speech data (POS + NEG); Random: Random classifier baseline; LR: Logistic Regression; SVM: Support Vector Machine; RF: Random Forest; XGB: Extreme Gradient Boosting; Matrix values: Bayesian probability that row model outperforms column model

Supplementary Table 6 : Normative ranges and impairment thresholds for physical function measures

Function	Calculation method	Age Group	Men (Mean ± SD)	Deficit Threshold (Men)	Women (Mean ± SD)	Deficit Threshold (Women)	References
**Walk speed (m/s)**	Direct measurement (m/s)	60–69	1.40 ± 0.18	<1.22	1.36 ± 0.23	<1.13	1
		70–79	1.19 ± 0.18	<1.01	1.11 ± 0.15	<0.96	1, 2
		80–89	1.10 ± 0.23	<0.87	0.98 ± 0.18	<0.80	1, 2
**6MWT (m)**	Direct measurement (m)	60–69	560 ± 49	<511	505 ± 45	<460	3, 4, 5
		70–79	530 ± 48	<482	490 ± 48	<442	3
		80–89	446 ± 61	<385	382 ± 66	<316	3
**LAM (kg/m²)**	Direct measurement (kg/m²)	60–69	7.8 ± 0.9	<7.0	6.3 ± 0.8	<5.5	6
		70–79	7.2 ± 1.1	<7.0	5.8 ± 1.0	<5.5	6
		80+	6.5 ± 1.2	<7.0	5.2 ± 1.1	<5.5	7
**Fatigue (MFI)**	Total score from questionnaire	All ages	NA	>36	NA	>36	8
**Balance (sec)**	Max standing time (sec)	60+	34 ± 12	<22	30 ± 10	<20	9, 10, 11
**Flexibility (cm)**	Direct measurement on a -30 to +30 scale (cm)	60–69	-7.6 to +7.6	<5.0	-1.3 to +11.4	<7.0	12
		70–74	-8.9 to +6.4	<4.0	-2.5 to +10.2	<6.0	13
		75–79	-10.2 to +5.1	<3.0	N/A	<5.0	14
**Strength (Nm)**	Normalized Force (%) = (Measured Peak Torque / Predicted Peak Torque) × 100	60+	140 ± 20	<120	85 ± 15	<70	15, 16
**Power (W)**	Normalized Power (%) = (Measured Power / Predicted Power) × 100	60+	200 ± 50	<150	115 ± 35	<80	17
**Endurance (J)**	Normalized Endurance (%) = (Measured Work / Predicted Work) × 100	60+	2000 ± 500	<1500	1350 ± 450	<900	18
**Hand grip (N)**	Direct measurement (N)	60–69	392 ± 78	<314	235 ± 49	<186	19
		70–79	324 ± 59	<265	196 ± 39	<157	19
		80+	270 ± 49	<221	186 ± 29	<157	19

For all assessments except Biodex measurements, deficit was defined as performance ≥1 standard deviation below age- and sex-specific normative means derived from population-based studies. For Biodex dynamometric measurements, deficit was defined as normalized performance <90% of predicted values based on manufacturer specifications and clinical guidelines. Predicted values for Biodex measurements are reference norms calculated according to age and sex. The -1 SD threshold is widely accepted in clinical research as indicative of mild functional impairment enabling early intervention.

References

1. Lee, S. et al. Age-specific normative values for walking speed in older adults: A systematic review. BMC Geriatr 21, 111 (2021).
2. Studenski, S. et al. Gait speed and survival in older adults. JAMA 305, 50–58 (2011).
3. Enright, P.L. & Sherrill, D.L. Reference equations for the six-minute walk in healthy adults. Am J Respir Crit Care Med 158, 1384–1387 (1998).
4. Rikli, R.E. & Jones, C.J. Functional fitness normative scores for community-residing older adults. J Aging Phys Act 6, 127–159 (1998).
5. American Thoracic Society. ATS statement: guidelines for the six-minute walk test. Am J Respir Crit Care Med 166, 111–117 (2002).
6. Cruz-Jentoft, A.J. et al. Sarcopenia: revised European consensus on definition and diagnosis. Age Ageing 48, 16–31 (2019).
7. Santilli, V. et al. Sarcopenia in older people: a preliminary cross-sectional study. Aging Clin Exp Res 32, 159–165 (2020).
8. Smets, E.M.A. et al. The Multidimensional Fatigue Inventory (MFI) psychometric qualities of an instrument to assess fatigue. J Psychosom Res 39, 315–325 (1995).
9. Bohannon, R.W. et al. Standing balance and strength measurements in older adults. Phys Ther 64, 191–195 (1984).
10. National Institutes of Health. NIH Toolbox Balance Norms (2017).
11. Missouri University Balance Toolkit. Standardized tests for fall risk screening (2019).
12. Rezvani, M. et al. The validity of spinal flexibility measures in older adults. Spine 37, 123–129 (2012).
13. Yen, C.J. et al. Normative values of trunk flexibility in older adults. Biomed Res Int 2015, 153909 (2015).
14. Kim, H. et al. Age-related changes in spinal flexibility and its association with frailty. PMC4530222 (2015).
15. Bijlsma, A.Y. et al. Muscle strength as a predictor of fall risk in older adults. BMC Musculoskelet Disord 21, 633 (2020).
16. Sallinen, J. et al. Hand-grip strength cut points to screen older persons at risk for mobility limitation. SAGE Open Med 9, 20503121211004300 (2021).
17. Lindemann, U. et al. Power and stair ascent performance in older adults. Front Bioeng Biotechnol 13, 100835 (2023).
18. Müller, V. et al. Isokinetic muscle endurance and functional mobility in the elderly. Front Physiol 12, 768907 (2021).
19. Dodds, R.M. et al. Grip strength across the life course: normative data from twelve British studies. PLoS One 9, e113637 (2014).
20. Biodex Medical Systems. System 4 Quick-Start Operating Manual. Biodex Inc., Shirley, NY (2017).
21. Cesari, M. et al. Prognostic value of usual gait speed in well-functioning older people—results from the Health, Aging and Body Composition Study. J. Am. Geriatr. Soc. 55, 266–271 (2007).
22. Middleton, A., Fritz, S. L. & Lusardi, M. Walking speed: the functional vital sign. J. Aging Phys. Act. 23, 314–322 (2015).

Supplementary Table 7 : Acoustic (a), Linguistic (b), and Temporal (c) speech markers with related extraction parameters

Detailed specification of the tripartite speech feature extraction framework comprising (a) acoustic, (b) temporal, and (c) linguistic markers with corresponding extraction parameters, computational methods, and clinical rationales. This comprehensive feature engineering protocol enables multi-dimensional characterization of speech production mechanisms relevant to physical function assessment.

Acoustic Features

Feature name	Definition	Extraction Parameters
Mean pitch	Average fundamental frequency (F0)	Extracted using Librosa v0.10.2, 25 ms Hamming window, 10 ms hop length
Absolute jitter	Mean absolute difference between consecutive pitch periods	Calculated from pitch periods using Librosa, 25 ms window
Jitter PPQ5	Smoothed local jitter over five consecutive periods	Computed using 5-period windows from pitch contour
Shimmer (dB)	Cycle-to-cycle variability in amplitude	Derived from amplitude envelope, 25 ms analysis window
Shimmer APQ5	Amplitude perturbation quotient over 5 cycles	Calculated using 5-cycle amplitude windows
Median pitch	Median of the pitch contour	From F0 contour, 25 ms window, 10 ms hop
Minimum pitch	Lowest detected F0	Minimum of F0 contour across entire recording
Maximum pitch	Highest detected F0	Maximum of F0 contour across entire recording
Number of pitch periods	Total number of voiced cycles	Count of voiced frames in F0 contour
Minimum intensity	Lowest detected vocal intensity (dB SPL)	From RMS energy, 25 ms window
Maximum intensity	Highest detected vocal intensity	Maximum RMS energy across recording
Mean intensity	Average speech intensity	Mean RMS energy, 25 ms window, 10 ms hop
MFCCs 1–8 (mean)	Average of first 8 Mel-Frequency Cepstral Coefficients	25 ms Hamming window, 10 ms hop, 128 mel bands, 50-8000 Hz range
Mean chroma	Average chroma energy across 12 pitch classes	25 ms window, 10 ms hop, 12 chroma bins
Mean spectral contrast	Difference in amplitude between spectral peaks and valleys	25 ms window, 10 ms hop, 6 frequency bands
Mean zero-crossing rate	Average zero-crossings per second	25 ms window, 10 ms hop
Mean harmonics-to-noise ratio (HNR)	Ratio of harmonic to aperiodic energy	Calculated using autocorrelation method, 25 ms window
Pitch slope	Overall slope of pitch contour	Linear regression slope of F0 contour over time
First Fourier coefficient of pitch	Dominant frequency component of pitch contour	FFT on F0 contour, 25 ms window

Temporal Features

Feature name	Definition	Extraction Parameters
**Total speech duration**	Length of spoken segment (seconds)	From Montreal Forced Aligner v3.2.1 alignment, French dictionary
**Total number of words**	Count of all spoken words	Montreal Forced Aligner alignment with Whisper v3 transcription
**Speech rate (words/s)**	Words spoken per second	Words count divided by speech duration
**Phoneme rate**	Phonemes uttered per second	Montreal Forced Aligner phoneme alignment, phoneme count/duration
**Syllable rate**	Syllables spoken per second	Estimated from phoneme sequence
**Mean word duration**	Average duration of words	Montreal Forced Aligner word-level alignment

**Mean word length (in phonemes)**	Average phonemes per word	Phoneme count from Montreal Forced Aligner alignment
**Longest word duration**	Duration of longest word	Maximum from Montreal Forced Aligner word durations
**Longest word (in phonemes)**	Phoneme count of longest word	From Montreal Forced Aligner phoneme alignment
**Number of pauses**	Count of silent pauses	Silence >100 ms from Montreal Forced Aligner alignment
**Total pause duration**	Cumulative duration of silent intervals	Sum of all pause durations from Montreal Forced Aligner
**Mean pause duration**	Average duration per silent pause	Total pause duration / number of pauses
**Longest pause duration**	Duration of longest silence	Maximum pause duration from Montreal Forced Aligner
**Mean vowel duration**	Average duration of vowel segments	From Montreal Forced Aligner phoneme alignment, vowel categories
**Mean plosive duration**	Average duration of plosive consonants	Montreal Forced Aligner alignment, plosive phoneme categories
**Mean fricative duration**	Average duration of fricative consonants	Montreal Forced Aligner alignment, fricative phoneme categories
**Mean nasal duration**	Mean duration of nasal consonants	Montreal Forced Aligner alignment, nasal phoneme categories
**Mean liquid duration**	Mean duration of liquids	Montreal Forced Aligner alignment, liquid phoneme categories
**Mean glide duration**	Duration of glide consonants	Montreal Forced Aligner alignment, glide phoneme categories
**Total number of phones**	Total number of phonetic units	Phoneme count from Montreal Forced Aligner alignment

Linguistic Features

**Feature name**	**Definition**	**Extraction Parameters**
**Verb ratio**	Proportion of verbs among all words	spaCy v3.8.2 French model, part-of-speech tagging
**Verb rate**	Number of verbs spoken per second	Verb count divided by speech duration
**Noun ratio**	Proportion of nouns in speech	spaCy part-of-speech tagging, noun identification
**Noun rate**	Number of nouns spoken per second	Noun count divided by speech duration
**Pronoun ratio**	Proportion of pronouns	spaCy part-of-speech tagging, pronoun categories
**Pronoun rate**	Number of pronouns spoken per second	Pronoun count divided by speech duration
**Article ratio**	Proportion of articles	spaCy part-of-speech tagging, article identification
**Article rate**	Number of articles per second	Article count divided by speech duration
**Adjective ratio**	Proportion of adjectives	spaCy part-of-speech tagging, adjective identification
**Adjective rate**	Number of adjectives per second	Adjective count divided by speech duration
**Adverb ratio**	Share of adverbs	spaCy part-of-speech tagging, adverb identification
**Adverb rate**	Adverbs spoken per second	Adverb count divided by speech duration
**Preposition ratio**	Proportion of prepositions	spaCy part-of-speech tagging, preposition identification
**Preposition rate**	Number of prepositions per second	Preposition count divided by speech duration
**Conjunction ratio**	Proportion of conjunctions	spaCy part-of-speech tagging, conjunction identification
**Conjunction rate**	Rate of conjunctions used per second	Conjunction count divided by speech duration

**Interjection ratio**	Share of interjections	spaCy part-of-speech tagging, interjection identification
**Interjection rate**	Rate of interjection usage	Interjection count divided by speech duration
**Time-related word ratio**	Proportion of time-referencing words	Custom lexicon matching, spaCy tokens
**Time-related word rate**	Rate of time-referencing words per second	Time word count divided by speech duration
**Narrator-related word ratio**	Proportion of personal agency words	Custom lexicon (I, me, my), spaCy tokens
**Narrator-related word rate**	Rate of narrator-related words per second	Narrator word count divided by speech duration
**Positive emotion word ratio**	Share of positive emotion words	Custom emotion lexicon, spaCy token matching
**Positive emotion word rate**	Positive emotional words per second	Positive emotion count divided by duration
**Negative emotion word ratio**	Proportion of negatively valenced words	Custom emotion lexicon, spaCy token matching
**Negative emotion word rate**	Rate of negatively valenced words per second	Negative emotion count divided by duration
**Location word ratio**	Proportion of location-referencing words	Custom spatial lexicon, spaCy token matching
**Location word rate**	Frequency of location-referencing words	Location word count divided by speech duration
